# Metabolite glues as a means of purine sensing and chemotherapeutic response

**DOI:** 10.1038/s41586-026-10790-3

**Published:** 2026-07-15

**Authors:** Samuel R. Witus, Megan M. Kober, Heegwang Roh, Zhi Yang, Fouad Choueiry, Avani S. Ghate, Denis V. Titov, Michael Rapé

**Affiliations:** 1 Department of Molecular and Cell Biology, University of California, Berkeley, Berkeley, CA USA; 2 Department of Nutritional Sciences and Toxicology, University of California, Berkeley, Berkeley, CA USA; 3 Howard Hughes Medical Institute, University of California, Berkeley, Berkeley, CA USA; 4 Center for Computational Biology, University of California, Berkeley, Berkeley, CA USA; 5 California Institute for Quantitative Biosciences (QB3), University of California, Berkeley, Berkeley, CA USA

**Keywords:** Cryoelectron microscopy, Nutrient signalling

## Abstract

Molecular glues stabilize weak interactions to impart new functionalities to complexes^1–3^. Although molecular glues have been described in plant signalling and as human therapeutics^4,5^, it is unclear whether this modality provides endogenous regulation in human cells. Here we show that purine nucleotides are molecular glues that tether the rate-limiting enzyme in purine biosynthesis—phosphoribosyl pyrophosphate amidotransferase (PPAT)—to its inhibitor NUDT5. This mechanism allows cells to sense the levels of purines and to establish essential feedback control of their synthesis. We refer to such molecules as metabolite glues. Thiopurine chemotherapeutics^6^, which have been in clinical use since the 1950s, glue the same complex but adopt distinct orientations for enhanced function. Unlike most known glues, the PPAT–NUDT5 metabolite-glue pocket can adjust its conformation to notable compound alterations, enabling increased glue potency and improved on-target activity. We therefore identify endogenous metabolite glues as a mode of nutrient sensing that can be exploited for therapeutic benefit.

## Abstract (web summary)

Purines can act as endogenous molecular glues in human cells by anchoring a rate-limiting enzyme in purine biosynthesis to its inhibitor, and modifying this mechanism could provide a way to improve the properties of thiopurine chemotherapeutic drugs.

## Main

The ability of cells to synthesize distinct metabolites from shared precursors is essential for tissue formation and homeostasis, and the biosynthetic pathways responsible must adapt rapidly to the frequent shifts in metabolic demand that occur during proliferation, differentiation or stress. This requires regulatory circuits that continuously monitor and adjust the levels of metabolic precursors and products^7,8^. Loss of nutrient sensing and feedback control accounts for some systemic diseases, such as diabetes, and for developmental pathologies—as seen in patients with mutations in *PRPS1* or Lesch–Nyhan syndrome, which stem from dysregulated purine metabolism^9^.

De novo purine biosynthesis is among the most crucial and energetically demanding metabolic pathways. It uses ATP, amino acids, mitochondrially derived cofactors and ribose-5-phosphate to generate nucleotides that fuel proliferation, nucleic acid synthesis, signalling and bioenergetics^10^. To prevent excess consumption or misallocation of metabolic building blocks, de novo purine synthesis is regulated by end-product inhibition^11,12^. Conversely, high purine demand stimulates production through allosteric enzyme activation, formation of a multi-enzyme purinosome, assembly of enzyme filaments and mTORC1 or RAS–ERK signalling^13–19^. This is complemented by a salvage pathway that recycles nucleobases at reduced energetic cost^20^. Isotope tracing has shown that most tissues rely on both de novo and salvage pathways^21^, suggesting a need for coordination that remains incompletely understood. Although enzymes of purine synthesis are conserved across all kingdoms of life, much of what is known about their regulation is inferred from studies in bacteria. It is likely that additional complexity evolved to accommodate the unique metabolic requirements of metazoan organisms.

Reflecting its importance for cell survival, purine metabolism has been widely exploited for the treatment of cancer, autoimmune disorders and organ transplant rejection^22^. Therapeutics that target purine metabolism were introduced into the clinic in the 1940s, including the antifolates aminopterin or methotrexate (MTX) and the thiopurine 6-mercaptopurine (6-MP), which remain staples of cancer treatment today^23,24^. Whereas MTX depletes metabolic cofactors that are required for purine synthesis, 6-MP is thought to act, at least in part, by being incorporated into DNA. Both drugs induce DNA damage to impart cancer cell death, but show off-target toxicity that limits therapeutic efficiency. Because purine availability constrains cell proliferation and immune activation, identifying regulatory mechanisms that sense and adjust purine biosynthesis could reveal strategies to exploit this process for cancer treatments and immunosuppressive therapies.

Here we show that de novo purine synthesis is regulated by endogenous molecular glues that we refer to as metabolite glues. Structural studies of the rate-limiting enzyme in purine synthesis, PPAT, reveal that purine monophosphates anchor the PPAT inhibitor NUDT5 in the active site of PPAT. This mechanism enables cells to monitor purine levels and provides essential feedback control for purine biosynthesis. Thiopurine chemotherapeutics act as molecular glues of the same complex, but adopt altered binding modes at the protein interface that translate conformational changes in PPAT into glues with increased potency. Of note, we could exploit the structural flexibility of this glue pocket to increase thiopurine potency and on-target activity. Our study therefore identifies metabolite glues as a mode of nutrient sensing and provides a starting point for the rational discovery of therapeutically relevant molecular glues.

## NUDT5 regulates purine synthesis

**Fig. 1:**
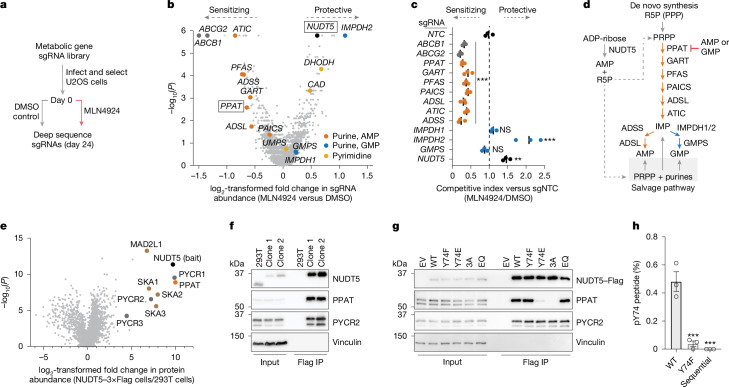
NUDT5 regulates purine metabolism by interacting with PPAT. **a**, Schematic of the metabolism-focused CRISPR screen. **b**, Comparison of the sgRNA abundance of metabolic genes on day 24 of the screen between MLN4924-treated and dimethyl sulfoxide (DMSO)-treated cells. Symbol colours denote pathway classifications: core de novo purine synthesis and adenylate production (orange), guanylate production (blue) and de novo pyrimidine synthesis (yellow). *ABCB1* and *ABCG2* (grey) encode multidrug efflux channels. Colour coding is consistent across **b**–**d**. *ADSS* is also known as *ADSS2*. **c**, Validation of the MLN4924 sensitivity of U2OS cells with selected gene knockdowns through a flow cytometry (fluorescence-activated cell sorting; FACS)-based cell competition experiment. Data are mean and individual values from *n* = 3 biological replicates. NTC, non-targeting control. **d**, Schematic of de novo purine biosynthesis, purine salvage and NUDT5 ADP-ribose hydrolase activity. R5P, ribose-5-phosphate; PPP, pentose phosphate pathway. **e**, Mass spectrometry proteomics of NUDT5–3×Flag immunoprecipitation from HEK293T cells. Statistical data are derived from *n* = 3 technical replicates. **f**, Western blot of Flag immunoprecipitation (IP) from HEK293T cells expressing NUDT5–3×Flag. Blot is representative of two independent experiments. **g**, Western blot of Flag immunoprecipitation from *∆NUDT5* HEK293T cells expressing wild-type (WT) NUDT5–3×Flag and mutants. EV, empty vector. Blot is representative of two independent experiments. **h**, Abundance of phosphorylated (p)Y74 peptide in NUDT5 from single NUDT5 (WT and Y74F) and PPAT–NUDT5 sequential immunoprecipitations from HEK293T cells. Data are mean ± s.e.m. with individual values from *n* = 3 technical replicates. Statistical significance in **c**,**h** was determined using a one-way ANOVA followed by Dunnett’s multiple-comparisons test against the control condition. NS, not significant. **P < 0.01 and ***P < 0.001. Exact *P* values are included as source data. Source data

**Extended Data Fig. 1:**
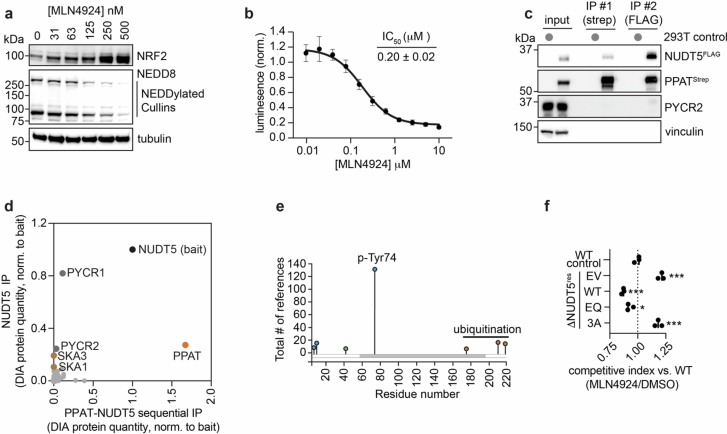
Identification of the PPAT–NUDT5 complex. **a**. Western blot of U2OS cells treated with indicated amounts of MLN4924 for 16 h. Blot is representative of two independent experiments. **b**. Growth of U2OS cells treated with MLN4924 for 72 h. Data represent the mean ± s.e.m. from n = 3 independent biological replicates. **c**. Western blot of sequential immunoprecipitation from HEK293T cells expressing PPAT–Strep and NUDT5–3×Flag. **d**. Mass spectrometry of PPAT–Strep–NUDT5–3×Flag sequential immunoprecipitation compared to NUDT5–3×Flag-only immunoprecipitation and normalized to NUDT5 bait protein abundance. Data represent the mean of n = 3 technical replicates. **e**. Survey of NUDT5 post-translational modifications from the PhosphoSitePlus database filtered for n > 5 observations. **f**. FACS-based growth competition comparing Δ*NUDT5* and rescue mutants to wild-type HEK293T cells treated with MLN4924. Data represent the mean with individual values from n = 3 biological replicates. Statistical significance was assessed using one-way ANOVA followed by Dunnett’s multiple-comparisons test against wild-type control. Significance is indicated as ns (not significant), *P < 0.05, **P < 0.01 and ***P < 0.001. Exact *P* values are included as source data. Source data

Because cullin–RING E3 ligases (CRLs) have emerged as crucial regulators of biosynthetic pathways^25–28^, we hypothesized that modulating their activity could allow us to discover mechanisms of metabolic control. We therefore sensitized osteosarcoma cells with sublethal doses of the CRL inhibitor MLN4924, which prevents neddylation of Cullin scaffolds, and performed a genetic screen using an single guide RNA (sgRNA) library targeting around 3,000 metabolic enzymes and transporters to identify chemogenetic interactions (Fig. 1a and Extended Data Fig. 1a,b). We found that cells depleted of enzymes involved in de novo purine synthesis—specifically, those enzymes that lead to adenylate production—were more sensitive to partial CRL inhibition than were control cells (Fig. 1b–d). CRLs have previously been implicated in the control of purine synthesis through regulation of IMPDH2, purinosome formation, nucleobase transport, glycolysis or mitochondrial function^25,27–29^. Conversely, cells depleted of enzymes involved in de novo pyrimidine synthesis, which compete with purine biosynthesis enzymes for shared precursors, were more resistant to CRL inhibition. Similar to targeting the pyrimidine pathway, loss of the NUDT5 protein was protective against MLN4924 treatment (Fig. 1b–d). NUDT5 is a Nudix hydrolase that cleaves ADP-ribose into AMP and ribose-5-phosphate, and synthesizes nuclear ATP to facilitate chromatin remodelling and DNA repair^30,31^, but how it regulates the response to CRL inhibition was unknown.

To reveal how NUDT5 functions in MLN4924-treated cells, we identified its interactors by affinity purification and mass spectrometry. NUDT5 co-immunoprecipitated with the rate-limiting enzyme of de novo purine biosynthesis, PPAT, depletion of which sensitized cells to MLN4924 (Fig. 1e,f). NUDT5 also bound to the mitochondrial proline biosynthetic enzymes PYCR1 and PYCR2 and to components of the kinetochore-associated SKA complex. Sequential immunoprecipitation of PPAT and NUDT5 showed that these proteins form a discrete complex that does not include PYCR1, PYCR2 or SKA subunits (Extended Data Fig. 1c,d).

**Extended Data Fig. 2:**
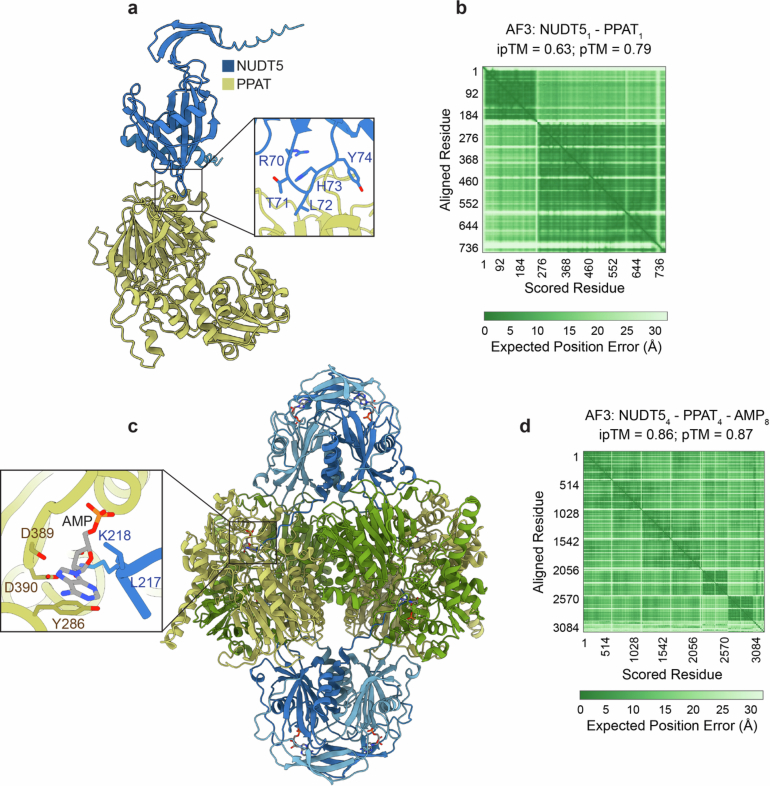
AlphaFold modelling of PPAT–NUDT5. **a**. AlphaFold3 model of PPAT–NUDT5 monomer interaction. **b**. Contact map for structure prediction shown in **a**. **c**. AlphaFold3 model of PPAT–NUDT5 hetero-octameric complex with 8 molecules of AMP, highlighting the predicted metabolite-glue interface. **d**. Contact map for structure prediction shown in **c**.

AlphaFold modelling^32^ suggested that NUDT5 recognizes PPAT through its Y74 residue, which can be phosphorylated in cells (Extended Data Figs. 1e and 2a,b). Although a Y74F mutation did not impede binding to PPAT, the phosphomimetic NUDT5(Y74E) or a more drastic loop mutation, NUDT5(R70A/L72A/Y74A) (hereafter, NUDT5(3A)), disrupted this interaction (Fig. 1g), and introducing NUDT5(3A) into Δ*NUDT5* cells did not restore MLN4924 sensitivity (Extended Data Fig. 1f). Consistent with these observations, we did not find any phosphorylated NUDT5 in complexes with PPAT, despite efficient detection of this modification in NUDT5 affinity purifications (Fig. 1h) By contrast, a catalytic mutant defective in nucleotide hydrolysis, NUDT5(E112Q/E116Q) (hereafter, NUDT5(EQ); ref. ^30^), still bound to PPAT (Fig. 1g) and re-established MLN4924 sensitivity in Δ*NUDT5* cells (Extended Data Fig. 1f). These data show that NUDT5 controls drug sensitivity through PPAT, but independently of its role as a hydrolase. Our findings are consistent with previous studies that implicated NUDT5 as a regulator of PPAT, but did not identify the molecular basis for this activity^33–37^.

## Structure of the PPAT–NUDT5–AMP complex

**Fig. 2:**
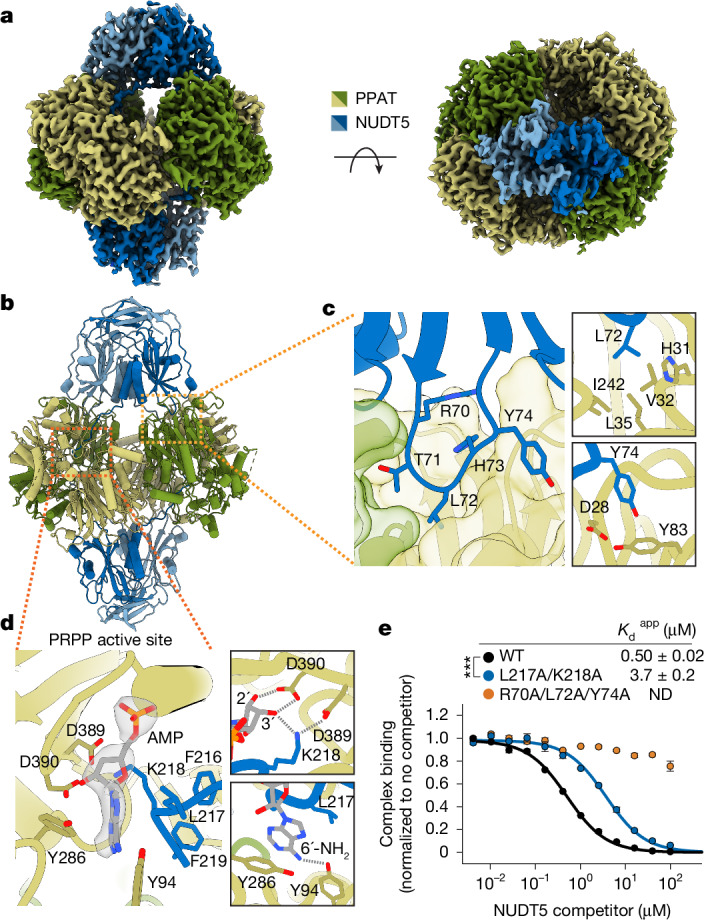
Structure of the PPAT–NUDT5–AMP complex. **a**, Cryo-EM density of the PPAT–NUDT5–AMP complex. **b**, Structural model of the PPAT–NUDT5–AMP complex. The light-orange box shows the location of the R70–Y74 loop interface, and the dark-orange box indicates the positioning of the molecular-glue interface in the same NUDT5 protomer. **c**, The R70–Y74 loop interface, highlighting specific interactions between NUDT5 and PPAT including the pY74 site. The Phe74 residue used to stabilize the complex for structure determination was replaced with the native Tyr in the model shown. **d**, The PPAT–NUDT5–AMP molecular-glue interface. Cryo-EM density for AMP is shown as a transparent surface. **e**, AlphaLisa binding assay measuring the apparent binding affinity (*K*_d_^app^) for wild-type PPAT–NUDT5 and NUDT5 mutant complexes in the presence of AMP (1 mM). Data are mean ± s.e.m. from *n* = 3 independent experiments. ND, not determined*. K*_d_^app^ values were compared by global nonlinear regression with an extra sum-of-squares F-test. ***P < 0.001. Exact *P* values are included as source data. Source data

**Extended Data Fig. 3:**
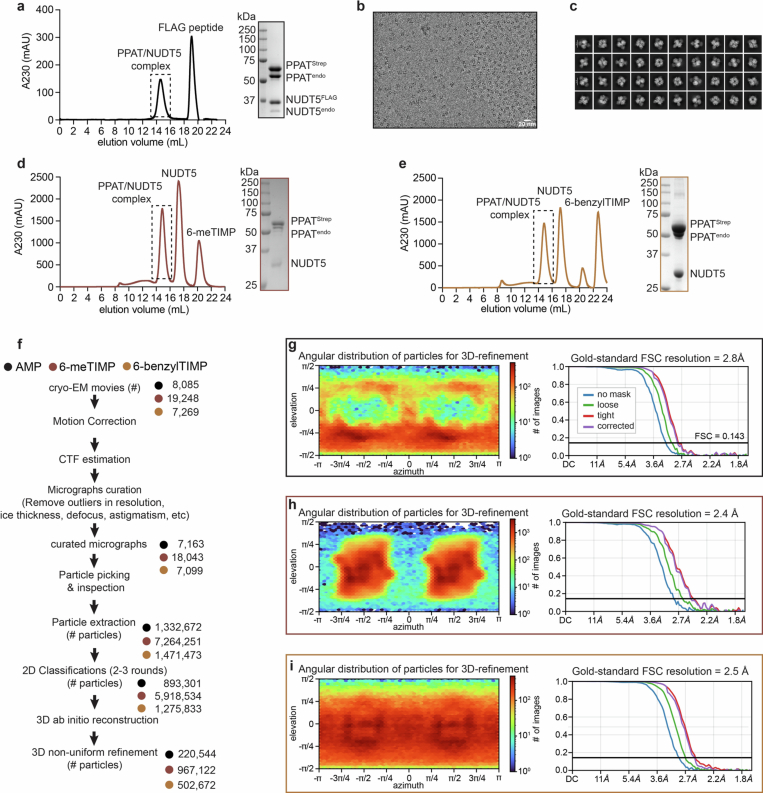
Cryo-EM sample preparation and workflow. **a**. Size exclusion chromatography (SEC) trace (left) and Coomassie-stained gel of PPAT–NUDT5–AMP complex (right) used for cryo-EM analysis. **b**. Motion-corrected and denoised micrograph of PPAT–NUDT5–AMP complex. **c**. Representative 2D class averages for PPAT–NUDT5–AMP complex. Micrographs and 2D class averages from the 6-meTIMP and 6-benzylTIMP datasets were highly similar to that of the AMP-bound dataset. **d**,**e**. SEC and Coomassie-stained gels of PPAT–NUDT5 complexes formed in the presence of **d**. 6-meTIMP and **e**. 6-benzylTIMP. **f**. Cryo-EM data processing particle flow for the three nucleotide-bound PPAT–NUDT5 structures. All processing steps were performed in cryoSPARC. **g**–**i**. Angular distribution of particles and FSC curves from refinements of PPAT–NUDT5–purine complexes with **g**. AMP, **h**. 6-meTIMP and **i**. 6-benzylTIMP.

**Extended Data Fig. 4:**
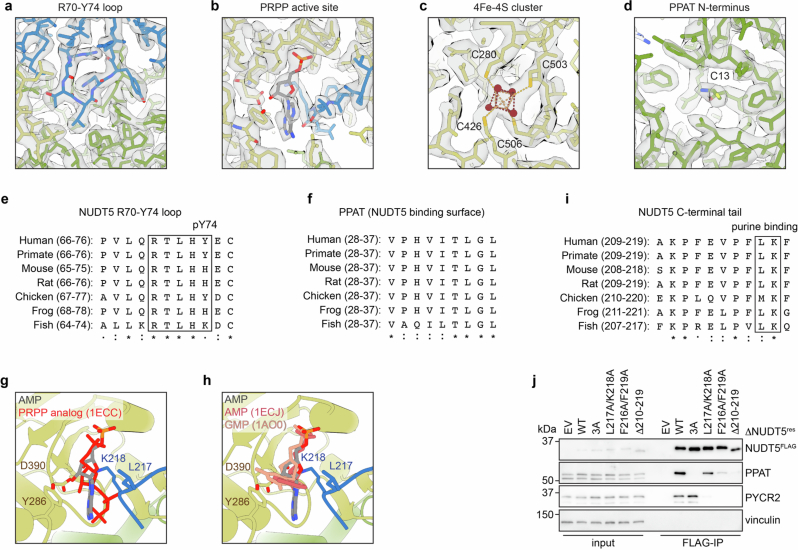
Structural and sequence analysis of the PPAT–NUDT5–AMP complex. **a**–**d**. Example cryo-EM density from the PPAT–NUDT5–AMP map with fit molecular model highlighting: **a**. the R70–Y74 interface **b**. AMP bound at the molecular-glue interface/PRPP active site. **c**. the 4Fe–4S cluster bound by PPAT and **d**. the processed N-terminus of PPAT. **e**,**f**. Multiple sequence alignment (MSA) of **e**. NUDT5 R70–Y74 loop region and **f**. A NUDT5-binding region of PPAT. **g**,**h**. Alignment of PPAT protomers from the human PPAT–NUDT5–AMP complex and **g**. *E*. *coli* PPAT bound to a non-hydrolysable PRPP analogue (PDB: 1ECC), **h**. *E*. *coli* PPAT bound to AMP (PDB: 1ECJ) and *B*. *subtilis* PPAT bound to GMP (PDB: 1AO0). Only the nucleotides are shown from the bacterial structures for clarity **i**. MSA of NUDT5 C-terminal tail region. **j**. Western blot of Flag immunoprecipitations from ∆*NUDT5* HEK293T cells re-expressing wild-type NUDT5–3×Flag and C-terminal tail mutants. Blot is representative of two independent experiments.

To determine how NUDT5 regulates PPAT, we purified a complex between NUDT5(Y74F) and PPAT in the presence of AMP, which had been suggested to be important for PPAT stability^38^ (Extended Data Fig. 3a). The structure of the PPAT–NUDT5(Y74F) complex was determined by cryo-electron microscopy (cryo-EM), yielding a density map with a global resolution of 2.8 Å that was sufficient for high-confidence model building (Fig. 2a,b, Extended Data Figs. 3b,c,f,g and 4a,b, Extended Data Table 1 and Supplementary Video 1). The structure revealed that NUDT5 dimers occupy each elliptical face of a PPAT tetramer, forming an approximately 330-kDa complex. We observed density for four 4Fe–4S clusters bound by PPAT, but did not detect its N-terminal propeptide, which suggests that PPAT was present in its mature form (Extended Data Fig. 4c,d). Each NUDT5 monomer makes contacts with two PPAT protomers from opposing dimers at distinct interfaces, enforcing a tetrameric form of PPAT that has been reported to be more sensitive to feedback inhibition^12,14^.

In agreement with AlphaFold modelling, the R70–Y74 loop of NUDT5 interacts with a conserved surface of PPAT that includes a hydrophobic pocket lined by residues H31, V32, L35 and I242, and mutation of this loop abolished complex formation (Fig. 2c,e and Extended Data Figs. 2 and 4e,f). NUDT5 Y74 projects into a region of PPAT that includes D28; this implies that both repulsion and steric hindrance occur in a phosphorylated state, explaining the absence of this modification in PPAT–NUDT5 complexes (Fig. 1h). Notably, our structural analyses identified a second interaction interface, in which the C terminus of NUDT5 extends into the catalytic centre of a PPAT molecule in the opposing dimeric unit relative to where the R70–Y74 loop binds (Fig. 2d and Extended Data Fig. 4g). The NUDT5 C terminus is anchored by an AMP molecule that occupies the PPAT active site. Reminiscent of a molecular glue stapling NUDT5 to PPAT, AMP sandwiches between PPAT and NUDT5 and contacts residues of each protein (PPAT: Y94, Y286 and D390; NUDT5: L217 and K218) through its purine ring and through hydrogen bonding with a ribose hydroxyl group. The position of AMP in the PPAT active site is distinct from structures of bacterial PPAT orthologues which, in the absence of NUDT5, are inhibited by AMP and guanosine-5′-monophosphate (GMP) through negative feedback^39^ (Extended Data Fig. 4h). Mutation of conserved C-terminal NUDT5 residues that interact with AMP decreased its association with PPAT (Fig. 2e and Extended Data Fig. 4i,j), which indicates that AMP acts as a molecular glue that tethers a rate-limiting metabolic enzyme, PPAT, to its inhibitor, NUDT5.

## PPAT inhibition through metabolite glues

**Fig. 3:**
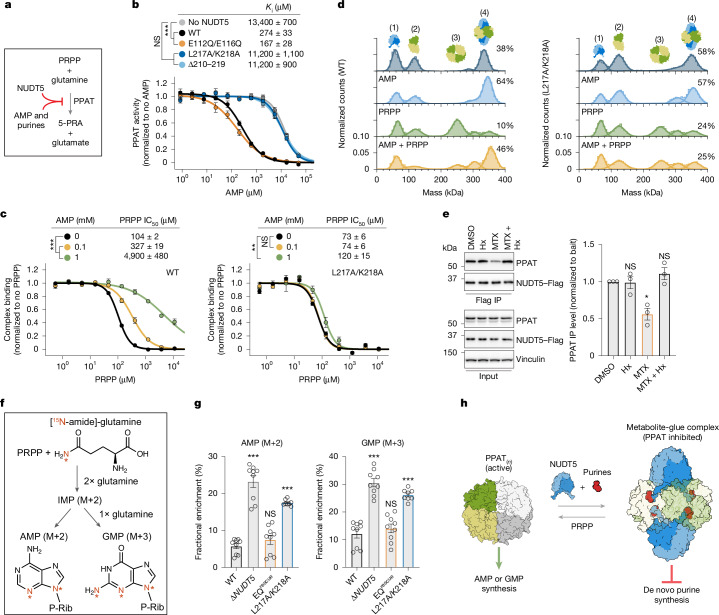
Regulation of PPAT through metabolite glues. **a**, Activity of PPAT, outlining the effects of purines and NUDT5. **b**, PPAT activity assay measuring AMP-dependent inhibition in the absence and presence of wild-type NUDT5 and mutants (1 mM PRPP). *K*_i_, inhibition constant. Data are mean ± s.e.m. from *n* = 3 independent experiments. **c**, PRPP-induced dissociation of PPAT–NUDT5 complexes (AlphaLisa) containing wild-type NUDT5 (left) or NUDT5(L217A/K218A) (right) in the absence and presence of AMP. IC_50_, half-maximum inhibitory concentration. Data are mean ± s.e.m. from *n* = 3 independent experiments. **d**, Mass photometry of PPAT–NUDT5 complexes with the indicated metabolite treatments. Peaks represent: (1) NUDT5_2_ and PPAT_1_; (2) PPAT_2_; (3) PPAT_4_; and (4) PPAT_4_–NUDT5_4_. A minor peak between (3) and (4) is PPAT_4_–NUDT5_2_. Cartoons denote complexes; percentages indicate relative amount of fully assembled PPAT_4_–NUDT5_4_. Data are representative of two independent experiments. **e**, Left, western blot of endogenous NUDT5–3×Flag immunoprecipitation after 16 h of treatment with MTX (2 µM), hypoxanthine (Hx; 50 µM) or both. Right, quantification of PPAT co-immunoprecipitation normalized to NUDT5–3×Flag bait. Data are mean ± s.e.m. with individual values from *n* = 3 independent biological replicates. **f**, [^15^N-amide]-glutamine labelling of purines in the de novo biosynthesis pathway. The orange colour and asterisk denotes the ^15^N-labelled position. P-Rib, ribose monophosphate. **g**, AMP (M+2) and GMP (M+3) fractional enrichment in [^15^N-amide]-glutamine labelling of cells treated with hypoxanthine. Data are mean ± s.e.m. with individual values from *n* = 8 (L217A/K218A only) or *n* = 9 biological replicates from 3 independent experiments. **h**, Model of PPAT–NUDT5 metabolite-glue-based regulation of de novo purine synthesis. *K*_i_ and IC_50_ values in **b**,**c** were compared by global nonlinear regression with an extra sum-of-squares F-test. Significance was determined by two-tailed one-sample *t*-test versus DMSO in **e** and by one-way ANOVA with Dunnett’s multiple-comparisons test versus wild type in **g**. NS, not significant. *P < 0.05, **P < 0.01 and ***P < 0.001. Exact *P* values are included as source data. Source data

**Extended Data Fig. 5:**
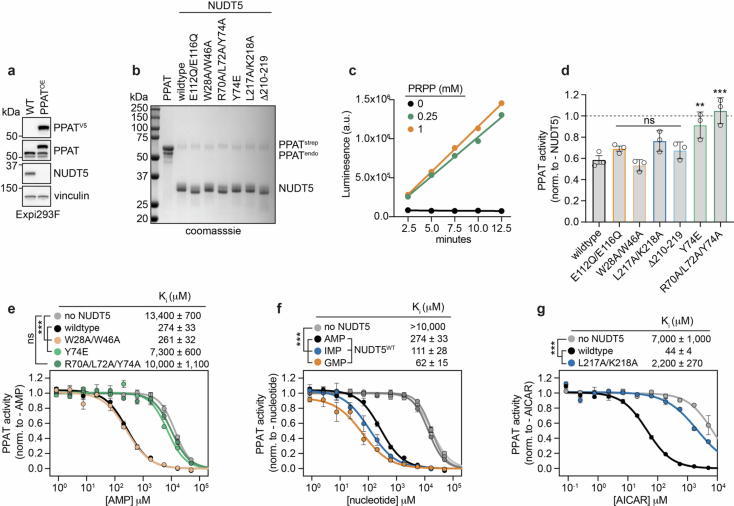
Reconstitution of PPAT activity. **a**. Western blot of engineered Expi293F cell line expressing PPAT–V5-TwinStrep. **b**. Coomassie-stained gel of purified PPAT and NUDT5 proteins. **c**. Time-course PPAT activity assay showing linearity of assays conducted with 0.25 and 1 mM PRPP. Data represent the mean of n = 2 technical replicates from one enzyme preparation. Similar linearity was observed with two separate PPAT preparations used for activity assays. **d**. PPAT activity with NUDT5 in the absence of AMP (1 mM PRPP). NUDT5 mutants ‘WWAA’ is W28/W46A, which abrogates NUDT5 Nudix hydrolase activity by interfering with ADP-ribose binding, and ‘LKAA’ is L217A/K218A. Data represent the mean ± s.e.m. with individual values from n = 3 independent experiments. **e**–**g**. PPAT activity assay measuring inhibitory effects of **e**. AMP in the absence and presence of wild-type NUDT5 and indicated mutants (1 mM PRPP), **f**. AMP, GMP and IMP in the absence and presence of wild-type NUDT5 (1 mM PRPP) and **g**. AICA-ribonucleotide in the absence and presence of wild-type NUDT5 and indicated mutants (0.25 mM PRPP). Data in **e**–**g** represent the mean ± s.e.m. from n = 3 independent experiments. Statistical significance in **d** was assessed using one-way ANOVA followed by Dunnett’s multiple-comparisons test against wild type. *K*_i_ values in **e**–**g** were compared by global nonlinear regression using an extra sum-of-squares F-test. Significance is indicated as ns (not significant), *P < 0.05, **P < 0.01 and ***P < 0.001. Exact *P* values are included as source data. Source data

Although plant hormones and therapeutic compounds are known molecular glues^1–3^, whether endogenous metabolites provide similar regulation in human cells remains to be established. To determine whether AMP is a molecular glue that inhibits purine synthesis by tethering NUDT5 to PPAT, we purified PPAT from Δ*NUDT5* cells and reconstituted its enzymatic activity (Fig. 3a and Extended Data Fig. 5a–c). We then asked whether AMP affected the ability of NUDT5 to modulate PPAT activity. AMP or NUDT5 alone inhibited PPAT, but only when present at high non-physiological concentrations, consistent with a nucleotide-independent binding site provided by the R70–Y74 loop (Fig. 3b and Extended Data Fig. 5d). Notably, the presence of NUDT5 stimulated the ability of AMP to shut off PPAT by around 50-fold, thereby allowing for PPAT regulation at endogenous levels of AMP and NUDT5 (Fig. 3b).

To investigate whether PPAT inhibition is mediated through a glue mechanism, we mutated NUDT5 L217 and K218, the main residues in NUDT5 that contact AMP. Indeed, these residues were required fοr NUDT5- and AMP-dependent PPAT inhibition (Fig. 3b). Mutation of the R70–Y74 loop that provides the second interface with PPAT also impaired NUDT5 function, in line with the notion that molecular glues stabilize pre-existing weak interactions^3^ (Extended Data Fig. 5e). PPAT regulation was not affected by mutations in NUDT5 that inactivate nucleotide hydrolysis (Fig. 3b and Extended Data Fig. 5e). Other purines that were predicted to fit into the glue pocket, such as inosine-5′-monophosphate (IMP), GMP or the biosynthesis intermediate AICA-ribonucleotide, also inhibited PPAT in a manner stimulated by NUDT5 and dependent on an intact glue pocket (Extended Data Fig. 5f,g). Thus, several purine nucleotides seem to act as molecular-glue stabilizers of the inhibited PPAT–NUDT5 complex. Because these molecular glues are derived from endogenous metabolites, we refer to them as metabolite glues.

**Extended Data Fig. 6:**
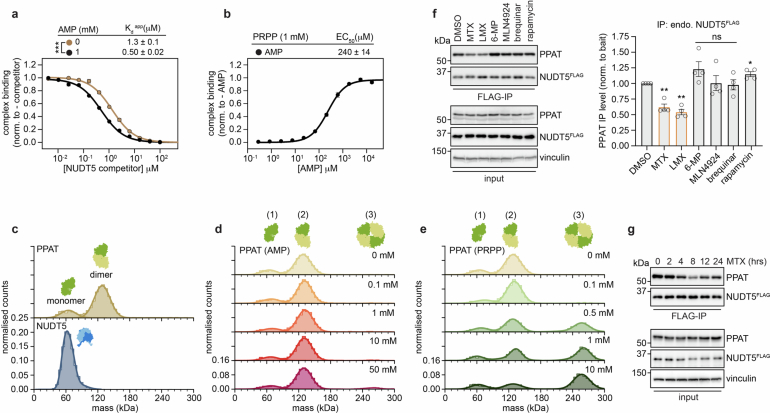
PPAT–NUDT5 complex assembly. **a**. AlphaLisa binding assay measuring apparent binding affinity (*K*_d_^app^) for PPAT–NUDT5 binding in the absence and presence of AMP (1 mM). Data represent the mean ± s.e.m. from n = 3 independent experiments. The AMP (1 mM) condition is also shown in Fig. 2e. **b**. AlphaLisa binding assay quantifying the stabilization of PPAT–NUDT5 binding by AMP in the presence of PRPP (1 mM). Data represent the mean ± s.e.m. from n = 3 independent experiments. **c**–**e**. Mass photometry analysis of purified PPAT and NUDT5 and PPAT incubated with the indicated metabolites: buffer only (**c**), AMP (**d**), or PRPP (**e**). Data in **c**–**e** are representative of two independent experiments. **f**. Left – Representative Western blot of immunoprecipitations from endogenous *NUDT5*^*3×Flag*^ HEK293T cells treated with indicated drugs for 16 h: methotrexate (MTX; 2 µM), lometrexol (LMX; 10 µM), 6-mercaptopurine (6-MP; 50 µM), MLN4924 (1 µM), brequinar (2 µM), and rapamycin (1 µM). Right, quantification of PPAT co-immunoprecipitation relative to NUDT5–3×Flag bait and normalized to a DMSO-treated control condition. Data represent the mean ± s.e.m. with individual values from n = 3 biological replicates. **g**. Time-course immunoprecipitation of endogenous NUDT5–3×Flag from HEK293T cells treated with MTX (2 µM). Blot shown is representative of two independent experiments. Values for *K*_d_^app^ in **a** were compared by global nonlinear regression using an extra sum-of-squares F-test. Statistical significance in **f** was determined using two-tailed one-sample *t*-tests relative to the DMSO control. Significance is indicated as ns (not significant), *P < 0.05, **P < 0.01 and ***P < 0.001. Exact *P* values are included as source data. Source data

Whereas disrupting the R70–Y74 interface prevented PPAT–NUDT5 assembly, mutating glue-contacting residues (NUDT5(L217A/K218A)) or omitting AMP had more modest effects on initial complex formation (Fig. 2e and Extended Data Fig. 6a). Despite this, our experiments showed that both NUDT5 mutants impaired AMP-dependent PPAT inhibition similarly (Fig. 3b and Extended Data Fig. 5e), suggesting that the AMP glue has functions beyond driving initial interactions. Providing a potential explanation, we found that the PPAT substrate phosphoribosyl diphosphate (PRPP), which binds to the same site as AMP, triggers disassembly of the PPAT–NUDT5 complex (Fig. 3c,d and Extended Data Fig. 6b). Mass photometry revealed that PRPP dismantles the octameric PPAT–NUDT5 complex into PPAT tetramers and NUDT5 dimers (Fig. 3d and Extended Data Fig. 6c–e). Of note, the presence of AMP shielded wild-type complexes from disassembly, but it did not exert this function in the presence of the NUDT5(L217A/K218A) mutant (Fig. 3c,d). These findings reveal that the metabolite glues also counteract substrate-induced dissociation of PPAT–NUDT5 complexes, thereby limiting de novo purine synthesis to conditions of high PRPP or low AMP levels.

To simulate these metabolic conditions in cells, we used MTX to increase PRPP and decrease AMP by blocking 10-formyl-tetrahydrofolate availability^18,40^. In agreement with our binding data, acute MTX treatment caused the dissociation of endogenous PPAT–NUDT5 complexes (Fig. 3e and Extended Data Fig. 6f,g). Supplementing MTX-treated cells with hypoxanthine, which is converted into IMP, AMP or GMP metabolite glues, completely blocked complex dissociation. In addition to MTX, the GART inhibitor lometrexol caused PPAT–NUDT5 dissociation, but compounds targeting pyrimidine synthesis or mTOR signalling did not affect complex integrity (Extended Data Fig. 6f). Together, these findings underscore the notion that metabolite glues prevent premature disassembly of the inhibitory PPAT–NUDT5 complex to control PPAT activation.

**Extended Data Fig. 7:**
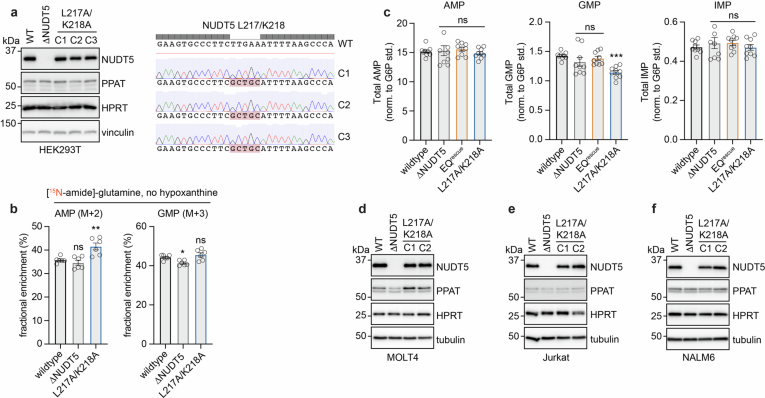
Characterization of endogenous NUDT5 molecular-glue mutants. **a**. Left, western blot of HEK293T cell lines used in this study. Right, aligned Sanger sequencing traces of endogenous L217A/K218A clones compared with the wild-type sequence. **b**. Fractional enrichment of AMP (M+2) and GMP (M+3) isotopologues in [^15^N-amide]-glutamine labelling experiments conducted without added hypoxanthine. Data represent the mean ± s.e.m. with individual values from n = 6 biological replicates from two independent experiments. **c**. Measurements of purine monophosphate levels normalized to a glucose-6’-phosphate internal isotopic standard in HEK293T cells treated with hypoxanthine identically to those used for [^15^N-amide]-glutamine labelling experiments in Fig. 3g. Data represent the mean ± s.e.m. with individual values from n = 9 biological replicates from 3 independent experiments. **d**–**f**. Western blots of ALL cell lines used in this study: MOLT4 (**d**), Jurkat (**e**) and NALM6 (**f**). Statistical significance in **b**,**c** was determined by one-way ANOVA followed by Dunnett’s multiple-comparisons test against wild type. Significance is indicated as ns (not significant), *P < 0.05, **P < 0.01 and ***P < 0.001. Exact *P* values are included as source data. Source data

If stabilizing the PPAT–NUDT5 complex were relevant for signalling, loss of glue recognition should affect the ability of cells to respond to changes in purine abundance. We therefore mutated L217 and K218 in endogenous *NUDT5* to selectively ablate metabolite-glue recognition and then monitored de novo purine synthesis by stable isotope tracing of [^15^N-amide]-glutamine (Fig. 3f and Extended Data Fig. 7a). We conducted these experiments in the presence of hypoxanthine, which feeds the salvage pathway and should suppress de novo synthesis^11^. We observed limited ^15^N-labelling of purines in wild-type cells and in cells that expressed hydrolase-inactive NUDT5(EQ), but noted a robust increase in ^15^N-incorporation when *NUDT5* had been deleted (Fig. 3g and Extended Data Fig. 7b); this confirms that NUDT5 inhibits de novo synthesis in the presence of sufficient purines. Notably, cells expressing NUDT5(L217A/K218A) incorporated ^15^N into purines to a similar extent to that seen in Δ*NUDT5* cells (Fig. 3g). Total purine levels remained consistent across labelling experiments (Extended Data Fig. 7c), which showed that PPAT dysregulation, rather than changes in the purine pool, drove enhanced de novo synthesis in the absence of glue recognition. Together, these findings show that purines act as metabolite glues that stabilize the PPAT–NUDT5 complex and thereby block flux through de novo synthesis if sufficient purines are available (Fig. 3h).

## Thiopurines are molecular glues

**Fig. 4:**
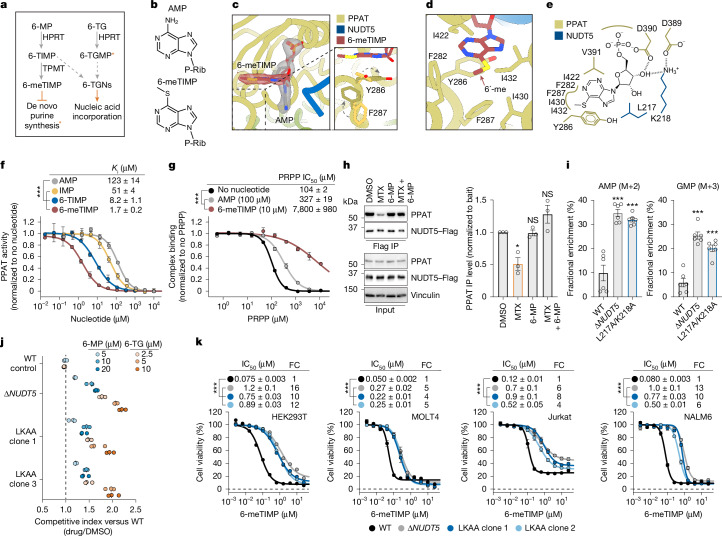
Thiopurines are molecular glues that inhibit PPAT. **a**, Metabolism of 6-MP and 6-TG. Orange asterisk indicates 6-TGMP conversion to 6-meTGMP, which is a PPAT–NUDT5 glue. **b**, AMP and 6-meTIMP structures. **c**, Left, alignment of the AMP and 6-meTIMP glue interface with cryo-EM density. Right, PPAT residue rearrangement in 6-meTIMP-bound (dark) versus AMP-bound (light) structures. **d**, PPAT hydrophobic pocket engaged by 6-meTIMP. **e**, 6-meTIMP glue interface. **f**, PPAT activity assay with wild-type NUDT5 and nucleotide (0.25 mM PRPP). Data are mean ± s.e.m. from *n* = 3 independent experiments (*n* = 2 for 6-TIMP). **g**, PRPP-induced PPAT–NUDT5 dissociation (AlphaLisa) with AMP and 6-meTIMP. Data are mean ± s.e.m. from *n* = 3 independent experiments. Data are also shown in Fig. 3c. **h**, NUDT5–3×Flag immunoprecipitation (left) and quantification (right) after treatment with MTX (2 µM) and/or 6-MP (50 µM). Data are mean ± s.e.m. with individual values from *n* = 3 independent biological replicate experiments. **i**, AMP (M+2) and GMP (M+3) fractional enrichment in [^15^N-amide]-glutamine labelling of cells treated with 6-MP. Data are mean ± s.e.m. with individual values from *n* = 6 biological replicates from 2 independent experiments. **j**, FACS growth competition: *∆NUDT5* and *NUDT5*^*L217A/L218A*^ (LKAA) mutants versus wild-type HEK293T cells treated with 6-MP or 6-TG. Data are mean with individual values from *n* = 3 biological replicates. **k**, Viability of wild-type, *∆NUDT5* and *NUDT5*^*L217A/L218A*^ ALL cells treated with 6-meTIMP. Data are mean ± s.e.m. of *n* = 6 biological replicates from 3 independent experiments. FC (fold change) is the ratio of mutant to wild-type IC_50_. *K*_i_ and IC_50_ values in **f**,**g**,**k** were compared by global nonlinear regression using an extra sum-of-squares F-test. Significance was determined by two-tailed one-sample *t*-test relative to DMSO control in **h** and by one-way ANOVA followed by Dunnett’s multiple-comparisons test against wild type in **i**. NS, not significant. *P < 0.05 and ***P < 0.001. Exact *P* values are included as source data. Source data

**Extended Data Fig. 8:**
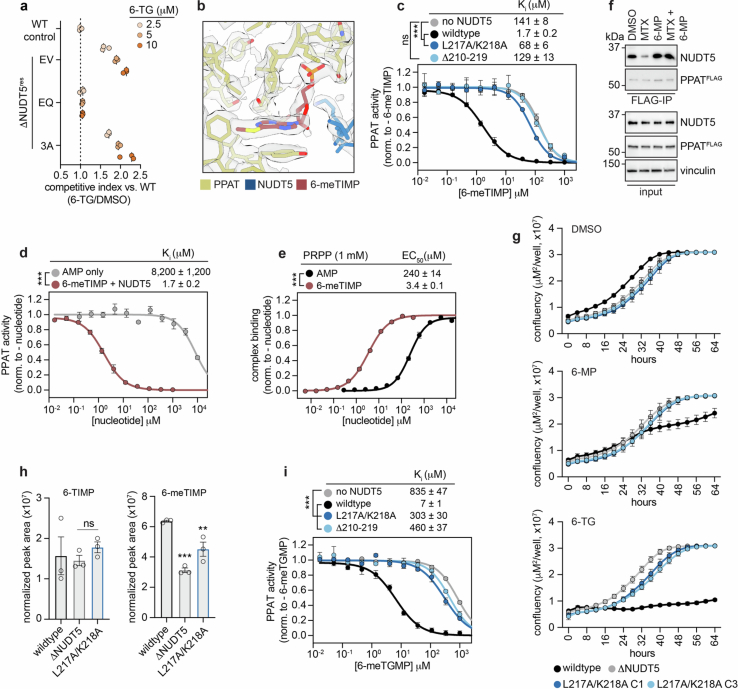
Chemotherapeutics operate through PPAT–NUDT5. **a**. FACS-based growth competition comparing Δ*NUDT5* and rescue mutants with wild-type HEK293T cells treated with 6-TG. Data represent the mean with individual values from n = 3 biological replicates. **b**. Cryo-EM density of the PPAT–NUDT5 6-meTIMP molecular-glue interface with model fit. **c**,**d**. PPAT activity assay measuring inhibitory effects of 6-meTIMP in the absence and presence of wild-type NUDT5 and indicated mutants (**c**) and compared to AMP only (**d**) with 0.25 mM PRPP. Data represent the mean ± s.e.m. from n = 3 independent experiments. **e**. AlphaLisa binding assay quantifying the stabilization of PPAT–NUDT5 binding by AMP and 6-meTIMP in the presence of PRPP (1 mM). Data represent the mean ± s.e.m. from n = 3 independent experiments. Data for the AMP condition are also shown in Extended Data Fig. 6b. **f**. Western blot of endogenous PPAT–3×Flag immunoprecipitations after 16-h treatment with MTX (2 µM), 6-MP (50 µM) and MTX + 6-MP. Blot is representative of two independent experiments **g**. Time-resolved microscopy (incucyte) growth assays of wild-type and mutant HEK293T cells treated with the indicated drugs. Data represent the mean ± s.e.m. of n = 6 biological replicates. **h**. Levels of intracellular 6-TIMP and 6-meTIMP metabolites after 16-h treatment with 6-MP (20 µM). Data represent the mean ± s.e.m. with individual values from n = 3 biological replicates. **i**. PPAT activity assay measuring inhibitory effects of 6-meTGMP in the absence and presence of wild-type NUDT5 and mutants. Data represent the mean ± s.e.m. from n = 3 independent experiments. *K*_i_ values in **c**,**d**,**i** as well as EC_50_ in **e** were compared by global nonlinear regression using an extra sum-of-squares F-test. Statistical significance in **h** was determined by one-way ANOVA followed by Dunnett’s multiple-comparisons test against wild type. Significance is indicated as ns (not significant), *P < 0.05, **P < 0.01 and ***P < 0.001. Exact *P* values are included as source data. Source data

The binding of molecular glues at the interface of two proteins complicates efforts to improve the potency of therapeutic compounds, and previous work has shown that chemical modification of molecular glues can in fact rewire the composition of stabilized protein complexes^41,42^. Because the PPAT–NUDT5 interface accommodates multiple purines, its metabolite-glue pocket might show increased flexibility and hence support the recognition of small molecules with therapeutic effects. To test this notion, we turned to 6-MP and 6-thioguanine (6-TG), two chemotherapeutics that are transformed in cells into metabolites that inhibit PPAT at the same site as purines^6,43^ (Fig. 4a). Although these molecules were long thought to act by being incorporated in the genome to cause replicative stress, reports^33–37^ have indicated that the cytotoxic effects of thiopurine metabolites are also mediated through regulating PPAT. Indeed, we found that Δ*NUDT5* cells were highly resistant to 6-TG (Extended Data Fig. 8a). Expression of NUDT5(3A), which does not bind to PPAT and is resistant to metabolite-glue regulation, did not resensitize Δ*NUDT5* cells to 6-TG, suggesting that thiopurine chemotherapeutics might act through a glue mechanism.

To test this hypothesis, we solved a 2.4-Å-resolution structure of PPAT–NUDT5 bound to the 6-MP metabolite 6-methylthioinosine-5′-monophosphate (6-meTIMP) (Extended Data Fig. 3d,f,h). The 6-meTIMP engaged the same subunit interface between PPAT and NUDT5 as AMP did (Fig. 4b,c and Extended Data Fig. 8b). However, the purine ring of 6-meTIMP showed a distinct binding orientation in the PPAT active site, compared with AMP. Whereas 6-meTIMP and AMP are nearly aligned by their ribose phosphates, the purine ring of 6-meTIMP adopted a bent conformation, compared with the extended AMP. This is mediated by rearrangement of the PPAT Y286 and F287 residues to form a distinct π-stacking interaction between the 6-meTIMP purine ring and PPAT Y286 that is not seen in the AMP-bound structure. The reorientation of the 6-meTIMP purine ring allows the 6-methylthio substituent to engage a hydrophobic pocket composed of PPAT residues F282, F287, I422, I430 and I432, which provides additional stabilizing contacts (Fig. 4d,e). These findings therefore not only suggest that thiopurine metabolites act as molecular glues in the PPAT–NUDT5 complex, but also reveal extensive flexibility of the glue pocket, which gives 6-meTIMP additional contacts to its protein partners.

Consistent with this structure, 6-meTIMP is an exceptionally strong inhibitor of PPAT that is around 70-fold more efficient than AMP and relies on an intact glue interface with NUDT5 (Fig. 4f and Extended Data Fig. 8c). Reflecting its enhanced potency, PPAT inhibition by 6-meTIMP and NUDT5 was around 5,000-fold more effective than by AMP alone (Extended Data Fig. 8d), and the 6-meTIMP glue strongly shielded PPAT–NUDT5 complexes from PRPP-induced disassembly (Fig. 4g and Extended Data Fig. 8e). Addition of the thiopurine precursor of 6-meTIMP, 6-MP, to cells also prevented dissociation of the PPAT–NUDT5 complex in MTX-treated cells, showing that thiopurine glues can overcome endogenous regulation of purine biosynthesis (Fig. 4h and Extended Data Fig. 8f). However, *NUDT5*^*L217A/K218A*^ mutant cells did not shut down purine synthesis in the presence of 6-MP and were highly resistant to thiopurines in growth assays (Fig. 4i,j and Extended Data Fig. 8g). Wild-type and mutant cells treated with 6-MP contained similar quantities of 6-TIMP and 6-meTIMP metabolites, indicating that these phenotypes are not due to a defect in purine salvage (Extended Data Fig. 8h).

To delineate the potential contribution of blocking purine synthesis to thiopurine-mediated therapies, we introduced the L217A/K218A glue-defective mutation into endogenous *NUDT5* loci of cell lines representing acute lymphoblastic leukaemia (ALL) subtypes currently treated with 6-MP (MOLT4, Jurkat and NALM6). We then treated these cells with increasing concentrations of 6-meTIMP. Δ*NUDT5* and *NUDT5*^*L217A/K218A*^ cells showed a reduced response to concentrations of 6-meTIMP that are used to treat patients, revealing an important contribution of the glue mechanism^44^. Cytotoxicity was observed independently of NUDT5 at higher drug concentrations. We observed similar PPAT inhibition using the methylated 6-TG metabolite 6-methylthio-guanosine-5′-monophosphate (6-meTGMP), suggesting that this compound also inhibits de novo purine synthesis as a molecular glue (Extended Data Fig. 8i). We conclude that thiopurines are molecular glues that exert their effects, at least in part, by blocking de novo purine synthesis through PPAT–NUDT5. Structural flexibility in the glue pocket allows thiopurines to act more potently than endogenous nucleotides, thereby bypassing regulatory mechanisms such as PPAT activation through increased PRPP that would normally disassemble the PPAT–NUDT5 complex.

## Design of enhanced molecular glues

**Fig. 5:**
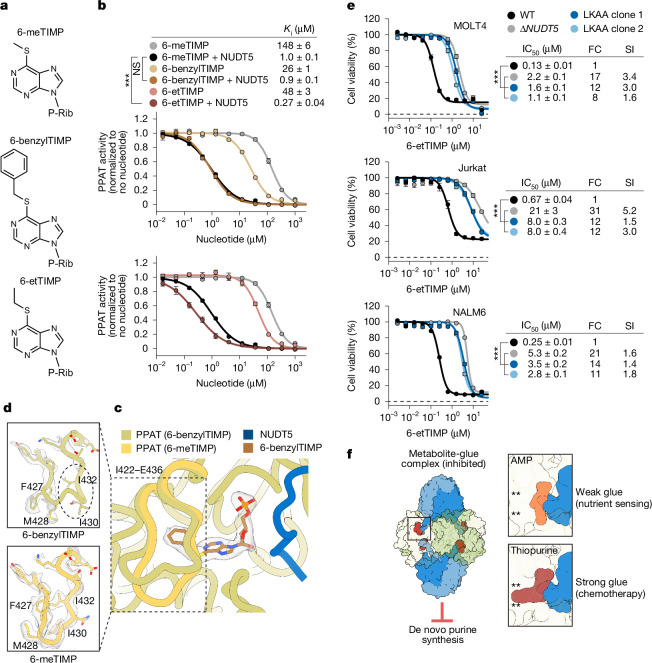
Design of improved molecular glues. **a**, Chemical structures of 6-meTIMP, 6-benzylthioinosine-5′-monophosphate (6-benzylTIMP) and 6-ethylthioinosine-5′-monophosphate (6-etTIMP). **b**, PPAT activity assay measuring nucleotide-dependent inhibition in the absence and presence of NUDT5. Data are mean ± s.e.m. from *n* = 3 independent experiments. The same data for 6-meTIMP are shown in both panels for clarity. **c**, Structural alignment of the region surrounding the molecular-glue interface for the 6-benzylTIMP- and 6-meTIMP-bound PPAT–NUDT5 structures. Cryo-EM density for the 6-benzylTIMP is shown as a transparent surface. **d**, Sharpened cryo-EM density of the I422–E436 loop in the 6-benzylTIMP and 6-meTIMP maps shown at the same contour. The dashed oval indicates a region of missing density in the 6-benzylTIMP map that is well-defined in the 6-meTIMP map. **e**, Viability of wild-type, *∆NUDT5* and endogenous *NUDT5*^*L217/K218A*^ mutant cells treated with 6-etTIMP for 72 h. Data are mean ± s.e.m. of *n* = 6 biological replicates from 3 independent experiments. FC (fold change) is the ratio of mutant to wild-type IC_50_, representing fold change in 6-etTIMP resistance. Selectivity index (SI) is the ratio of 6-etTIMP to 6-meTIMP fold change relative to wild type, quantifying relative on-target cytotoxicity. An SI greater than 1 is indicative of increased on-target cytotoxicity. **f**, Model of the NUDT5- and purine- or thiopurine-dependent metabolite-glue mechanism. The double asterisks represent the PPAT hydrophobic pocket. *K*_i_ and IC_50_ values in **b**,**e** were compared by global nonlinear regression using an extra sum-of-squares F-test. NS, not significant. ***P < 0.001. Exact *P* values are included as source data. Source data

As conformational flexibility of a glue pocket could facilitate therapeutics development, we examined the consequences of attaching bulkier substituents to 6-TIMP. We hypothesized that a 6-benzyl-thio group should only be accommodated if the PPAT–NUDT5 interface can undergo larger conformational changes than seen for 6-meTIMP. Notably, 6-benzylTIMP retained similar PPAT inhibition to 6-meTIMP in the presence of NUDT5 and was around fivefold more potent in its absence (Fig. 5a,b), indicating that the metabolite-glue interface discovered here is even more pliable than anticipated.

**Extended Data Fig. 9:**
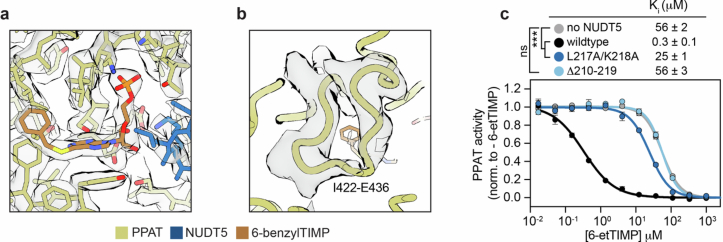
Characterization of thiopurine derivatives. **a**. Cryo-EM density of the PPAT–NUDT5–6-benzylTIMP glue interface with fit model. **b**. I422–E436 loop of PPAT in the 6-benzylTIMP complex modelled into the unsharpened cryo-EM density map and shown at a low contour. **c**. PPAT activity assay measuring inhibitory effects of 6-etTIMP in the absence and presence of wild-type NUDT5 and indicated mutants. Data represent the mean ± s.e.m. from n = 3 independent experiments. *K*_i_ values were compared by global nonlinear regression using an extra sum-of-squares F-test. Significance is indicated as ns (not significant), *P < 0.05, **P < 0.01, and ***P < 0.001. Exact *P* values are included as source data. Source data

To understand the molecular basis for flexible glue recognition, we solved the structure of 6-benzylTIMP bound to PPAT–NUDT5 (Fig. 5c and Extended Data Figs. 3e,f,i and 9a). Although 6-benzylTIMP binds in the same overall orientation as 6-meTIMP, the 6-benzyl group further reorients PPAT residues in the hydrophobic pocket that were observed to form stabilizing interactions with the 6-methylthio substituent of 6-meTIMP. This disrupts the local PPAT structure around a loop comprising residues I422–E436, causing the loop to move away from the benzyl substituent and increasing flexibility in the region, as indicated by a loss of high-resolution cryo-EM density (Fig. 5c,d and Extended Data Fig. 9b). Thus, the metabolite-glue pocket in the PPAT–NUDT5 complex can undergo marked changes to accommodate larger ligands, in a manner that does not degrade glue potency.

Our structural insight opened the possibility of designing more-potent thiopurine glues. We noted that the 6-methylthio substituent of 6-meTIMP occupies a hydrophobic pocket of PPAT that is not engaged by AMP. 6-meTIMP is also a more potent inhibitor than its unmethylated precursor 6-TIMP, suggesting that added hydrophobicity at this position enhances inhibitor strength (Fig. 4f). In line with this notion, inclusion of an ethyl group to form 6-ethylthioinosine-5′-monophosphate (6-etTIMP) resulted in threefold greater inhibition of PPAT, compared with 6-meTIMP, in a manner dependent on the glue interface (Fig. 5b and Extended Data Fig. 9c). To investigate the therapeutic properties of 6-etTIMP, we measured its effects on the viability of wild-type, Δ*NUDT5* and glue-deficient *NUDT5*^*L217A/K218A*^ ALL cells (Fig. 5e). Like 6-meTIMP (Fig. 4k), 6-etTIMP showed dose-dependent cytotoxicity that was diminished in Δ*NUDT5* and *NUDT5*^*L217A/K218A*^ cells. Notably, however, 6-etTIMP showed a larger difference between wild-type and Δ*NUDT5* cells than did 6-meTIMP, as reflected by its more favourable selectivity index (Fig. 5e). Thus, the flexibility of the PPAT–NUDT5 metabolite-glue pocket can be exploited to improve the properties of chemotherapeutics that have been in clinical use without alteration for decades.

## Discussion

Here, we have uncovered a mechanism of regulation that is centred on a class of molecules referred to as metabolite glues (Figs. 3h and 5f). The identified metabolite glues restrict de novo purine synthesis under nutrient-replete conditions, showing that the glue mechanism provides a means of purine sensing that safeguards cells from the misallocation of metabolic resources. Our work therefore establishes that endogenous molecular glues can serve crucial regulatory functions in human cells. Extending this finding, we show that thiopurine chemotherapeutics, clinically used for around 70 years, act as enhanced glues to inhibit de novo purine synthesis and suppress cell growth. Structural comparison of drug-bound complexes revealed a marked conformational flexibility of the metabolite-glue pocket, indicating that investigating metabolic control mechanisms could provide a fruitful avenue for the discovery of therapeutically relevant molecular glues.

Foundational work suggested that molecular glues enhance weak, pre-existing interactions to bring affinities between binding partners into a biologically meaningful range^2,4,5,45,46^. Consistent with this principle, each NUDT5 protomer engages PPAT at two distinct regions, only one of which is stabilized by the molecular glue. At the glue interface, the NUDT5 C-terminal tail is anchored at the PPAT active site through interactions with the nucleotide and surrounding regions of PPAT. This interaction is crucial for PPAT feedback inhibition, allowing cells to detect purines at micromolar levels that reflect endogenous purine monophosphate concentrations^47^ of 10–40 μM. Biochemical approaches revealed that purine metabolite glues both promote formation and prevent substrate-induced disassembly of the inhibitory PPAT–NUDT5 complex. In this manner, purine metabolite glues oppose premature PPAT activation and thereby prevent unnecessary flux through an energetically costly metabolic pathway.

Although metabolite glues serve as a primary mode of regulation, they act by reinforcing a second site centred on the R70–Y74 loop of NUDT5. This second interface contributes to PPAT inhibition in a glue-independent manner, potentially rigidifying a less active PPAT tetramer without blocking its active site. The R70–Y74 interface is broken by phosphorylation of Y74, which could couple complex formation to signalling inputs from growth factors or the cell cycle. Combined regulation by metabolite glues and phosphorylation might allow cells to upregulate purine production quickly during replication, DNA repair or transcriptional bursting, which require increased levels of purine nucleotides^48^. We expect that metabolite glues frequently cooperate with other forms of regulation to rapidly adjust biosynthetic pathways to the complex needs of a eukaryotic cell. Metabolite glues could therefore provide a mechanism to embed biosynthetic control within larger signalling networks.

Our discovery of glue-dependent regulation of purine synthesis provides practical insight to facilitate glue discovery. Unlike therapeutic glues that bind to rigid and narrowly defined pockets, the conformational flexibility of the PPAT–NUDT5 metabolite-glue pocket permits substantial ligand modifications without sacrificing inhibitor potency or specificity. Supporting this, substitution of the 6-methylthio group of 6-meTIMP with an ethyl group increased glue potency in vitro and enhanced NUDT5-dependent cytotoxicity, potentially by limiting off-target effects. We predict that structural flexibility is a recurring feature of metabolite-glue pockets, reflecting the evolutionary adaptation of biosynthetic enzymes to diverse substrates and metabolic regulators. As proteomic platforms are beginning to enable the identification of weak protein–metabolite interactions and the subsequent discovery of glue-regulated complexes^49^, leveraging the flexibility of metabolite-glue pockets could facilitate the rational development of therapeutic glues that are inspired by endogenous regulation. We therefore anticipate that the study of metabolite glues will not only provide more examples of nutrient sensing, but also expand the therapeutic potential of molecular glues as an emerging drug class.

## Methods

### Data reporting

No statistical methods were used to predetermine sample size. The experiments were not randomized, and the investigators were not blinded to allocation during experiments and outcome assessment.

### Mammalian cell culture

U2OS and HEK293T cells were maintained in Dulbecco’s modified Eagle’s medium (DMEM) with Glutamax (Gibco, 10566016), and MOLT4, Jurkat and NALM6 cells were maintained in RPMI-1640 medium with glutamine (Gibco, 11875093). The medium was supplemented with 10% fetal bovine serum (FBS; VWR, 89510-186) and 1× penicillin–streptomycin (Pen-Strep; Gibco, 15140122) at 37 °C and 5% CO_2_. The growth medium for *PPAT-*knockout cells was supplemented with 100 µM adenine. For drug treatment immunoprecipitation experiments, cells were plated in DMEM with Glutamax, 10% dialysed FBS (dFBS; Cytiva, SH30079.02) and 1× Pen-Strep. For metabolomics experiments, cells were plated in DMEM (high glucose, no glutamine; Gibco, 11960044) supplemented with 2 mM l-glutamine (Gibco, A2916801), 10% dFBS and 1x Pen-Strep. Further details about cell culture conditions for isotopic tracing experiments can be found under ‘Mass-spectrometry-based metabolomics’. Expi293F cells were maintained in Epxi293 medium (Gibco; A1435101) in shaker flasks at 130 rpm and 8% CO_2_. Cell stocks were obtained from and authenticated by (using short tandem repeat profiling) the University of California Cell Culture Facility (RRID: SCR_017924) and were routinely tested for mycoplasma contamination.

### CRISPR editing and endogenous cell line generation

To generate CRISPR knockout *NUDT5* and *PPAT* lines in HEK293T, Expi293F, MOLT4, Jurkat and NALM6 cells, we used a mixture of three optimized sgRNAs (Gene Knockout Kit v.2, Synthego). A total of 1.5 μl sgRNA mixture (100 μM) was incubated with 3.2 μl Cas9-NLS protein (40 μM; produced by the University of California, Berkeley (UC Berkeley) MacroLab) for 10 min at room temperature. Ribonucleoproteins (RNPs) were nucleofected into 5 × 10^5^ cells resuspended in Lonza SF solution (Lonza, V4XC-2032) using a Lonza 4D Nucleofector X-unit and pulse code CM-130 (HEK293T and Expi293F), CA-137 (MOLT4) or CM-138 (NALM6). Jurkat cells were nucleofected in Lonza SE solution (Lonza, V4XC-1032) using pulse code CL-120. Cells were recovered for 3-4 days, split once and then cloned by single-cell dilution in 96-well plates. Expi293F cells were not cloned, because NUDT5 bulk depletion was observed to be more than 95% by western blot. Clones were assessed for knockout by western blotting and phenotypic assay. Uncropped western blots and gels are included as Supplementary Fig. 1. To establish *NUDT5-*knockout lines used for experiments, five clones were combined at equal cell number into one pool to minimize clonal bias effects. A similar editing strategy was used for endogenous 3×Flag epitope tagging and *NUDT5*^*L217A*^^/^^*K218A*^ mutant generation, except that a single sgRNA was used and 1 μl Alt-R HDR single-stranded oligodeoxynucleotide (ssODN) donor template (100 μM stock; IDT) was included in the nucleofection mixture added after RNP formation. For knock-in generation, cells were recovered in HDR Enhancer v.2 (IDT) for 16 h after nucleofection, followed by a change of medium. Knock-in clones were validated by Sanger sequencing. The sgRNA and ssODN sequences used are listed below:

sgRNA sequences: *NUDT5* gene knockout (gKO) 1: 5′-CTTGCAGGTCTCATAGATGA-3′; *NUDT5* gKO 2: 5′-ATCAATCCCTTCCCAGACGG-3′; *NUDT5* gKO 3: 5′-ATACCAACCTGGAGAACATT-3′; *PPAT* gKO 1: 5′-TGATAGCAGTAGGACAATAA-3′; *PPAT* gKO 2: 5′-GTGTCTGATATAAATGACAA-3′; *PPAT* gKO 3: 5′-CATCTCTGTGCATTATAAGC-3′; *NUDT5* endo-3×Flag: 5′-AATGGAGAGCCAAGAACCAA-3′; *PPAT* endo-3×Flag: 5′-TGTAGAATTAGAATGGTAGC-3′; *NUDT5*^*L217A*/*K218A*^: 5′-TGGGCTTAAAATTTCAAGAA-3′.

ssODN sequences: ^*3*×*Flag*^*NUDT5*: 5′-AACTTCTCACCTGAGGGCTGTAAAGACTCGTTTGAAAATGGACTACAAGGACCACGACGGCGATTATAAGGATCACGACATCGACTACAAAGACGACGATGACAAGGGTGGGAGTGGCGGGAGTGAGAGCCAAGAACCAACGGAATCTTCTCAGAATGGCAAACAGTATATCATTTC-3′; *PPAT*^*3*×*Flag*^: 5′-AGCTTGTCTCACTGGAAAATATCCTGTAGAATTAGAATGGGGTGGCAGCGGCGGTTCTGGTGGCAGCGACTACAAGGACCACGACGGCGATTATAAGGATCACGACATCGACTACAAAGACGACGATGACAAGTAGCTGGTAGGGTTGGATGTGTGTAGTTTCAAGATAGAAAG-3′; *NUDT5*^*L217A*/*K218A*^: 5′-TCGTTTACAAAAATGGCCAGTGTCATATTTGGGCTTAAAATGCAGCGAAGGGCACTTCAAATGGCTTTGCATTTGCATGTTTCAGT-3′.

### Stable cell line generation

Lentiviral particles were generated by co-transfecting pLVX-IRES-puromycin or blasticidin constructs encoding the desired protein mutants with second-generation lentiviral packaging plasmids into HEK293T cells using Mirus Trans-IT LT1 transfection reagent (Mirus, MIR 2304). For FACS-based growth competition experiments, pLVX-mCherry-P2A-blasticidin and pLVX-GFP-P2A-blasticidin constructs were used to establish fluorescent cell lines for co-culture. After transfection of the plasmid mixture, the medium was changed the next day, and virus was collected 48 h after the medium change by filtering through a 0.45-µm filter. For stable cell line generation, cells were infected with lentiviral particles at a multiplicity of infection (MOI) < 1 in medium supplemented with polybrene (8 μg ml^−1^). Cells were selected with antibiotic (1 μg ml^−1^ puromycin or 10 μg ml^−1^ blasticidin) around 48 h after transduction until all cells on a control plate were dead, at which point selected cells were recovered without antibiotics for at least 2 days before assaying.

### Metabolism-focused CRISPR screen

For the MLN4924 metabolism-focused CRISPR screen, we used a custom sgRNA library with 8 sgRNAs per gene targeting around 3,000 metabolic enzymes and transporters cloned into the pLentiCRISPR-V2 vector (a gift from K. Birsoy). Lentiviral particles were generated by co-transfecting the sgRNA library with third-generation lentiviral packaging plasmids into HEK293T cells using Mirus Trans-IT LT1 transfection reagent (Mirus, MIR 2304). U2OS cells grown in DMEM with Glutamax, 10% FBS and 1× Pen-Strep were transduced with lentivirus at an MOI of around 0.5 and 500-fold sgRNA coverage in medium supplemented with 8 μg ml^−1^ polybrene for 48 h. Transduced cells were selected with puromycin (1.5 μg ml^−1^) for 4 days, and recovered for an additional 2 days, after which a zero time point was collected for the screen (30 × 10^6^ cells). The selected and expanded cells were split into two pools maintaining 1,000-fold sgRNA coverage throughout the screen and treated with either dimethyl sulfoxide (DMSO) or sublethal doses of MLN4924 (150 nM). Cells were split every 4 days, and fresh DMSO or MLN4924 was added after allowing cells to re-adhere to plates for 4 h. On day 24, 30 × 10^6^ cells were collected per condition and genomic DNA was extracted using a QIAamp DNA Blood Maxi kit (QIAGEN, 51192) according to the manufacturer’s protocol. sgRNA-encoding regions were amplified with ExTaq DNA polymerase (Takara, RR001) with unique barcoded primers. PCR products were combined and 50 μl was gel purified from a 3% agarose TBE gel. Amplicons were sequenced using an Illumina NextSeq2000 by the UC Berkeley QB3 Genomics core facility. Data were analysed using the MAGeCK analysis package^50^, comparing normalized sgRNA representation on day 24 between DMSO- and MLN4924-treated cell pools.

For screen validation, competition experiments were performed as outlined in ‘Cellular growth assays’, with the following sgRNA sequences that were not present in the original metabolism-focused screening library:

NTC: 5′-GGCCGATAATGATCCGACCG-3′; *ABCB1*: 5′-TAGTAGGATTTACACGTGGT-3′; *ABCG2*: 5′-TTCTTGGATGAGCCTACAAC-3′; *ADSL*: 5′-CGAGCCAGTCTACCCACATT-3′; *ADSS*: 5′-TCAAGCAGCTGATGGTATCC-3′; *ATIC*: 5′-AAACCACGCTCGAGTGACAG-3′; *GART*: 5′-CGAGTACTTATAATTGGCAG-3′; *GMPS*: 5′-CGAACAGTTCCCTCACTCTT-3′; *IMPDH1*: 5′-ACACCCGGGACACGAGACGG-3′; *IMPDH2*: 5′-GCAGTGAAGTCGATGTACCC-3′; *NUDT5*: 5′-ACAGTTCCGACCACCAATGG-3′; *PAICS*: 5′-TACGAATTGTTAGACAGTCC-3′; *PFAS*: 5′-GCGGCACACTGACACGATGT-3′; *PPAT*: 5′-GTGATCACTCTGGGACTCGT-3′.

### Cellular growth assays

All growth assays were conducted in DMEM with Glutamax supplemented with 10% FBS and 1x Pen-Strep. Specific drugs are noted under ‘Drugs and chemicals’ and amounts are specified in the figures and legends.

#### FACS-based growth competition

For growth competition experiments, GFP- or mCherry-expressing cell populations for comparison were established using lentiviral expression as described above. For CRISPR screening validation experiments, GFP-expressing U2OS cells were transduced with pLentiCRISPR-V2 lentivirus particles containing sgRNAs targeting the indicated genes (sgRNA sequences listed above) and an mCherry population was transduced with a non-targeting control sgRNA (sgNTC). After puromycin selection and recovery, GFP-knockout and mCherry-sgNTC cells were mixed in 12-well plates in equal cell number, and either DMSO or MLN4924 (100 nM) was added. Cells were split every 4 days, re-adding drug at each split. After 12 days, the ratio of GFP^+^ to mCherry^+^ cells was determined by flow cytometry using a BD LSRFortessa instrument and analysed using FlowJo (v.10.8.1). GFP^+^ to mCherry^+^ cell ratios for each knockdown competition condition in the presence of MLN4924 were normalized to equivalent DMSO-treated control conditions. The FACS gating strategy for growth competition experiments is outlined in Supplementary Fig. 2.

For drug treatment growth competition assays using HEK293T cells, GFP^+^ and mCherry^+^ cell populations were established as described above. For rescue experiments, NUDT5 was reintroduced to Δ*NUDT5* cells by lentiviral expression using a pLVX-^3×Flag^NUDT5-IRES-puromycin construct as described above. In brief, 2.5 × 10^4^ wild-type HEK293T mCherry^+^ cells were co-plated with 2.5 × 10^4^ GFP^+^ cells in 12-well plates. The drug was added the following day, and cells were analysed by flow cytometry 72 h after addition. GFP^+^ to mCherry^+^ cell ratios for drug treatment wells were normalized to equivalent DMSO-treated cell mixtures.

#### CellTiter Glo

A total of 2.5 × 10^3^ U2OS cells were seeded in 96-well clear-bottom plates (Corning, 165306) in 50 μl medium. MLN4924 was added the following day at 2× concentration in 50 μl medium. Viability was measured after 72 h by the addition of 100 μl CellTiter Glo reagent per well (Promega, G7570) according to the manufacturer’s instructions, and a luminescence signal was measured on a Perkin Elmer EnVision microplate reader.

#### Incucyte

A total of 5 × 10^3^ HEK293T cells were seeded in black 96-well clear-bottom plates in 100 μl medium (Corning, 3904). Drugs were added the following day at 2× concentration in 100 μl medium. Cell growth was monitored using time-resolved microscopy in an Incucyte S3 microscope.

#### CellTiter Blue

Cells were seeded in black 96-well clear-bottom plates (Corning, 3904) at 20,000 cells per well (MOLT4 and Jurkat) or 15,000 cells per well (NALM6) in 50 μl RPMI-1640 medium supplemented with 10% FBS. HEK293T cells were seeded at 4,000 cells per well in DMEM with 10% FBS and allowed to attach for 24 h. 6-meTIMP (final concentration (*C*_f_) = 0–25 μM) diluted in 50 μl of medium was added to the cells. A fully dead control treated with staurosporine (1 μM) was used for normalization in all assays. After 72 h of treatment, 20 μl of CellTiter Blue reagent (Promega, G8080) was added to each well. Plates were incubated at 37 °C and 5% CO_2_ for around 2 h, after which fluorescence signal was read using a PerkinElmer Envision microplate reader. Values for the dead control were subtracted from all wells, and raw data were normalized to the top values (*Y*_max_) of the viability curve. Normalized data were plotted in GraphPad Prism (v.10) and fitted using the ‘[Inhibitor] versus response (four parameters)’ equation with top values fixed at 100% viability, and the bottom value was unfixed. Reported half-maximum inhibitory concentration (IC_50_) values are mean ± s.e.m. from *n* = 9 observations from 3 independent biological replicates.

### Drugs and chemicals

The following drugs and chemicals were used in this study at amounts specified in figures and legends: pevonedistat (MLN4924; MedChemExpress, HY-70062), MTX (MedChemExpress, HY-14519), lometrexol (LMX; MedChemExpress, HY-14521), brequinar (MedChemExpress, HY-108325), rapamycin (Adooq Biosciences, A10782), 5-phospho-d-ribose 1-diphosphate (PRPP; Sigma-Aldrich, P8296), l-glutamine (Sigma-Aldrich, G8540); adenosine-5′-monophosphate; (AMP; Sigma-Aldrich 01930), inosine-5′-monophosphate (IMP; MedChemExpress, HY-W010759), guanosine-5′-monophosphate (GMP; Sigma-Aldrich, G8377), AICA-ribonucleotide (Cayman Chemicals, 33907), adenine (Thermo Fisher Scientific, A17622.14), hypoxanthine (MedChemExpress, HY-N0091), 6-TG (Thermo Fisher Scientific, B21280.03), 6-MP (Adooq Biosciences, A15898), 6-thioinosine-5′-monophosphate (6-TIMP; Jena Biosciences, NU-1148), 6-methylthioinosine-5′-monophosphate (6-meTIMP; Jena Biosciences, NU-1226), 6-methylthioguanosine-5′-monophosphate (6-meTGMP; Jena Biosciences, NU-1128), 6-benzylthioinosine-5′-monophosphate (6-benzylTIMP; WuXi, custom synthesis); staurosporine (Selleckchem, AM-2282); 6-ethylthioinosine-5′-monophosphate (6-etTIMP; WuXi, custom synthesis) amd 6-ethylmercaptopurine riboside (6-EMPR; WuXi, custom synthesis). Liquid chromatography–mass spectrometry (LC–MS) and proton nuclear magnetic resonance (HNMR)/carbon nuclear magnetic resonance (CNMR) spectra for chemicals synthesized by WuXi are attached as Supplementary Fig. 3.

### Immunoprecipitation

#### Small-scale immunoprecipitation

Cells were collected from one 10-cm plate per condition by aspirating medium, scraping and washing in cold Dulbecco’s phosphate-buffered saline (DPBS), and pellets were flash-frozen in liquid nitrogen or immediately processed. Cell pellets were lysed in in 500 μl lysis buffer (30 mM HEPES-NaOH pH 7.5, 150 mM NaCl, 5 mM MgCl_2_, 0.1% NP40) supplemented with EDTA-free protease inhibitors (Roche, 11873580001), PhosStop phosphatase inhibitors (Roche, 4906845001) and benzonase (Millipore, 70746). Lysis was allowed to proceed for 30 min with rotation, and lysates were clarified by centrifugation at 21,000*g*. An input sample was taken from the supernatant by mixing 1:1 with 2× urea sample buffer (120 mM Tris pH 6.8, 4% SDS, 4 M urea, 20% glycerol and bromophenol blue), and the remaining supernatant was incubated with Flag-M2 agarose beads (15 μl of 50% slurry per condition; Millipore, A2220) for 2 h at 4 °C with rotation. Beads were then washed three times with immunoprecipitation buffer and eluted at 65 °C with 1× urea sample buffer.

#### Large-scale immunoprecipitation for mass spectrometry proteomics

For immunoprecipitation–mass spectrometry (IP–MS), cells were collected from 10× 15-cm plates per condition, lysed in 5 ml lysis buffer and processed as described above for the NUDT5(3×Flag) immunoprecipitation using 90 µl Flag-M2 resin slurry per sample. For the PPAT–V5-TwinStrep and NUDT5(3×Flag) sequential immunoprecipitation, supernatant was first bound to Strep-Tactin XT 4Flow resin (IBA, 2-5010-010), washed three times in lysis buffer and eluted in lysis buffer containing 50 mM biotin (2-1016-005). Eluate was then incubated with Flag-M2 affinity resin for 2 h, followed by three washes in lysis buffer. A further three DPBS washes were performed after washes in lysis buffer to remove detergent. Control immunoprecipitation from wild-type HEK293T cells was performed in parallel for all IP–MS experiments. Flag-M2 beads were flash-frozen and further processed by the UC San Diego Proteomics Facility as described in ‘Mass-spectrometry-based proteomics’.

### Western blotting and antibodies

Western blot samples were derived from the immunoprecipitation experiments described above, or samples were lysed in NP40 buffer (30 mM HEPES-NaOH, 150 mM NaCl and 0.5% NP40) supplemented with protease inhibitors and benzonase for 30 min on ice, and clarified by centrifugation at 21,000*g*. Western blot samples were normalized to protein concentration using Pierce 660nm Protein Assay reagent (Thermo Fisher Scientific, 22660). Next, 2× urea sample buffer was added to the samples, which were then denatured at 65 °C. SDS–PAGE and immunoblotting were performed using the indicated antibodies. Images were captured using a ProteinSimple FluorChem M device.

The following antibodies were used in this study, with specific dilutions varying depending on the experiment: anti-PPAT (rabbit, ProteinTech, 15401-1-AP), anti-NUDT5 (rabbit, ProteinTech, 27004-1-AP), anti-PYCR2 (rabbit, ProteinTech, 17146-1-AP), anti-HPRT (rabbit, ProteinTech,15059-1-AP); anti-vinculin (rabbit, CST, 4650), anti-Flag (mouse, Clone M2; Sigma, F1804), anti-DYKDDDDK tag (rabbit, CST, 14793), anti-NRF2 (rabbit, CST, D1Z9C), anti-NEDD8 (rabbit, CST, 19E3), anti-α-tubulin (mouse, DM1A, Calbiochem, CP06), anti-V5 (rabbit, CST, D3H8Q) and anti-HA (rabbit, CST, C29F4).

### Protein expression and purification

#### PPAT

PPAT–V5-TwinStrep was lentivirally expressed in Expi293F human cells in which NUDT5 was bulk depleted using sgRNAs a described above. Protein preparations were performed from 2–6 l of culture collected at around 8 × 10^6^ cells per ml. Purification buffers were extensively vacuum degassed and sparged with nitrogen gas before being cooled on ice. All purification steps were done on ice or at 4 °C. Cell pellets were resuspended in lysis buffer (50 mM HEPES-NaOH pH 7.5, 150 mM NaCl and 1 mM DTT) supplemented with EDTA-free protease inhibitor cocktail and benzonase. Cells were lysed by brief sonication and clarified by centrifugation. The supernatant was treated with BioLock reagent (1 ml per 40 ml lysate; IBA, 2-0205-050) for 20 min then incubated with Strep-Tactin XT 4Flow resin for 90 min with gentle agitation. Resin was transferred into a gravity column and washed with 10 column volumes (CVs) of lysis buffer followed by 20 CVs of lysis buffer supplemented with 10 mM MgCl_2_ and 10 mM ATP heated to 37 °C before application to remove chaperone contamination, and an additional 10 CVs of cold lysis buffer. Protein was eluted in lysis buffer containing 50 mM biotin. The eluate was concentrated (Amicon, UFC9050) and buffer-exchanged using a 0.5-ml Zeba desalting column into lysis buffer without biotin (7 kDa molecular weight cut-off (MWCO); Thermo Fisher Scientific, 89882), flash-frozen in small aliquots and stored at −80 °C for future use. For protein preparations used in AlphaLisa binding assays, PPAT was further purified by size-exclusion chromatography (SEC) using a Superose 6 10/300 increase SEC column (Cytiva) equilibrated in SEC buffer (25 mM HEPES-NaOH pH 7.5, 150 mM NaCl and 1 mM DTT) using an ÄKTA pure 25 FPLC system (Cytiva).

#### NUDT5

His-SUMO-TEV-NUDT5 wild type and mutants were expressed in LOBSTR-BL21(DE3)-RIL cells (Vector Laboratories, NC1789768) induced with 0.5 mM isopropyl β-d-1-thiogalactopyranoside (IPTG) for around 16 h at 18 °C. Cell pellets were resuspended in lysis buffer (50 mM Tris-HCl pH 8, 500 mM NaCl and 10 mM imidazole) supplemented with EDTA-free protease inhibitor cocktail and phenylmethylsulfonyl fluoride (PMSF), benzonase and lysozyme. Cells were lysed by sonication and the supernatant was clarified by centrifugation. Supernatant was applied to a 5-ml HisTrap crude FF column (Cytiva, 17525501) with a syringe pump. The column was washed with 50 ml lysis buffer followed by 25 ml wash buffer (50 mM Tris-HCl pH 8, 500 mM NaCl and 30 mM imidazole) and eluted in 20 ml elution buffer (50 mM Tris-HCl pH 8, 200 mM NaCl and 300 mM imidazole). Eluate was incubated with TEV protease (1:50 w/w; produced in house by MacroLab, UC Berkeley) and dialysed overnight against 4 l buffer (50 mM Tris-HCl pH 8.0, 200 mM NaCl and 2 mM BME). The cleaved product was filtered over a HisTrap column and the flow-through was concentrated and applied to a HiLoad Superdex 75 preparative SEC column (Cytiva, 28989333) equilibrated in storage buffer (25 mM HEPES-NaOH pH 7.5, 150 mM NaCl and 1 mM DTT).

#### PPAT–NUDT5 for cryo-EM (AMP complex)

PPAT–V5-TwinStrep and 3×Flag–NUDT5(Y74F) were co-expressed lentivirally in 4 l wild-type Expi293F cell culture grown in Expi293 medium supplemented with 100 μM adenine and collected at around 8 × 10^6^ cells per ml. All purification steps were performed on ice or at 4 °C in purification buffer (50 mM HEPES-NaOH pH 7.5, 150 mM NaCl, 2 mM AMP and 1 mM DTT). The first Strep-tag immunoprecipitation step was performed as described above to purify PPAT, omitting the MgCl_2_ and ATP wash. Eluate from the Strep-Tactin XT resin was rebound to 1 ml of Flag-M2 agarose resin for 2 h in purification buffer without DTT, washed three times and eluted three times in a total of 6 ml purification buffer supplemented with 1 mg ml^−1^ 3×Flag peptide (Sigma-Aldrich, F4799). The Flag-M2 eluate was concentrated in a 100-kDa-MWCO concentrator (Amicon) and applied to a Superose 6 10/300 increase SEC column (Cytiva, 29091596) equilibrated in SEC buffer (25 mM HEPES-NaOH pH 7.5, 150 mM NaCl, 2 mM AMP and 1 mM DTT). The complex was concentrated to around 9 mg ml^−1^ and used immediately for cryo-EM grid preparation.

#### PPAT–NUDT5 for cryo-EM (6-meTIMP and 6-benzylTIMP complexes)

PPAT–V5-TwinStrep and bacterially expressed NUDT5(Y74F) were purified separately as described above. In this case, we used NUDT5(Y74F) because it forms a more stable complex with PPAT than does the wild-type protein. NUDT5 (75 μM) was incubated with PPAT (15 μM) directly after elution of PPAT from Strep-Tactin resin for 1 h in 2 ml Strep-Tactin elution buffer (50 mM HEPES-NaOH pH 7.5, 150 mM NaCl, 1 mM DTT and 50 mM biotin) supplemented with 200 μM 6-meTIMP (Jena Biosciences, NU-1226) or 6-benzylTIMP (WuXi). The protein mixture was concentrated to 350 μl and the concentration of nucleotide adjusted to 1 mM. After another 1-h incubation, the complex was applied to a Superose 6 10/300 increase SEC column (Cytiva) equilibrated in SEC buffer (25 mM HEPES-NaOH pH 7.5, 150 mM NaCl, 50 μM 6-meTIMP or 200 μM 6-benzylTIMP and 1 mM DTT). Fractions containing the PPAT–NUDT5 complex were concentrated to around 15 mg ml^−1^. The complex was diluted to 10 mg ml^−1^ and a final concentration of 2 mM 6-meTIMP/6-benzylTIMP in cryo-EM buffer (25 mM HEPES-NaOH pH 7.5, 150 mM NaCl and 1 mM DTT) and used immediately for grid preparation.

### Cryo-EM sample preparation, data collection and analysis

Cryo-EM samples were mixed with a final concentration of 0.02% (w/v) fluorinated octylmaltoside (Anatrace, O310F) immediately before cryo-freezing to prevent protein denaturation at the air–water interface. Then, 2.6 μl of the sample was applied to a glow-discharged 300-mesh Quantifoil R1.2/1.3 grid and incubated for 15 s before being blotted and plunge-vitrified in liquid ethane cool-protected by liquid nitrogen. Grid freezing was performed using a Mark IV Vitrobot (Thermo Fisher Scientific) system operating at 12 °C and 100% humidity.

Cryo-EM data were collected using a 300 kV Titan Krios G3i microscope (Thermo Fisher Scientific) equipped with a BIO Quantum energy filter (slit width 20 eV). Data were collected using SerialEM software at a nominal magnification of 105,000× with a pixel size of 0.424 Å per pixel. Movies were recorded using a Gatan K3 Direct Electron Detector operating in super-resolution CDS mode. Each movie was composed of 40 subframes with a total dose of 50 e^−^ per A^2^, resulting in a dose rate of 1.25 e^−^ per A^2^. Data processing, including motion correction, CTF estimation, particle picking, two-dimensional (2D) class averaging and three-dimensional (3D) refinement, was performed using the cryoSPARC v.4.3 workflow^51^, with mostly default settings. All movies were 2× binned and patch motion corrected. After particle picking and several iterations of 2D class averaging, the initial 3D volume was calculated using several rounds of ab initio 3D reconstruction. Selected particles were then used for non-uniform 3D refinement. Default *B*-factor sharpening or local filtering was used to generate the final sharpened maps. Reported resolution is based on the corrected masking value for the gold-standard FSC from refinements in cryoSPARC.

### Model building

The AlphaFold3 PPAT–NUDT5 monomer complex prediction (Extended Data Fig. 2a,b) was performed by co-folding one protomer of NUDT5 and PPAT using AlphaFold3. The initial model for the PPAT–NUDT5 complex (Extended Data Fig. 2c,d) was generated using AlphaFold3^32^ by specifying four chains of NUDT5 (UniProt ID: Q9UKK9), four chains of PPAT omitting the first 11 amino acids (UniProt ID: Q06203) and 8 molecules of AMP. Coordinates for 4Fe–4S clusters were added by aligning *Bacillus subtilis* PPAT (PDB ID: 1GPH) to the AlphaFold model. The model was fitted into the high-resolution cryo-EM density map in ChimeraX (v1.9)^52^, and model regions without adequate density in the unsharpened map were deleted. Models were iteratively refined using a combination of ISOLDE (v.1.2)^53^ in ChimeraX (v.1.9), Coot (v.0.9.4.1)^54^ and phenix.real_space_refine and phenix.validation_cryoem in PHENIX (v.1.21.2-5419)^55^. Models from the AMP-bound datasets were used as initial models for the additional datasets. The Grade2 server was used to generate ligand structure and restraints for the 6-meTIMP and 6-benzylTIMP (Global Phasing).

### PPAT activity assay

PPAT activity assays monitoring glutamate formation were performed in 96-well PCR plates at a final volume of 20 μl, and all reaction components were diluted in PPAT reaction buffer (50 mM HEPES-NaOH pH 7.5, 150 mM NaCl, 5 mM MgCl_2_, 0.1 mg ml^−1^ BSA and 1 mM DTT). First, 5 µl of nucleotide dilution was added to each well, followed by 5 µl of buffer or NUDT5 (*C*_f_ = 10 µM), and then 5 μl of PPAT (*C*_f_ = 50 nM). The initial PPAT species was determined to be mostly dimeric by mass photometry, with a minor monomeric population (Extended Data Fig. 6c). Reaction components were incubated at room temperature for 30 min, then initiated by adding 5 µl of 4× substrate mixture containing PRPP (*C*_f_ = 1 mM; Sigma-Aldrich) and glutamine (*C*_f_ = 4 mM; Sigma-Aldrich). Some assays were performed with 0.25 mM PRPP to better measure competitive inhibitor potency, indicated in the figure legends. Reactions proceeded for 8 min at 37 °C in a thermocycler and were quenched at 98 °C for 2 min. Reaction kinetics were observed to be linear for 12.5 min for reactions at both 0.25 mM and 1 mM PRPP. Reactions were diluted 1:100 in 10 mM HEPES-NaOH pH 7.5, and 20 µl of dilution was transferred to a white 384-well microplate (Corning, 3752). Then, 5 μl of Glutamate-Glo reagent (Promega, J7021) was added to each well and incubated for 2 h, and luminescence signal was measured on a Perkin Elmer EnVision microplate reader.

Raw data were fitted to a four-parameter logistic equation, *Y* = *Y*_min_ + (*Y*_max_ − *Y*_min_)/(1 + (*X*/10^log*K*i^)^*n*^) in Python (v3.11.8) using the scipy.optimize.curve_fit function and fits were manually inspected. Inhibition curves that reached saturation were baseline normalized using both *Y*_min_ and *Y*_max_ values, whereas conditions that did not reach saturation were normalized using only *Y*_max_. Normalized data were plotted in GraphPad Prism (v.10) and fitted using the ‘[Inhibitor] versus response (four parameters)’ equation with the bottom value fixed at 0. The reported *K*_i_ is the mean ± s.e.m. from *n* = 3 independent experiments.

### PPAT–NUDT5 binding assays

#### AlphaLisa assay

To measure the apparent *K*_d_ for NUDT5 wild-type and mutants, PPAT–V5-TwinStrep (125 nM) was incubated with N-terminally tagged wild-type NUDT5(6×His–HA) (125 nM) to form a tracer complex in the presence of increasing amounts (0–100 μM) of untagged wild-type or mutant NUDT5 competitor in binding buffer (25 mM HEPES-NaOH pH 7.5, 150 mM NaCl, 5 mM MgCl_2_, 0.01% NP40 and 0.1% BSA) supplemented with 1 mM AMP. For the measurement without supplemented AMP, tagged PPAT and NUDT5 tracer complex components were incubated at 250 nM each. After a 4-h incubation at 25 °C, binding reactions were diluted to a final concentration of 25 nM of each component, and 12.5 μl of the dilution was mixed with 12.5 μl of 2× AlphaLisa bead mixture in binding buffer containing Strep-Tactin donor beads (40 μg ml^−1^; Revvity, 6760002S) and anti-HA acceptor beads (20 μg ml^−1^; Revvity, AL170C) in a light-grey 384-well plate (Revvity, 6057350). Notably, we observed that the PPAT–NUDT5 complex did not further assemble or dissociate when diluted to assay-compatible concentrations. After a 30-min incubation at 25 °C, the AlphaLisa signal was read using a Perkin Elmer EnVision microplate reader. Bead-only and biotin elution controls were used to ensure signal specificity.

To measure metabolite-induced dissociation and stabilization of the complex, PPAT–V5-TwinStrep (3 μM) was incubated with NUDT5(6×His–HA) (3 μM) in binding buffer for 2 h at 25 °C. For NUDT5(L217A/K218A) complexes, the initial incubation was performed with 10 μM of each component. To measure PRPP-dependent dissociation, complexes were diluted to 50 nM in buffer containing AMP or 6-meTIMP and incubated for 15 min. Binding reactions were further diluted 1:1 with PRPP (*C*_f_ = 0–11 mM) in binding buffer for an additional 30 min. For assay set-ups monitoring the stabilization of PPAT–NUDT5 at a fixed concentration of PRPP, binding reactions were first diluted to 75 nM and mixed 1:1 with a concentration series of AMP (*C*_f_ = 0–16.7 mM) or 6-meTIMP (*C*_f_ = 0–0.33 mM) diluted in binding buffer. After 15 min, PRPP (*C*_f_ = 1 mM) was added, and reactions were incubated for an additional 30 min. Reactions were mixed with AlphaLisa beads and incubated, and the signal was read as described above. Raw data were fitted to a four-parameter logistic equation, *Y* = *Y*_min_ + (*Y*_max_ − *Y*_min_)/(1 + ($X/{10}^{{\mathrm{logIC}}_{\mathrm{50}}}{)}^{n}$) in Python (v.3.11.8) using the scipy.optimize.curve_fit function and fits were manually inspected. Inhibition curves that reached saturation were baseline normalized using both *Y*_min_ and *Y*_max_ values, whereas conditions that did not reach saturation were normalized using only *Y*_max_. Normalized data were plotted in GraphPad Prism (v.10) and fitted using the ‘[Inhibitor] versus response variable slope (four parameters)’ equation with the bottom value fixed at 0. The reported IC_50_ or half-maximum effective concentration (EC_50_) value is the mean ± s.e.m. from *n* = 3 independent experiments.

#### Mass photometry

Complexes were formed for 2 h at 25 °C in binding buffer (25 mM HEPES-NaOH pH 7.5, 150 mM NaCl, 5 mM MgCl_2_ and 1 mM DTT) with PPAT (1 μM) and wild-type NUDT5 (1 μM) or 3 μM of each component for complexes containing NUDT5(L217A/K218A) in the absence or presence of AMP (1 mM). For conditions with PRPP (1 mM), it was added around 1 h before sample measurement. Conditions containing only PPAT or NUDT5 were assembled at 1 μM of protein component. Binding reactions were rapidly diluted in binding buffer and immediately measured using a Refeyn Mass Photometer TwoMP within 10 s of dilution with a normal viewing window and 1-min recording time using AquireMP software (Refeyn). Data were processed using the MassFerence P1 standards for mass estimation (Refeyn, MP-CON-41033) and figures were assembled in the DiscoverMP software (Refeyn). All mass photometry measurements were repeated at least twice in independent experiments.

### Mass-spectrometry-based metabolomics

#### Isotope tracing experiments

Cells were seeded in 6-cm dishes in DMEM with 10% dFBS and 2 mM l-glutamine. For experiments with hypoxanthine, 20 μM was included at plating. For experiments with 6-MP, 20 μM of drug was added around 36 h after plating and around 12 h before the addition of isotopes for tracing. Around 48 h after plating, the medium was exchanged by washing plates once in a minimal medium (DMEM with dFBS) before adding medium containing either [^15^N-amide]-l-glutamine (2 mM; Cambridge Isotope Laboratories, NLM-557) or unlabelled l-glutamine (2 mM) with fresh hypoxanthine or 6-MP (20 µM) supplemented for the described treatments. After labelling for 3 h, cellular metabolism was quenched by aspirating medium, snap-freezing plates in liquid nitrogen and storing at −80 °C until extraction. Plates were collected in parallel accounting for cell number for proactive normalization during sample processing. Intracellular metabolites were extracted by adding extraction solvent (40% acetonitrile, 40% methanol, 20% water and 0.1 M formic acid with 1 μM ^13^C_6_-glucose-6-phosphate and 10 μM ^13^C_1_-fructose-1,6-bisphosphate internal isotopic standards) to each dish (7.5 × 10^6^ cells per ml extraction solvent) and incubated at 4 °C for 10 min. Cell extracts were scraped, neutralized with ammonium bicarbonate (0.1 M final) and stored at −80 °C until analysis. Before injection, cell extracts were centrifuged at 17,000*g* for 10 min at 4 °C, and supernatant was used for analysis.

Metabolites from cellular extracts were injected (10 μl) and separated using the SeQuant ZIC-pHILIC column (5 mm polymeric sorbent, 150 × 2.1 mm; Millipore-Sigma, 1.50460.0001) using an Agilent 1260 Infinity HPLC. Autosampler used a 10-s needle wash between samples (1:1:1 isopropanol, acetonitrile, water) and was maintained at 10 °C. Chromatographic separation was performed using a 30-min linear gradient starting at 10% ammonium acetate (20 mM, pH 9.3) with medronic acid (5 μM; Agilent, 5191-4506) and 90% acetonitrile, and terminating at 30% acetonitrile. Flow rate and column temperature were maintained at 200 μl per min and 15 °C, respectively. A triple-quadrupole mass spectrometer (Agilent 6430 QQQ) equipped with electrospray ionization was coupled to the HPLC system to perform targeted metabolomics through dynamic multiple reaction monitoring (dMRM). The dMRM method used both positive and negative ionization mode, with metabolite *m*/*z* and retention times optimized and validated using an in-house metabolite library. QQQ source parameters were held constant at: gas, 350 °C at 11 l per min; nebulizer, 25 psi; capillary, 3,000 V (both negative and positive). Agilent MassHunter (v.10.1) was used for data acquisition, and Skyline (MacCoss Lab v.24.1.0.214)^56^ was used for analysis. Parameters for metabolite identification are included as Supplementary Table 1.

Each independent experiment contained three biological replicates per sample per treatment group. To quantify the relative abundance of total metabolite pools in unlabelled samples, the total peak area for all measured isotopologues was normalized to the isotopic standard with the closest retention time. For de novo purine synthesis rates in [^15^N-amide]-l-glutamine-labelled samples, all raw peak areas were corrected for the natural abundance of ^15^N, ^13^C, ^18^O and ^2^H isotopes using IsoCorrectoR (v.3.22)^57^. Isotope enrichment was calculated using the corrected peak areas of the fully labelled isotopologue relative to the sum of all biologically relevant isotopologues. For AMP and IMP, relevant isotopologues included M+0 and M+2, where M+2 was deemed fully labelled. For GMP, relevant isotopologues included M+0, M+2 and M+3, where M+3 was deemed fully labelled.

#### Measurements of thiopurine metabolite abundance

Intracellular levels of 6-TIMP and 6-meTIMP were determined by LC–MS/MS. Cells were washed with cold PBS before the addition of a 40/40/20 mixture of LC–MS-grade acetonitrile/methanol/water + 0.1% formic acid for intracellular metabolite extraction. Cells were incubated for 30 min at −20 °C, then centrifuged at 14,000 rpm for 10 min to pellet insoluble material. Isotope-labelled ^13^C-^15^N amino acids (Cambridge Isotope Laboratories, MSK-A2-1.2) were also spiked into each sample as internal standards to monitor analytical conditions. The supernatant was transferred to LC–MS vials for metabolomics analysis, with 2 μl of sample used for analysis.

Untargeted metabolomics was done on a Vanquish UHPLC system coupled with an Orbitrap Exploris 240 mass spectrometer (Thermo Fisher Scientific). Polar metabolites were separated on a XBridge Amide Column (2.1 mm inner diameter (ID) × 100 mm, particle size 3.5 μm; Waters,186004860) which was consistently housed at 40 °C. Mobile phases were prepared as follows: (A) 95/5 (v/v) water/acetonitrile with 10 mM ammonium hydroxide and 10 mM ammonium acetate; (B) 5/95 (v/v) water/acetonitrile with 10 mM ammonium hydroxide and 10 mM ammonium acetate. The mobile phases were delivered at a flow rate of 0.15 ml per min for a 25-min run with the following stepwise gradient for solvent B: 0 min, 85% B; 2.5 min, 70% B; 7 min, 55% B; 16 min, 35% B; 16.1–8 min, 25% B; 18–25 min, 85% B. The electrospray ionization source (ESI) was operated in positive mode. The ion spray voltage was set at 4 kV, with the ion transfer tube temperature set at 350 °C, vaporizer temperature 325 °C, sheath gas 35 arbitrary units (a.u.), aux gas 5 a.u. and sweep gas 1 a.u. Full MS scans of 1 ms were performed at a resolution of 90,000 units, with a scan range of 60–900 *m*/*z*. A pooled quality control (pQC) sample followed by a blank (mobile phase solution) injection was performed among every ten injections of biological samples to monitor instrument performance. The pQC samples were also used for the top ten MS/MS analyses with dynamic exclusion during the analysis for compound identification. Peaks were integrated using the Quan Browser module within Xcalibur and were normalized to protein content for each sample.

### Mass-spectrometry-based proteomics

#### Sample processing

Flag-M2 beads from immunoprecipitations were diluted in TNE (50 mM Tris pH 8.0, 100 mM NaCl and 1 mM EDTA) buffer. RapiGest SF reagent (Waters, 186008090) was added to the mix to a final concentration of 0.1%, and the samples were boiled for 5 min. TCEP was added to 1 mM (final concentration) and the samples were incubated at 37 °C for 30 min. Subsequently, the samples were carboxymethylated with 0.5 mg ml^−1^ iodoacetamide for 30 min at 37 °C followed by neutralization with 2 mM TCEP (final concentration). The protein samples were then digested with trypsin (trypsin:protein ratio, 1:50) overnight at 37 °C. RapiGest was degraded and removed by treating the samples with 250 mM HCl at 37 °C for 1 h followed by centrifugation at 14,000 rpm for 30 min at 4 °C. The soluble fraction was then added to a new tube and the peptides were extracted and desalted using C18 desalting columns (Thermo Fisher Scientific, PI-87782). Peptides were quantified using the BCA assay and a total of 1 μg of peptides were injected for LC–MS analysis.

#### LC–MS analysis

Trypsin-digested peptides were analysed by ultra-high-pressure liquid chromatography (UPLC) coupled with MS/MS using nanospray ionization. The nanospray ionization experiments were performed using a TimsTOF 2 pro hybrid mass spectrometer (Bruker) interfaced with nanoscale reversed-phase UPLC (Evosep One). The Evosep method of 30 samples per day was performed using a 10 cm × 150-μm reversed-phase column packed with 1.5-μm C18-beads (PepSep, Bruker) at 58 °C. The analytical columns were connected with a fused silica emitter (10 μm ID; Bruker Daltonics) inside a nanoelectrospray ion source (captive spray source; Bruker). The mobile phases comprised 0.1% formic acid as solution A and 0.1% formic acid/99.9% acetonitrile as solution B. Data-independent acquisition with parallel accumulation-serial fragmentation (dia-PASEF) MS settings were used for data collection on a TimsTOF Pro 2, with the outlined parameters as follows: the values for mobility-dependent collision energy were set to 10 eV; no merging of TIMS scans was performed; the ion mobility (IM) was set between 0.85 (1/*k*_0_) and 1.3 (1/*k*_0_) with a ramp time of 100 ms; each method includes one IM window per dia-PASEF scan with a variable isolation window at 20 AMU segments; 34 PASEF MS/MS scans were triggered per cycle (1.38 s) with a maximum of 7 precursors per mobilogram; and precursor ions in an *m*/*z* range between 100 and 1,700 with charge states ≥3+ and ≤8+ were selected for fragmentation. Protein identification, label-free quantification, post-translational modification (PTM) quantification and statistical analysis were performed using default settings in Spectronaut 18.0 (Biognosys). The peptide search allowed for two missed cleavages and included phosphorylation (STY) as a variable modification in addition to acetylation and oxidation. Data were mapped to the UniProt reference proteome UP000005640.

For the PPAT–V5-TwinStrep–NUDT5–3×Flag sequential immunoprecipitation and NUDT5–3×Flag immunoprecipitation comparison, DIA protein quantity for candidate hits from both experiments was normalized to that measured for the NUDT5 bait. The resulting gene list was compared to the CRAPome database of common mass spectrometry contaminants, and hits that were found in more than 50% of database experiments were removed from the analysis.

### Multiple sequence alignment

Multiple sequence alignment (MSA) was performed using Clustal Omega^58^. The UniProt sequence identifiers are as follows (species, NUDT5 accession, PPAT accession): human (*Homo sapiens*, Q9UKK9, Q06203); primate (*Macaca mulatta*, A0A1D5QNV6, F7GPV9); mouse (*Mus musculus*, Q9JKX6, Q8CIH9); rat (*Rattus norvegicus*, Q6AY63, P35433); chicken (*Gallus gallus*, A0A8V0XJK7, P28173); frog (*Xenopus laevis*, Q6IND3, A0A974DTF4); fish (*Danio rerio*, Q6IQ66, A6H8S4).

### Software and programs

All software used is freely or commercially available: FACSDiva (v.9.0), FlowJo (v.10.10.0), GraphPad Prism (v.10), SerialEM (v.4.1), AlphaFold 3, Coot (v.0.9.8.92), ChimeraX (v.1.8), PyMOL (v.2.5.5), PHENIX (v.1.21.1-5286), cryoSPARC (v.4.3), Isolde (v.1.2), Spectronaut (v.18.0), Agilent MassHunter (v.10.1), Skyline (MacCoss Lab v.24.1.0.214), Xcalibur (v.4.3). Python (v3.10), SciPy library (v.1.11), Matplotlib (v.3.7), IsoCorrectoR (v.3.22), Refeyn AquireMP (2024 R2) and Refeyn DiscoverMP (2024 R2).

### Reporting summary

Further information on research design is available in the Nature Portfolio Reporting Summary linked to this article.

## Online content

Any methods, additional references, Nature Portfolio reporting summaries, source data, extended data, supplementary information, acknowledgements, peer review information; details of author contributions and competing interests; and statements of data and code availability are available at 10.1038/s41586-026-10790-3.

## Supplementary information

Supplementary Information Supplementary Fig. 1: Uncropped western blots and gels; Supplementary Fig. 2: FACS gating strategy for cell competition assay; Supplementary Fig. 3: Supporting data for custom-synthesized compounds, including LC–MS and NMR analyses of 6-ethylTIMP (SW-01) and 6-benzylTIMP (SW-05); and Supplementary Table 1: Compound identification for targeted metabolomics.

Reporting Summary

Peer Review file

Supplementary Video 1 Cryo-EM structure of the PPAT–NUDT5–AMP complex

## Source data

Source Data Fig. 1

Source Data Fig. 2

Source Data Fig. 3

Source Data Fig. 4

Source Data Fig. 5

Source Data Extended Data Fig. 1

Source Data Extended Data Fig. 5

Source Data Extended Data Fig. 6

Source Data Extended Data Fig. 7

Source Data Extended Data Fig. 8

Source Data Extended Data Fig. 9

## Figures and Tables

**Extended Data Table 1:**
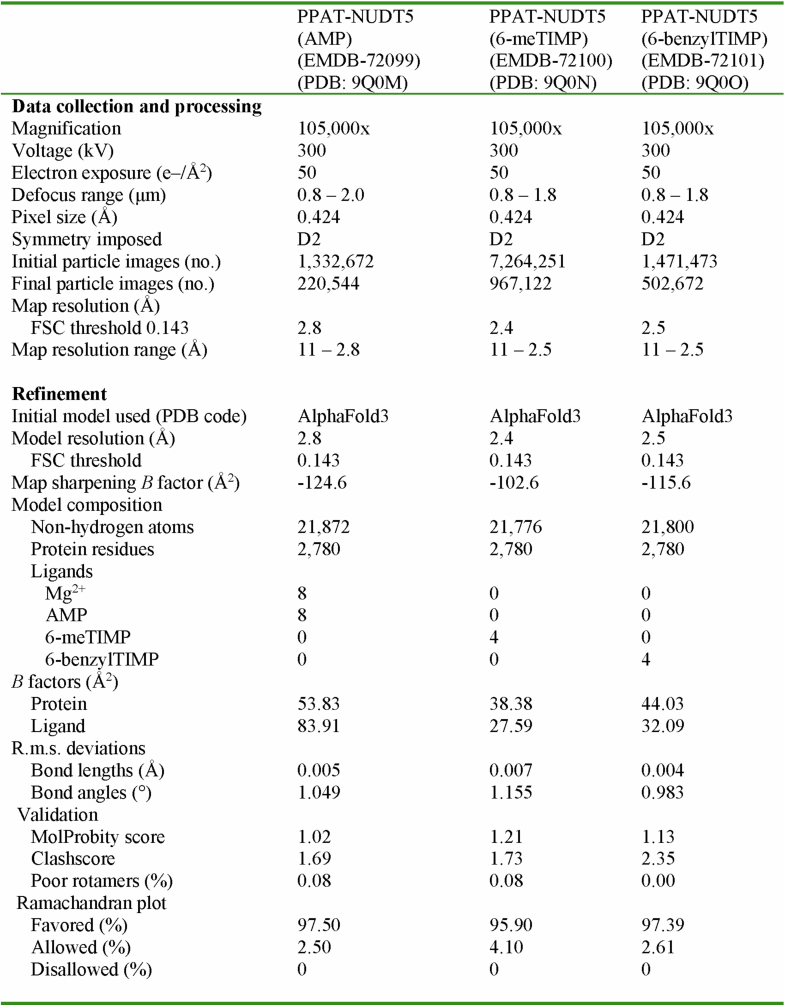
Cryo-EM data collection, refinement and validation statistics

## Data Availability

Structural models of PPAT–NUDT5–nucleotide complexes have been deposited in the Worldwide Protein Data Bank (wwPBD) and cryo-EM maps in the Electron Microscopy Data Bank (EMDB) with the following accession codes: AMP complex (PDB: 9Q0M; EMDB: EMD-72099), 6-meTIMP complex (PDB: 9Q0N; EMDB: EMD-72100) and 6-benzylTIMP complex (PDB: 9Q0O; EMDB: EMD-72101). Numerical and immunoblot source data are provided as Supplementary Fig. 1. The mass spectrometry proteomics data have been deposited to the ProteomeXchange Consortium via the PRIDE partner repository with the dataset identifier PXD077951. Untargeted metabolomics data were deposited in the MassIVE repository with dataset identifier MSV000101706. There are no restrictions on data availability. Source data are provided with this paper.
