# Supplementary material for: Metabolite glues as a means of purine sensing and chemotherapeutic response
Source: Nature. 2026 Jul 15;655(8125):1300–8. doi: 10.1038/s41586-026-10790-3 (PMC13421311; doi:10.1038/s41586-026-10790-3)
Supplement: Supplementary file 1 — Supplementary Fig. 1: Uncropped western blots and gels; Supplementary Fig. 2: FACS gating strategy for cell competition assay; Supplementary Fig. 3: Supporting data for custom-synthesized compounds, including LC–MS and NMR analyses of 6-ethylTIMP (SW-01) and 6-benzylTIMP (SW-05); and Supplementary Table 1: Compound identification for targeted metabolomics. [file 41586_2026_10790_MOESM1_ESM.pdf]

nature portfolio

https://doi.org/10.1038/s41586-026-10790-3

Supplementary information

Metabolite glues as a means of purine sensing and chemotherapeutic response

In the format provided by the authors and unedited

Supplementary Figure 1. Uncropped Western blots and gels.

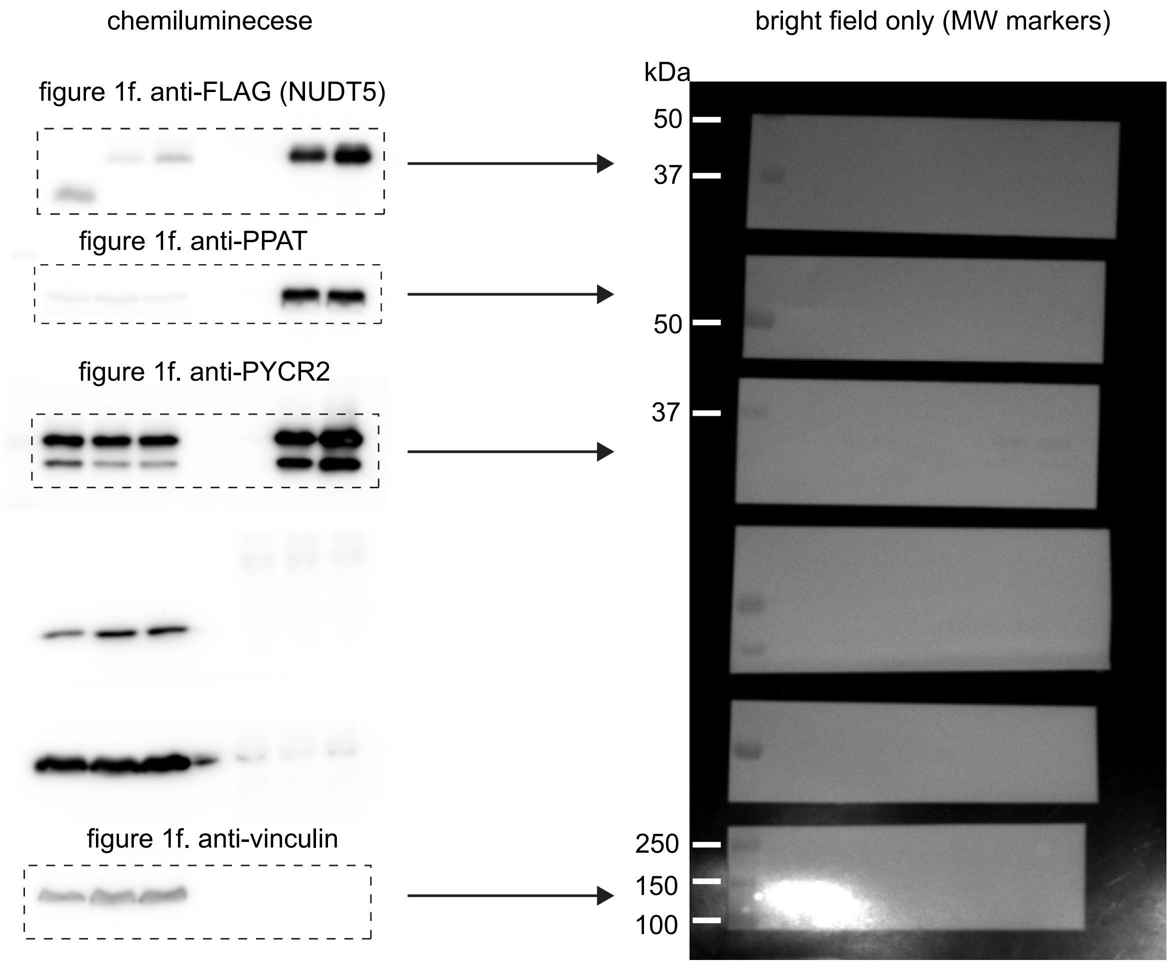

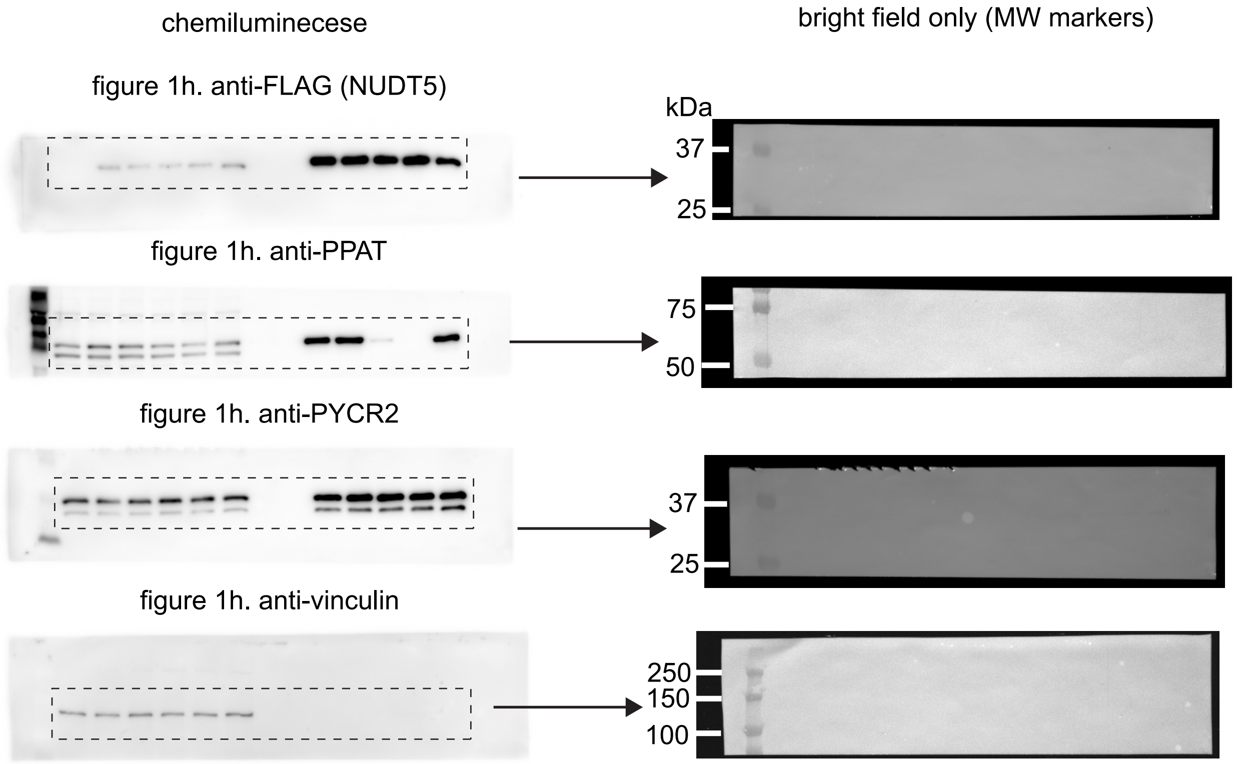

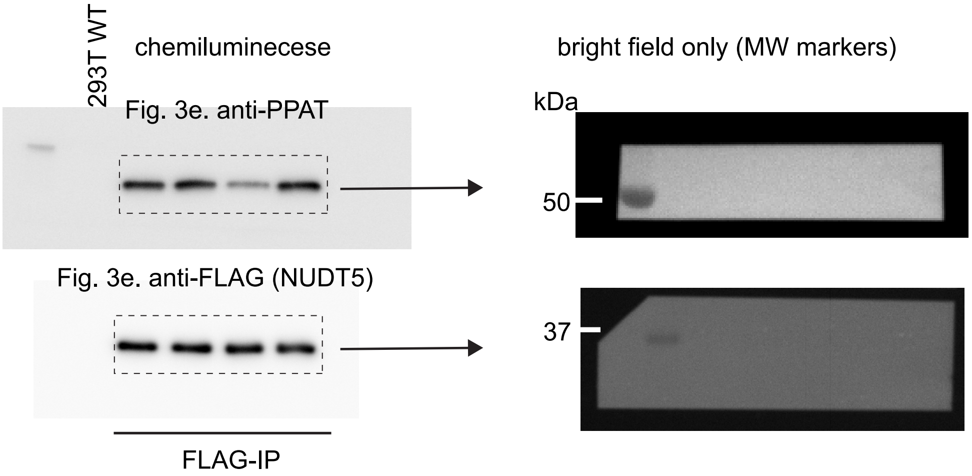
293T WT
chemiluminecese
bright field only (MW markers)
Fig. 3e. anti-PPAT
kDa
50
Fig. 3e. anti-FLAG (NUDT5)
37
FLAG-IP

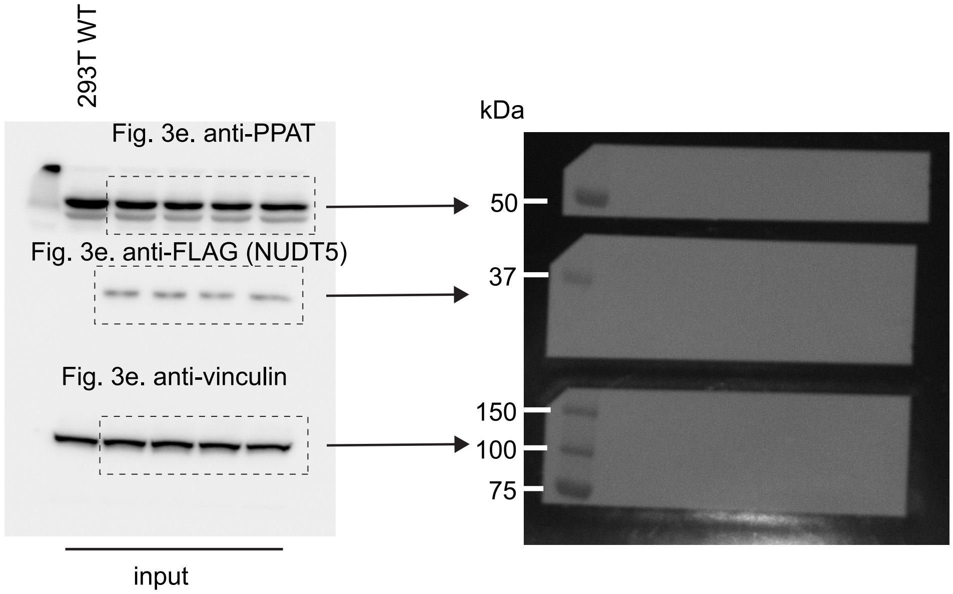
293T WT
kDa
Fig. 3e. anti-PPAT
50
Fig. 3e. anti-FLAG (NUDT5)
37
Fig. 3e. anti-vinculin
150
100
75
input

chemiluminecese

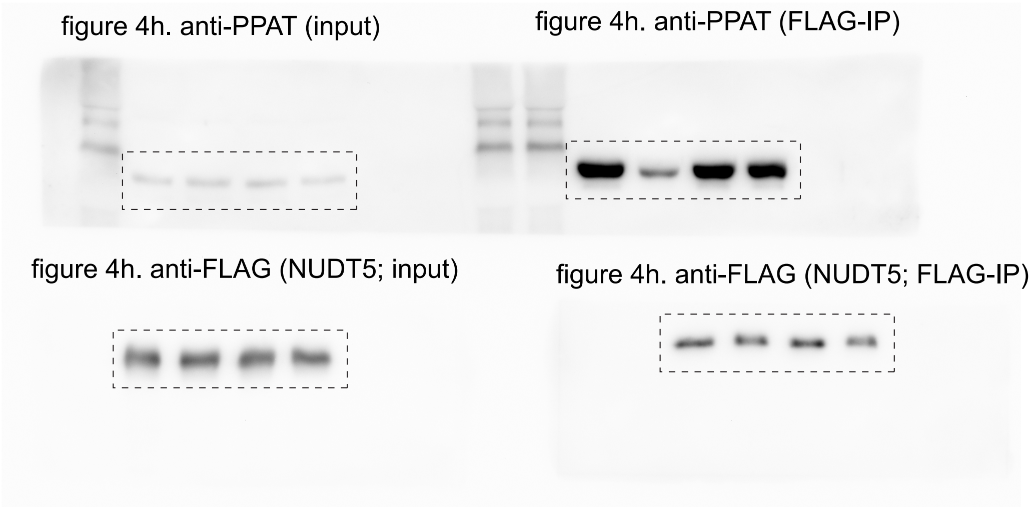

bright field only (MW markers)

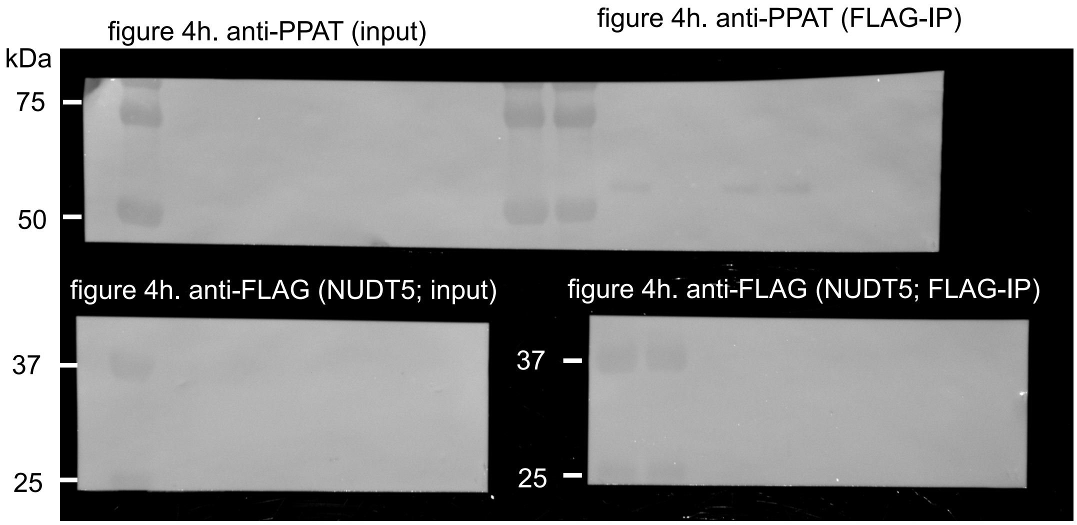

chemiluminecese

figure 4h. anti-vinculin (input)

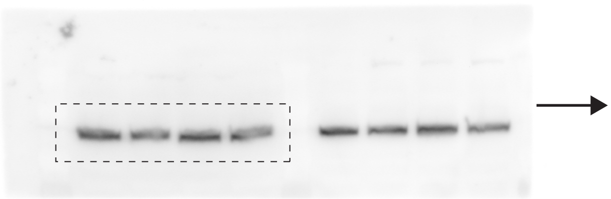

bright field only (MW markers)

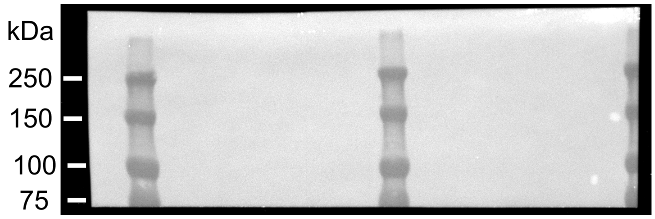

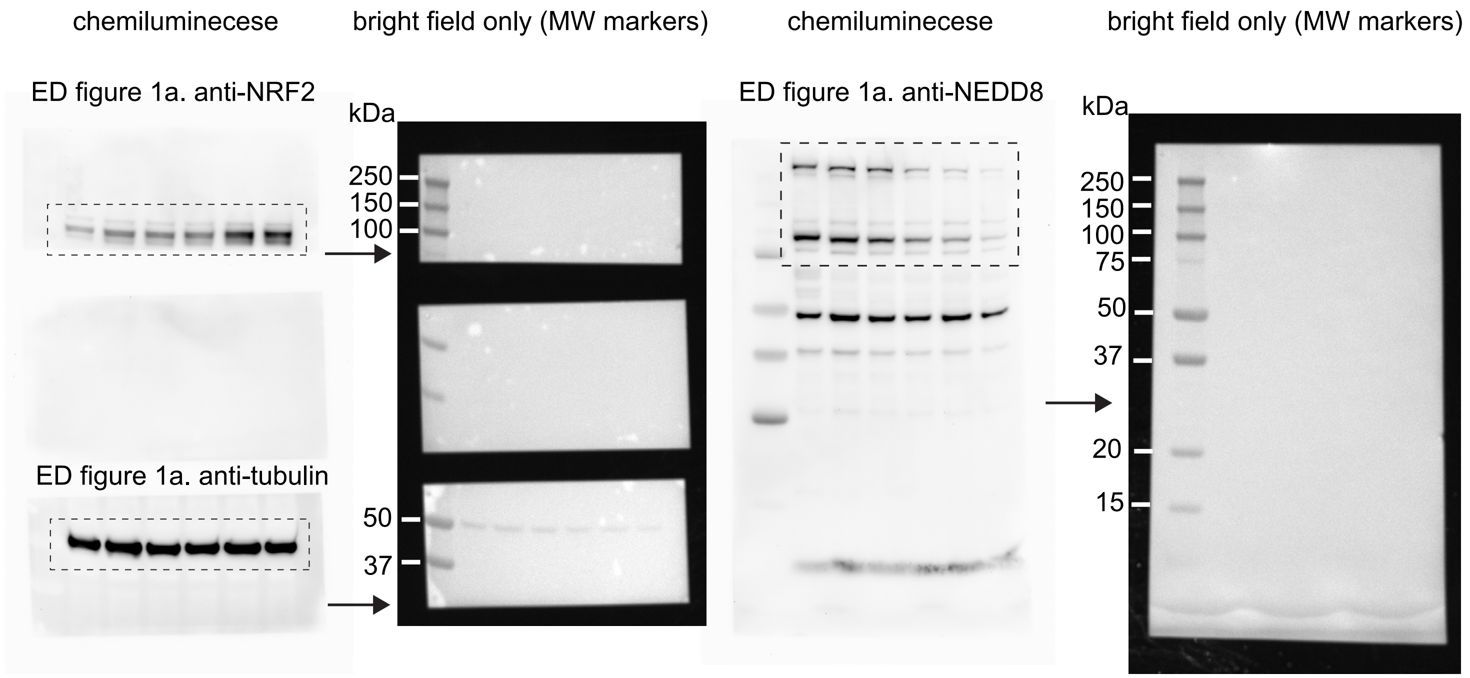

chemiluminecese
bright field only (MW markers)
chemiluminecese
bright field only (MW markers)
ED figure 1a. anti-NRF2
kDa
250
150
100
ED figure 1a. anti-tubulin
50
37
ED figure 1a. anti-NEDD8
kDa
250
150
100
75
50
37
20
15

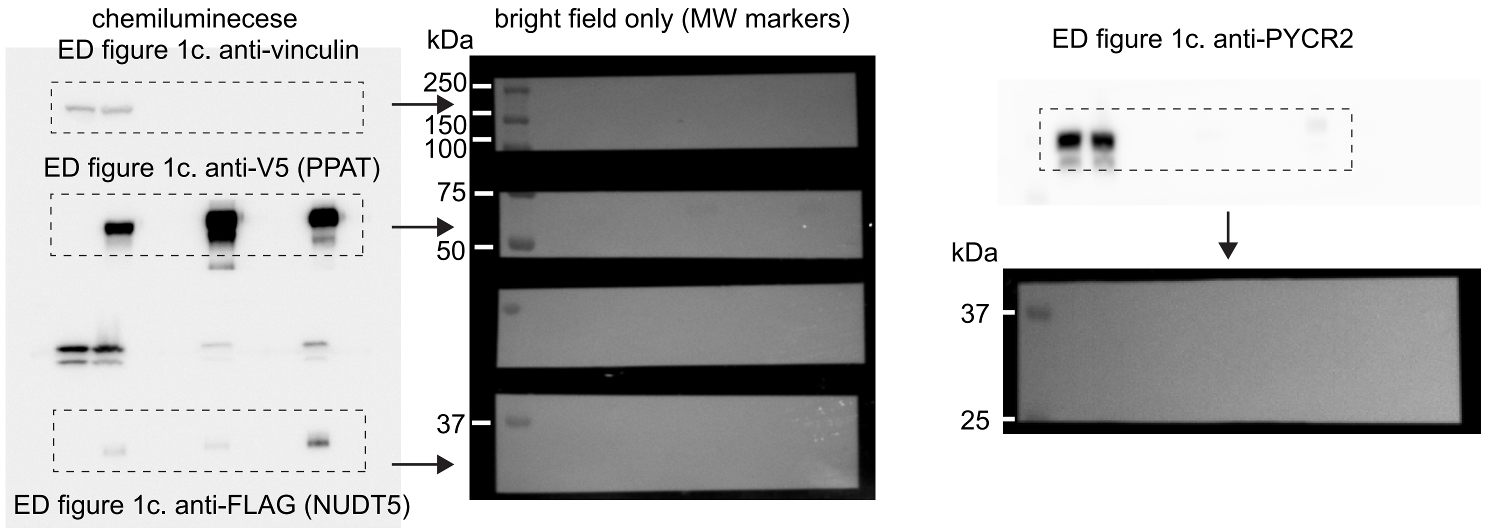

chemiluminecese
ED figure 1c. anti-vinculin
ED figure 1c. anti-V5 (PPAT)
ED figure 1c. anti-FLAG (NUDT5)
bright field only (MW markers)
kDa
250
150
100
75
50
37
ED figure 1c. anti-PYCR2
kDa
37
25

ED figure 3d - coomassie

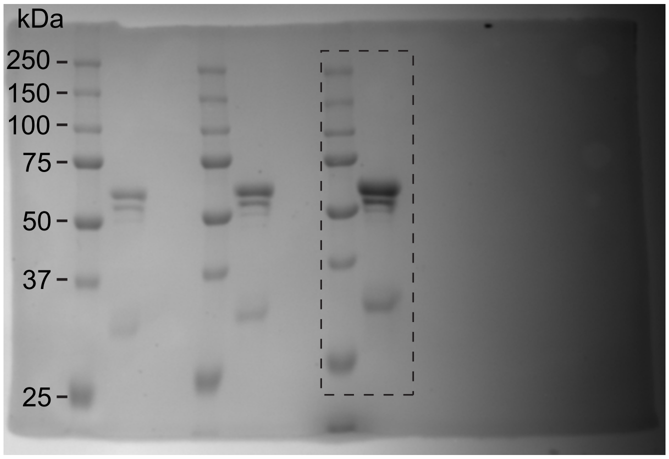

ED figure 3a - coomassie

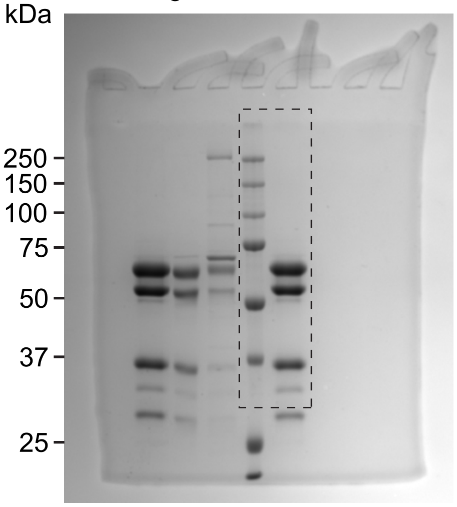

ED figure 3e - coomassie

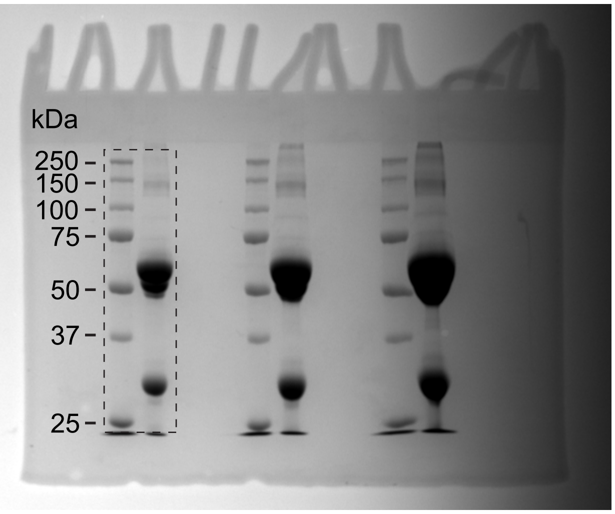

chemiluminecese

bright field only (MW markers)

ED figure 4j. anti-FLAG (NUDT5)

kDa

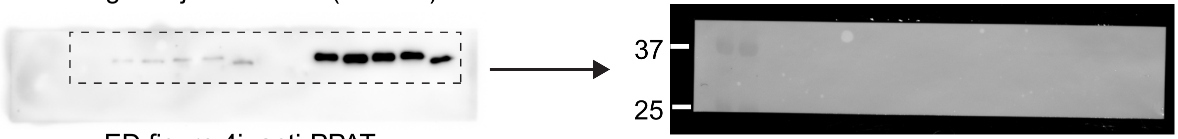

ED figure 4j. anti-PPAT

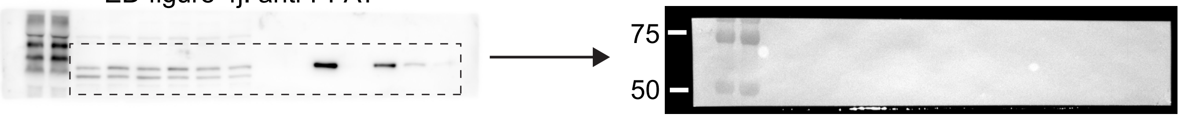

ED figure 4j. anti-PYCR2

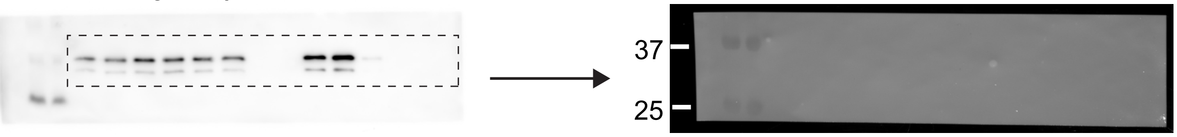

ED figure 4j. anti-vinculin

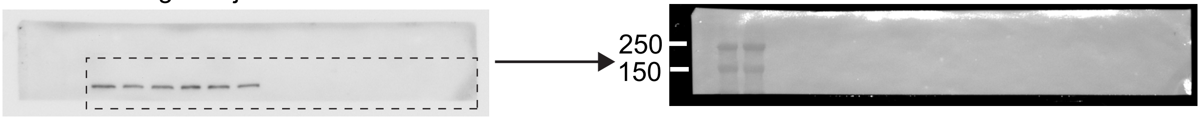

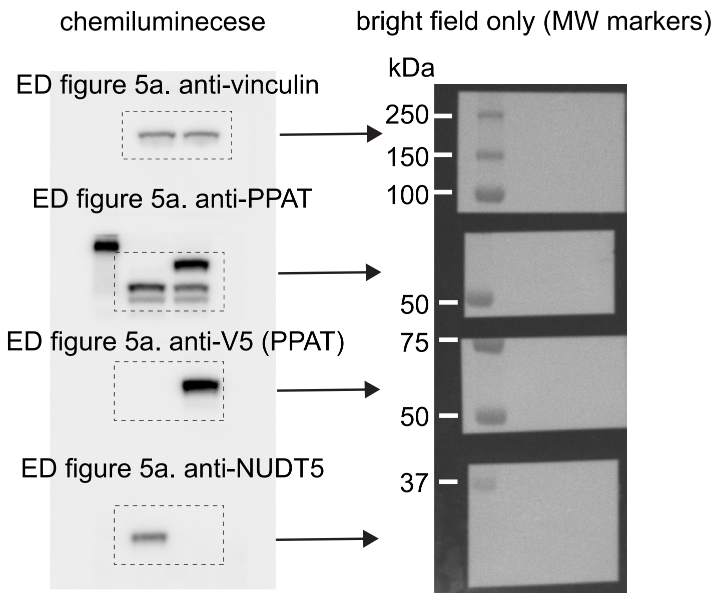
chemiluminecese
bright field only (MW markers)
ED figure 5a. anti-vinculin
ED figure 5a. anti-PPAT
ED figure 5a. anti-V5 (PPAT)
ED figure 5a. anti-NUDT5
kDa
250
150
100
50
75
50
37

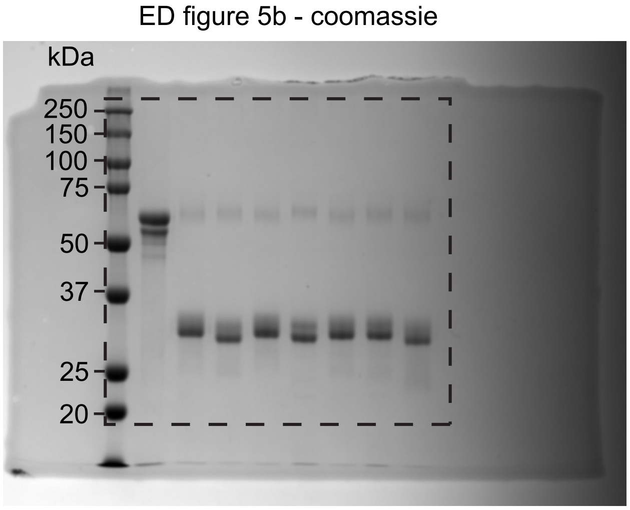
ED figure 5b - coomassie
kDa
250
150
100
75
50
37
25
20

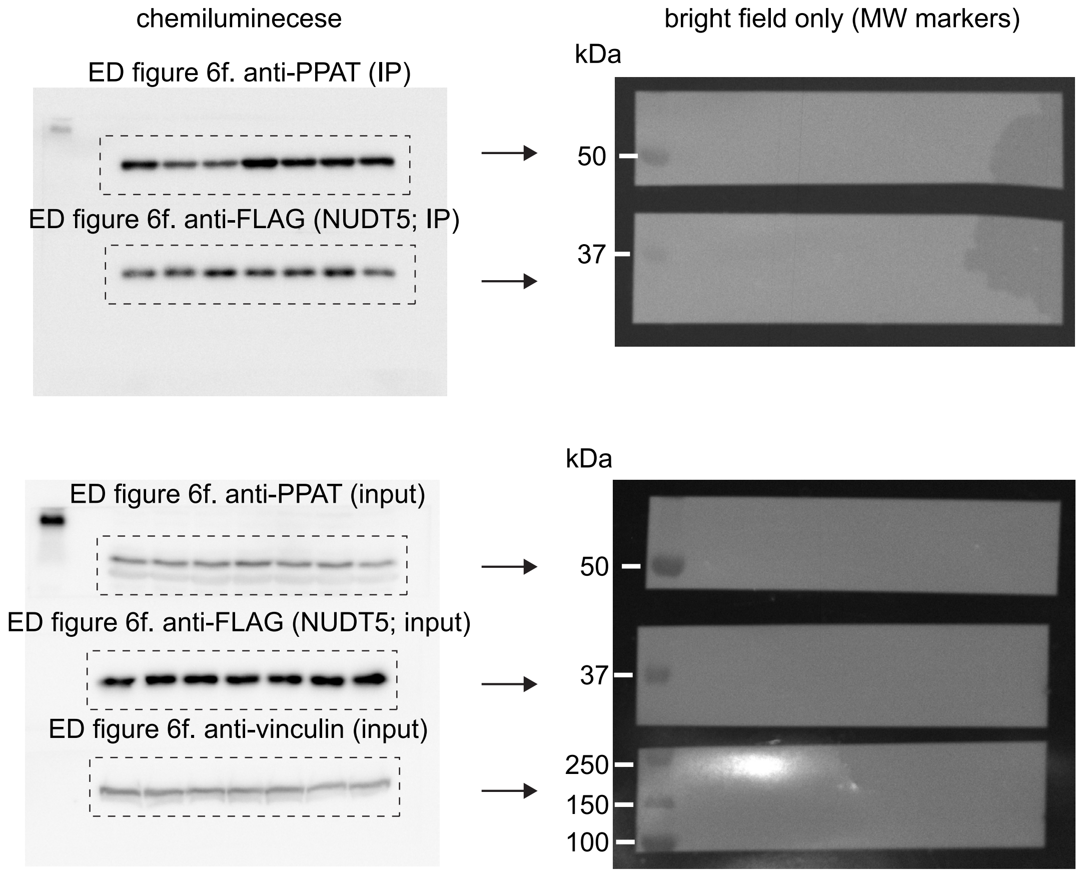
chemiluminecese
bright field only (MW markers)
ED figure 6f. anti-PPAT (IP)
ED figure 6f. anti-FLAG (NUDT5; IP)
kDa
50
37
ED figure 6f. anti-PPAT (input)
ED figure 6f. anti-FLAG (NUDT5; input)
ED figure 6f. anti-vinculin (input)
kDa
50
37
250
150
100

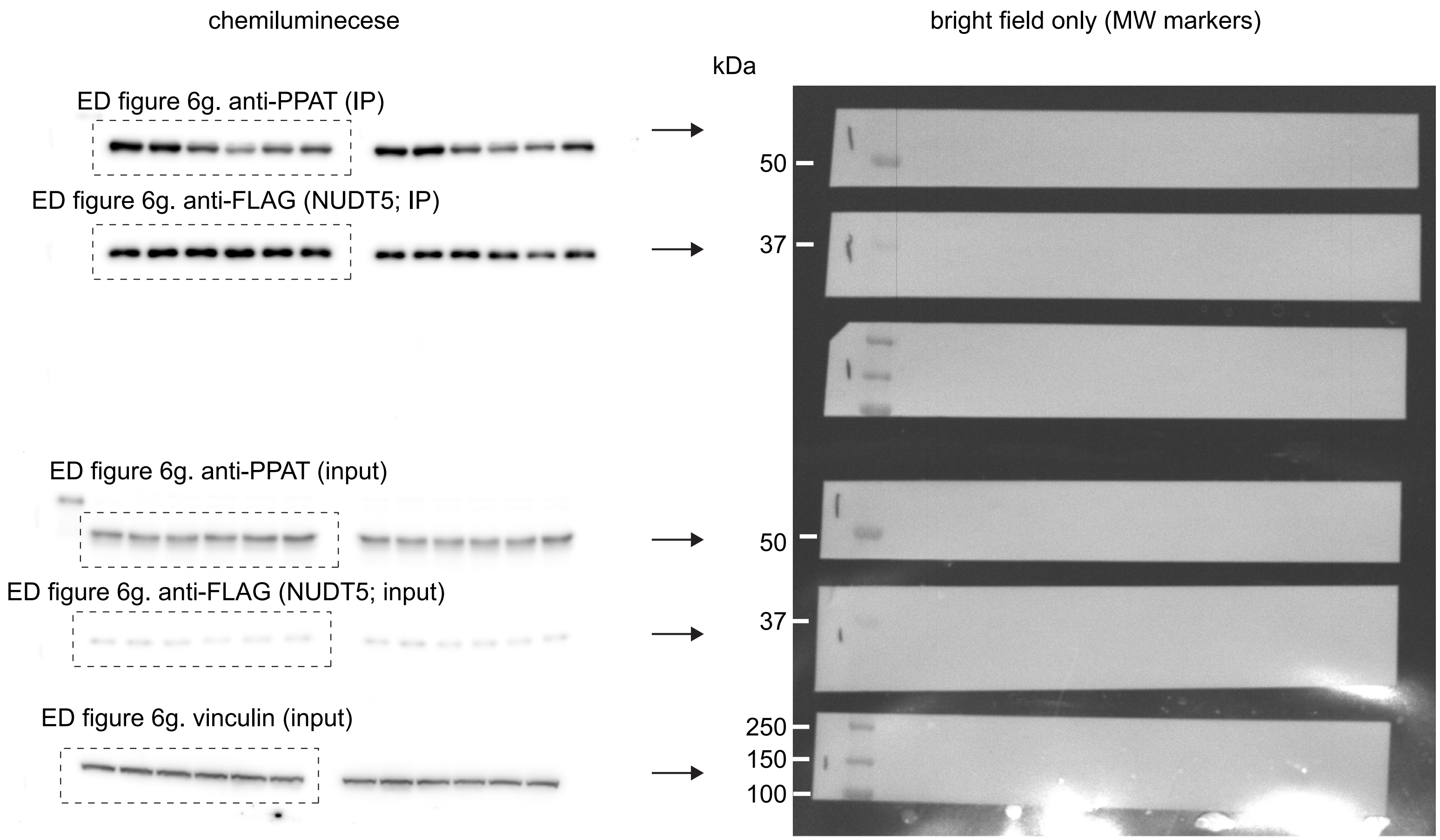
chemiluminecese
bright field only (MW markers)
kDa
ED figure 6g. anti-PPAT (IP)
ED figure 6g. anti-FLAG (NUDT5; IP)
ED figure 6g. anti-PPAT (input)
ED figure 6g. anti-FLAG (NUDT5; input)
ED figure 6g. vinculin (input)
50
37
50
37
250
150
100

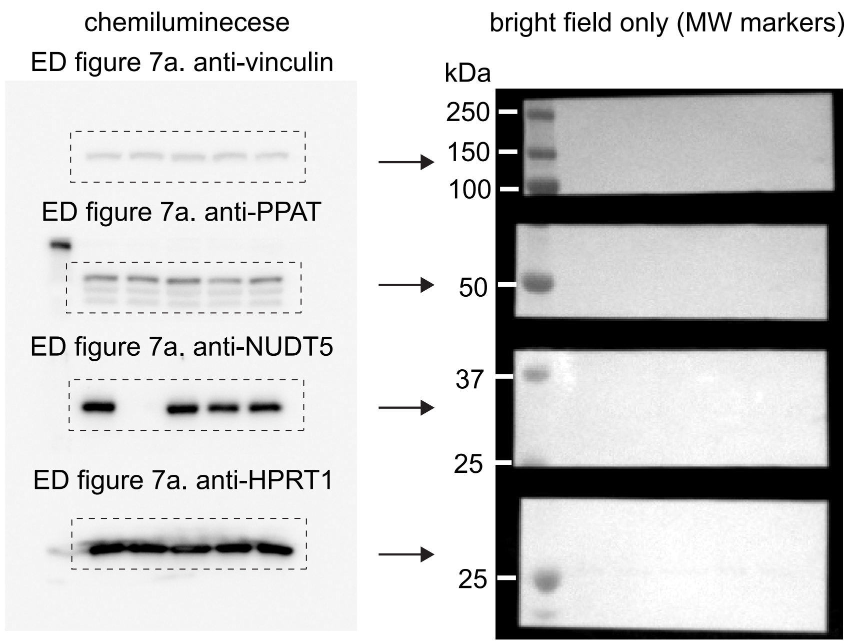
chemiluminecese
bright field only (MW markers)
kDa
ED figure 7a. anti-vinculin
ED figure 7a. anti-PPAT
ED figure 7a. anti-NUDT5
ED figure 7a. anti-HPRT1
250
150
100
50
37
25
25

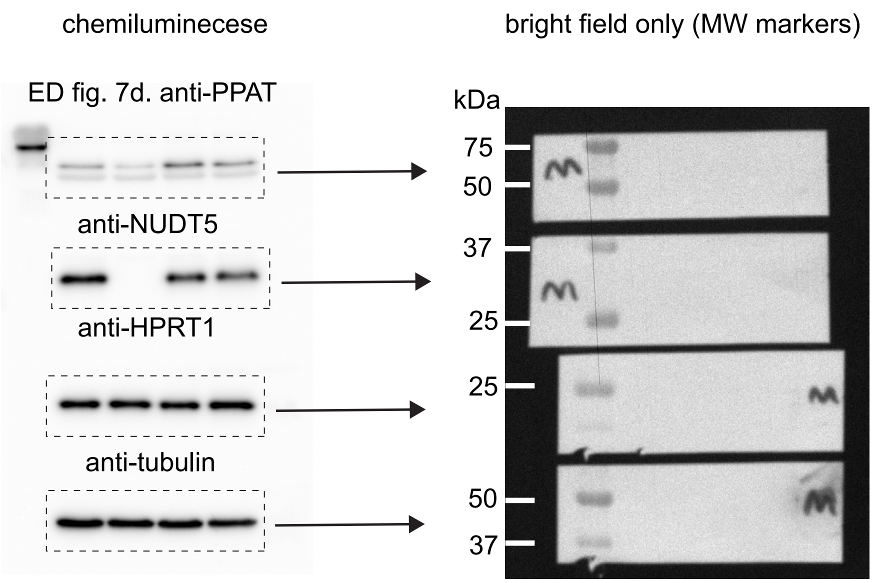
chemiluminecese
bright field only (MW markers)
ED fig. 7d. anti-PPAT
anti-NUDT5
anti-HPRT1
anti-tubulin
kDa
75
50
37
25
25
50
37
M
M
M
M

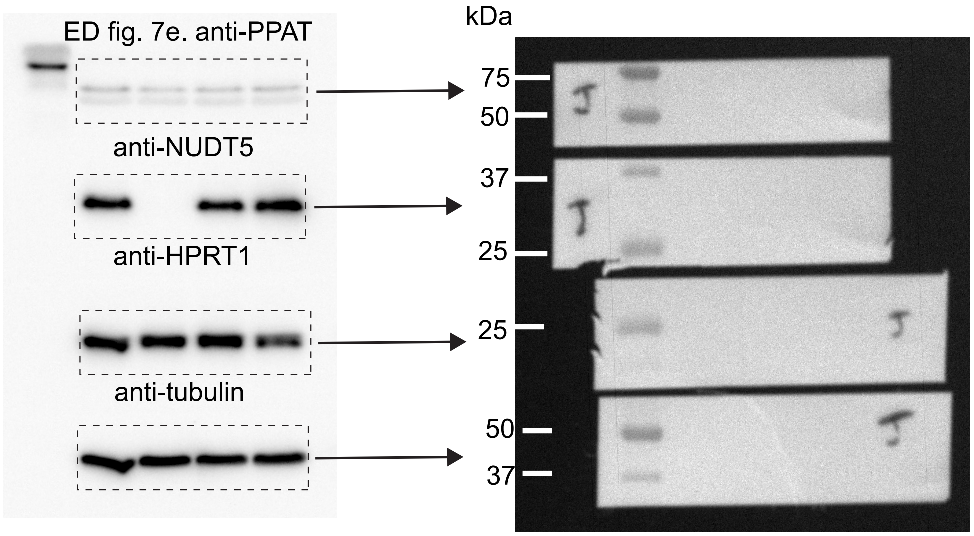
ED fig. 7e. anti-PPAT
anti-NUDT5
anti-HPRT1
anti-tubulin
kDa
75
50
37
25
25
50
37

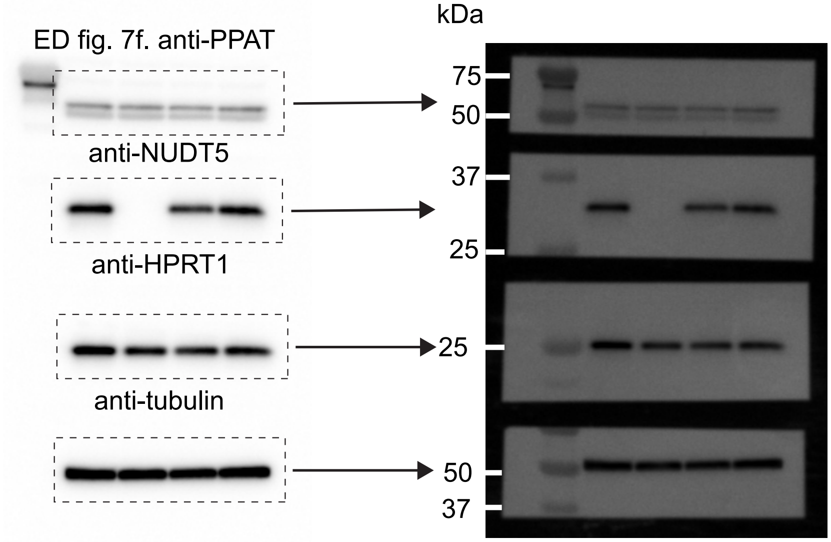
ED fig. 7f. anti-PPAT
anti-NUDT5
anti-HPRT1
anti-tubulin
kDa
75
50
37
25
25
50
37

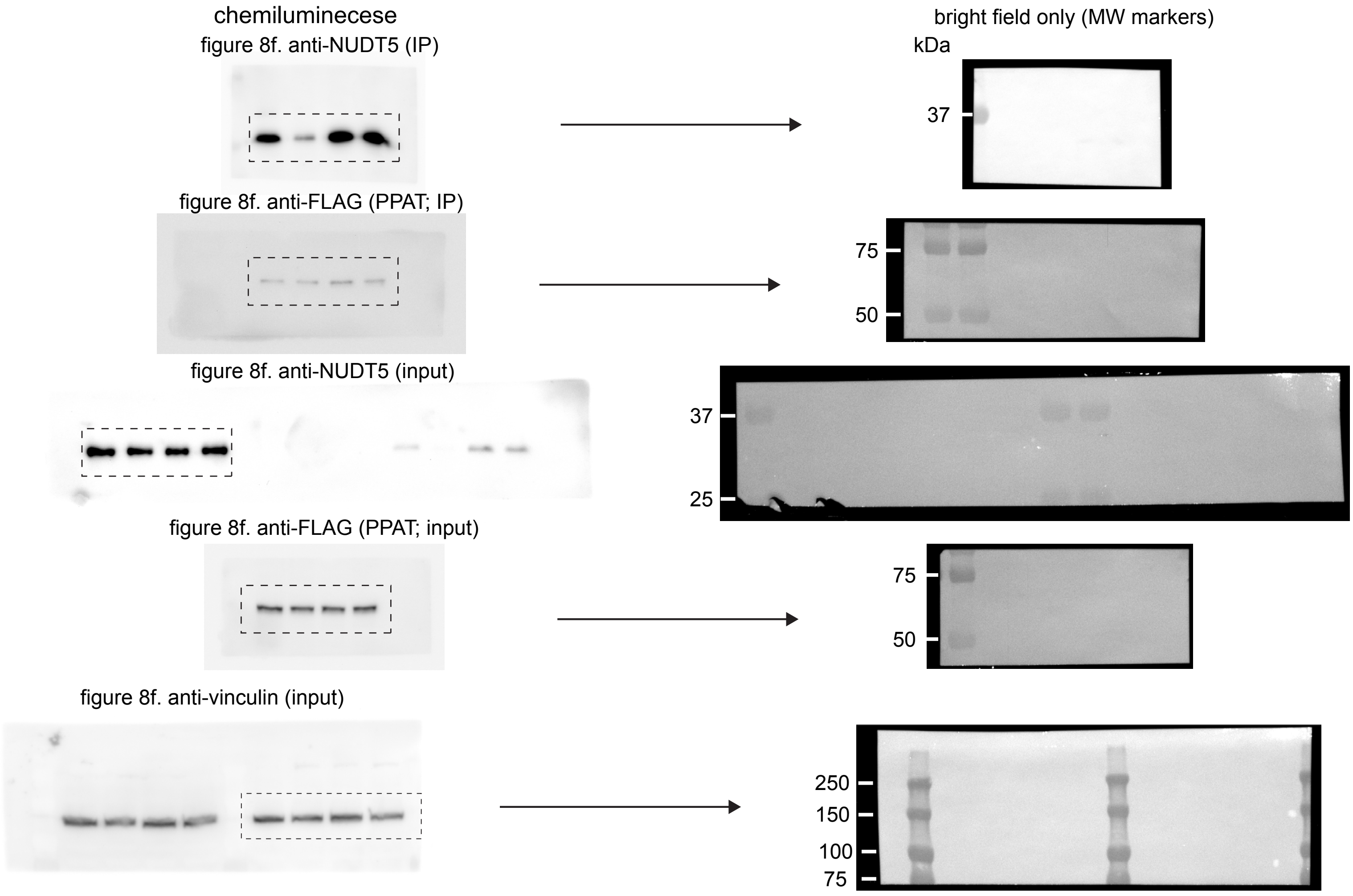
chemiluminecese
figure 8f. anti-NUDT5 (IP)
figure 8f. anti-FLAG (PPAT; IP)
figure 8f. anti-NUDT5 (input)
figure 8f. anti-FLAG (PPAT; input)
figure 8f. anti-vinculin (input)
bright field only (MW markers)
kDa
37
75
50
37
25
75
50
250
150
100
75

Supplementary Figure 2. FACS gating strategy for cell competition growth experiments.

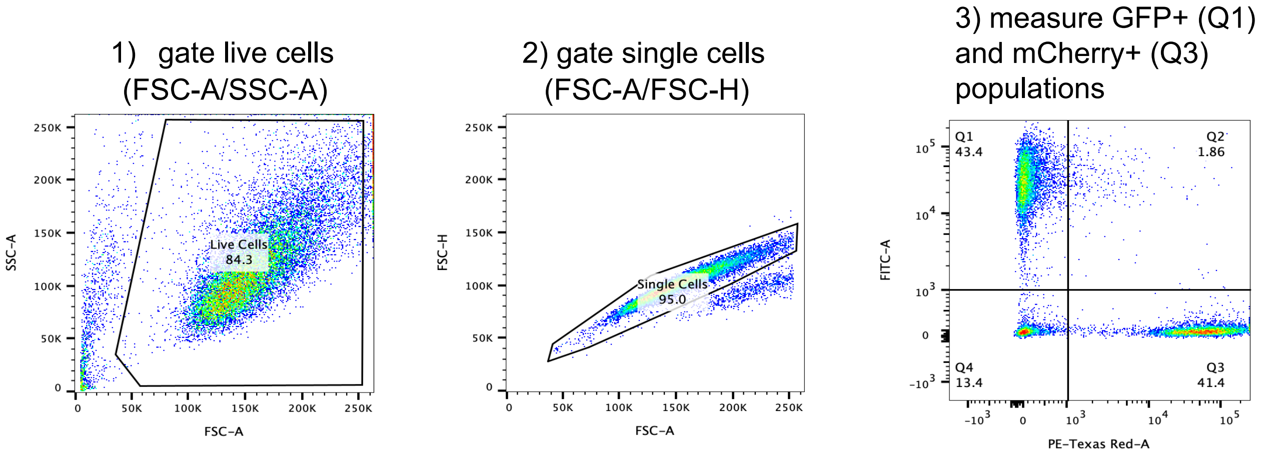

Supplementary Figure 3. Supporting data for custom synthesized compounds. Data contain LC-MS and NMR analysis of 6-ethylTIMP (denoted SW-01) and 6-benzylTIMP (denoted SW-05).

Print Time: 5/9/2025 12:32:31 P

LCMS REPORT

Compound ID	:	SW-01
Sample ID	:	ET89462-1107-P1A
Injection Date	:	09. May. 2025
Location	:	P2-B-03
Inj. Vol.	:	1.00 uL
Acq Method	:	D:\DATA\2505\250509 11\5_95AB_6min_220&254_ELSD.M
Data Filename	:	D:\DATA\2505\250509 11\ET89462-1107-P1A.D
Instrument	:	02-LCMS-BX

Chromatogram

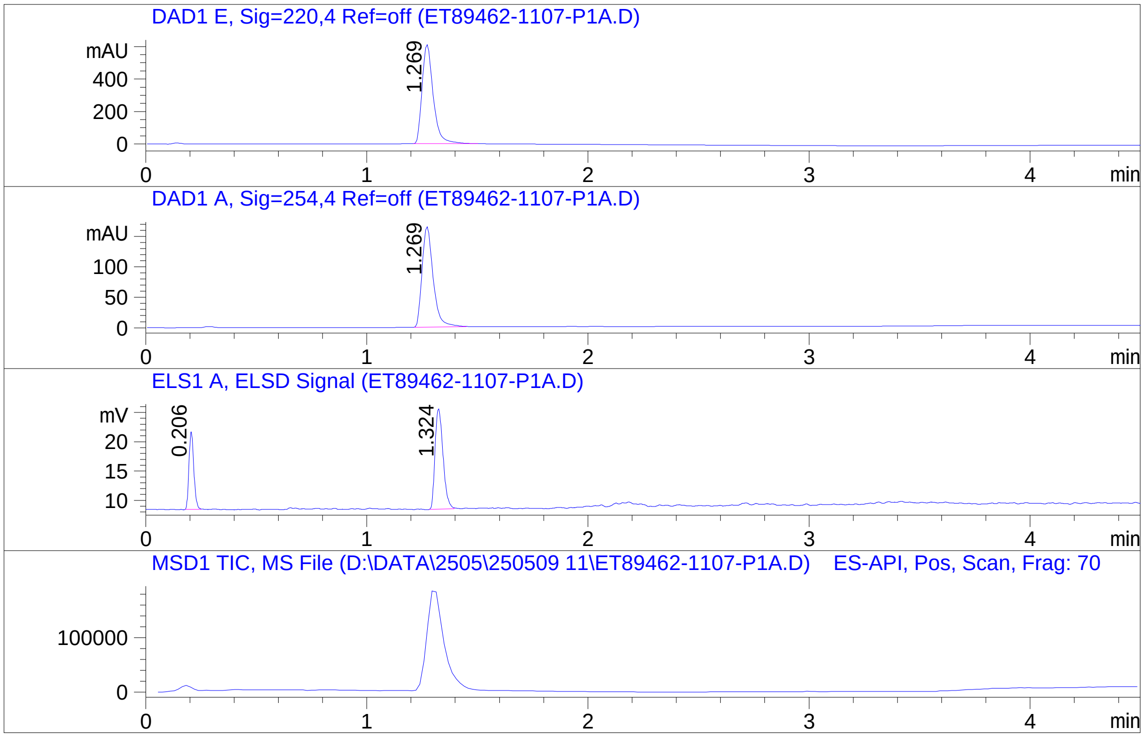

Integration Result

Signal 1 : DAD1 E, Sig=220,4 Ref=off

Peak #	RT [min]	Height	Height %	Width [min]	Area	Area %
1	1.269	610.012	100.000	0.053	2149.241	100.000

Signal 2 : DAD1 A, Sig=254,4 Ref=off

Peak #	RT [min]	Height	Height %	Width [min]	Area	Area %
1	1.269	164.701	100.000	0.053	576.793	100.000

Signal 3 : ELS1 A, ELSD Signal

Peak #	RT [min]	Height	Height %	Width [min]	Area	Area %
1	0.206	13.265	43.644	0.024	20.198	31.911
2	1.324	17.128	56.356	0.038	43.095	68.089

Signal 4 : MSD1 TIC, MS File

Peak #	RT [min]	Height	Height %	Width [min]	Area	Area %

MS Spectrum

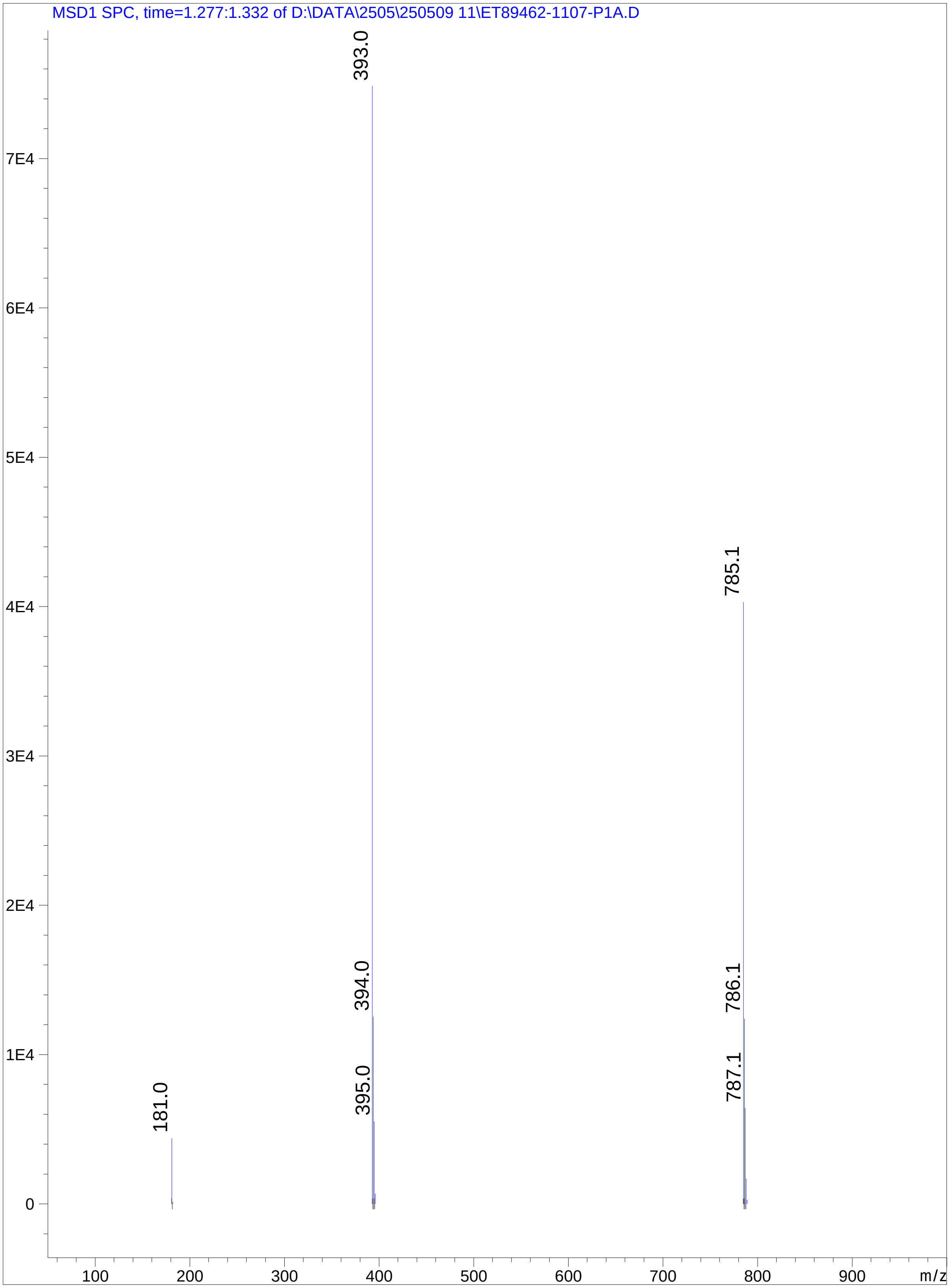

MSD1 SPC, time=1.277:1.332 of D:\DATA\2505\250509 11\ET89462-1107-P1A.D
393.0
785.1
394.0
786.1
395.0
787.1
181.0
7E4
6E4
5E4
4E4
3E4
2E4
1E4
0
100
200
300
400
500
600
700
800
900
m/z

Compound ID: SW-01

ET89462-1107-P1B D2O Bruker_02_F_400MHz

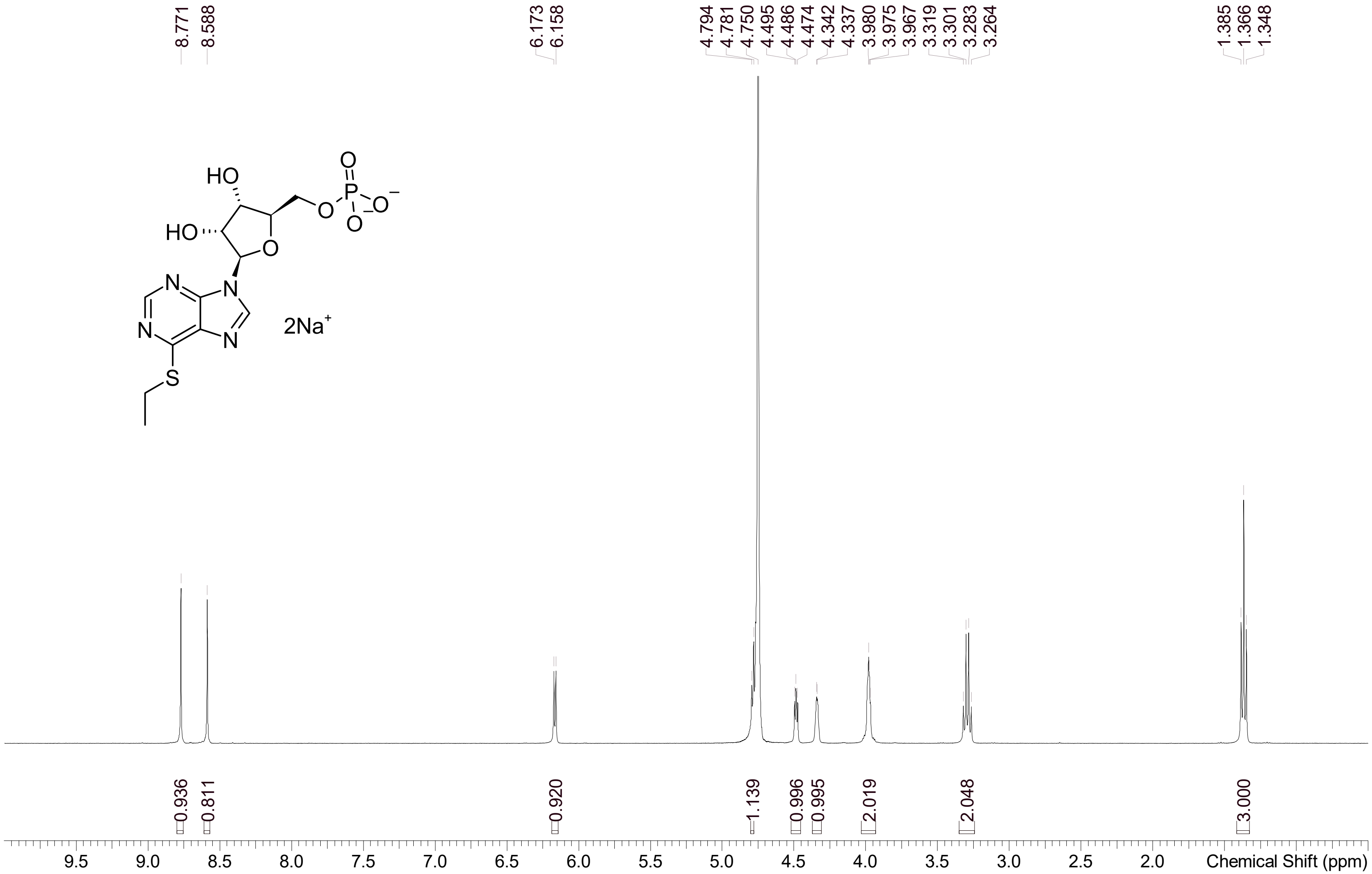

Acquisition Time (sec)	2.0447
Comment	ET89462-1107-P1B D2O Bruker_02_F_400MHz
Date	09 May 2025 10:35:29 (GMT+08:00)
Frequency (MHz)	400.1700
Nucleus	1H
Number of Transients	8
Origin	spect
Original Points Count	16384
Owner	nmr
Points Count	65536
Pulse Sequence	zg30
Receiver Gain	177.77
SW(cyclical) (Hz)	8012.82
Solvent	DEUTERIUM OXIDE
Spectrum Offset (Hz)	2489.8176
Spectrum Type	standard
Sweep Width (Hz)	8012.70
Temperature (degree C)	24.999

Operator:

Date:

Compound ID: SW-01

ET89462-1107-P1C D2O Bruker_02_B_400MHz 13C-NMR

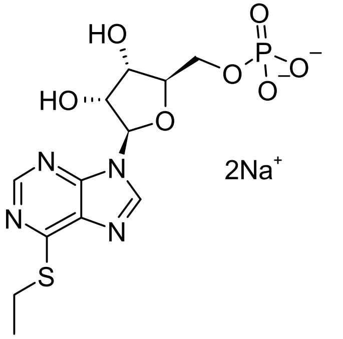

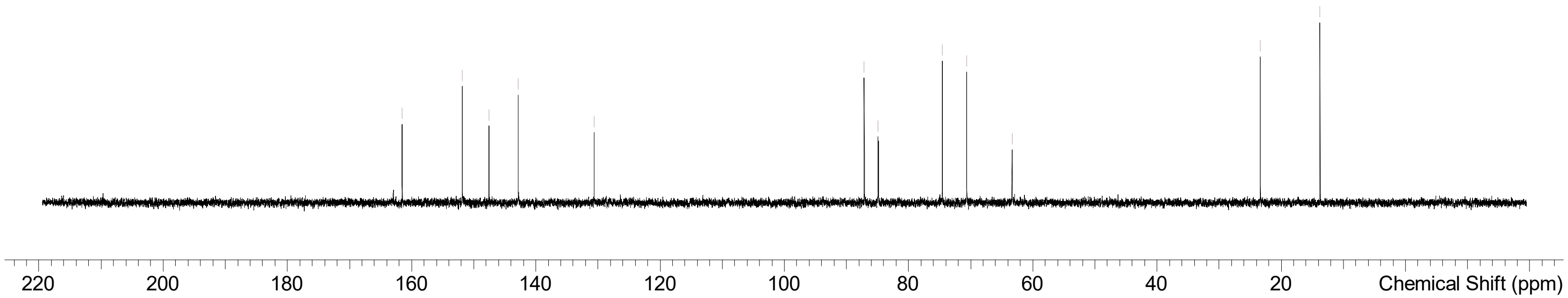

Acquisition Time (sec)	1.3631
Comment	ET89462-1107-P1C D2O Bruker_02_B_400MHz 13C-NMR
Date	10 May 2025 00:01:41 (GMT+08:00)
Frequency (MHz)	100.6128
Nucleus	13C
Number of Transients	4096
Origin	spect
Original Points Count	32768
Owner	nmr
Points Count	65536
Pulse Sequence	zgpg30
Receiver Gain	199.37
SW(cyclical) (Hz)	24038.46
Solvent	DEUTERIUM OXIDE
Spectrum Offset (Hz)	10060.8057
Spectrum Type	standard
Sweep Width (Hz)	24038.09
Temperature (degree C)	-273.000

Operator: Date:

LCMS REPORT

Compound ID	:	SW-05
Sample ID	:	ET89462-1139-P1A
Injection Date	:	21. May. 2025
Location	:	P2-A-05
Inj. Vol.	:	1.00 uL
Acq Method	:	D:\DATA\2505\250521 4\5_95AB_6min_220&254_ELSD.M
Data Filename	:	D:\DATA\2505\250521 4\ET89462-1139-P1A.D
Instrument	:	02-LCMS-BX

Chromatogram

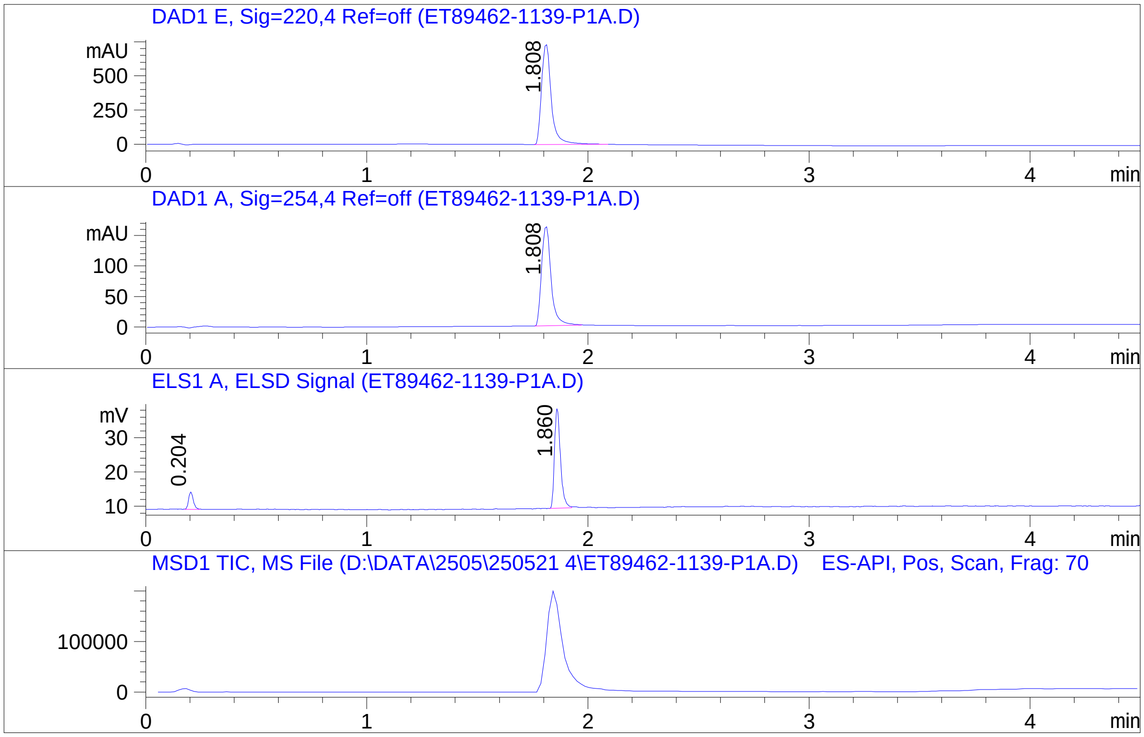

Integration Result

Signal 1 : DAD1 E, Sig=220,4 Ref=off

Peak #	RT [min]	Height	Height %	Width [min]	Area	Area %
1	1.808	736.054	100.000	0.048	2268.621	100.000

Signal 2 : DAD1 A, Sig=254,4 Ref=off

Peak #	RT [min]	Height	Height %	Width [min]	Area	Area %
1	1.808	163.393	100.000	0.047	491.454	100.000

Signal 3 : ELS1 A, ELSD Signal

Peak #	RT [min]	Height	Height %	Width [min]	Area	Area %
1	0.204	5.029	14.792	0.023	7.468	11.998
2	1.860	28.971	85.208	0.030	54.773	88.002

Signal 4 : MSD1 TIC, MS File

Peak #	RT [min]	Height	Height %	Width [min]	Area	Area %

MS Spectrum

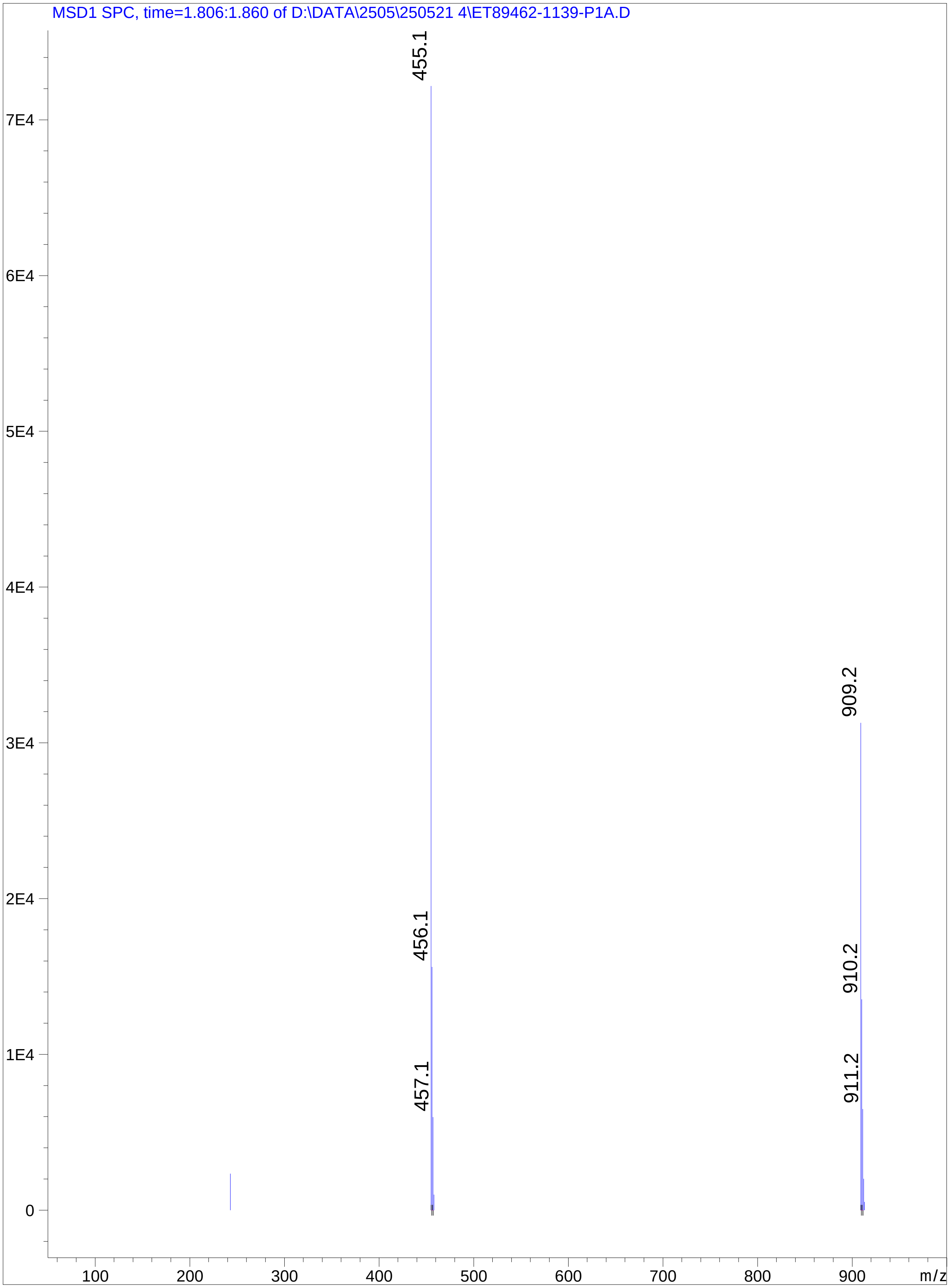
MSD1 SPC, time=1.806:1.860 of D:\DATA\2505\250521 4\ET89462-1139-P1A.D
455.1
456.1
457.1
909.2
910.2
911.2
7E4
6E4
5E4
4E4
3E4
2E4
1E4
0
100
200
300
400
500
600
700
800
900
m/z

Compound ID: SW-05

ET89462-1139-P1B D2O Bruker_02_C_400MHz

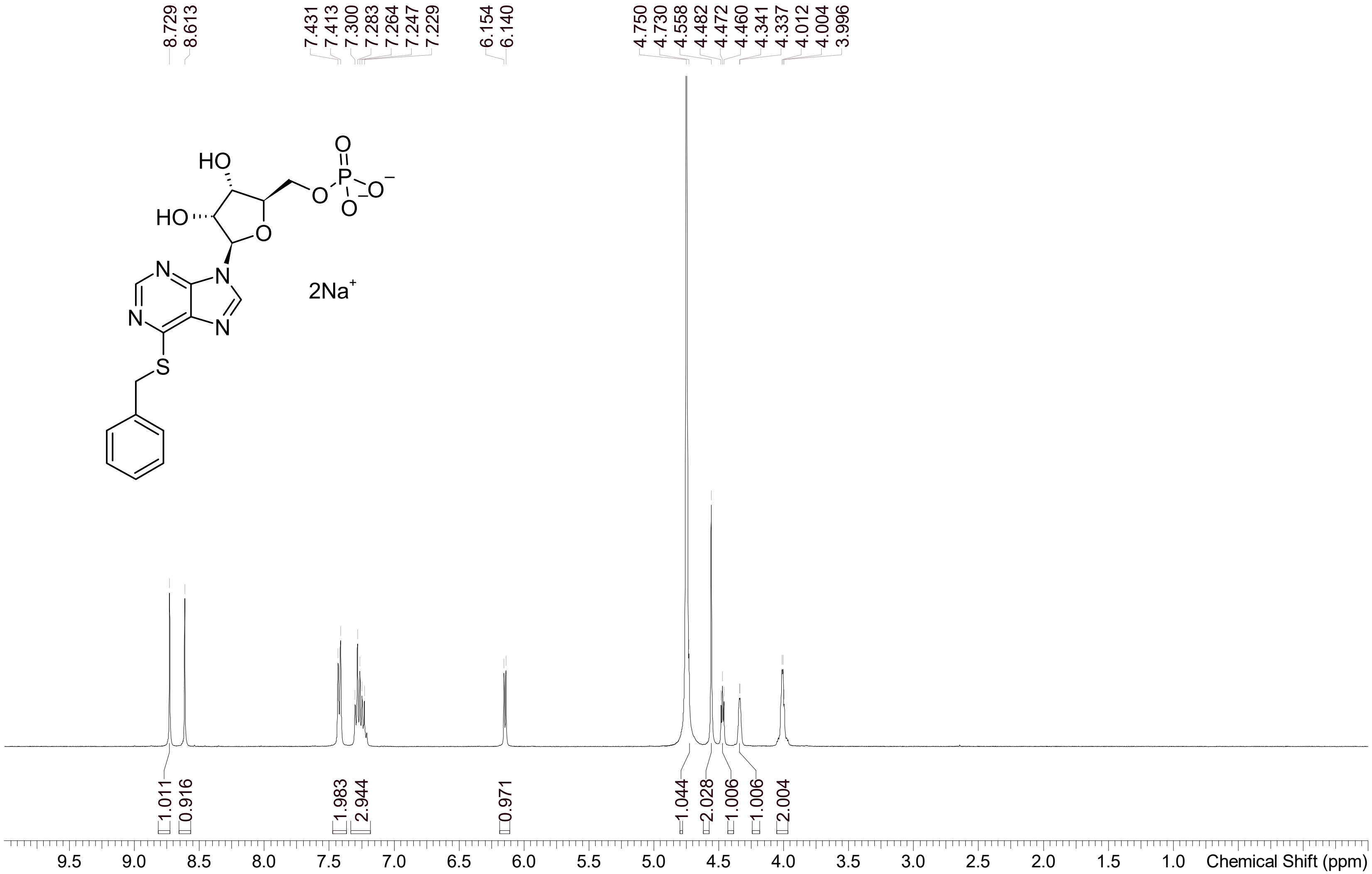

Acquisition Time (sec)	2.0447
Comment	ET89462-1139-P1B D2O Bruker_02_C_400MHz
Date	21 May 2025 09:31:04 (GMT+08:00)
Frequency (MHz)	400.1500
Nucleus	1H
Number of Transients	8
Origin	spect
Original Points Count	16384
Owner	nmr
Points Count	65536
Pulse Sequence	zg30
Receiver Gain	106.30
SW(cyclical) (Hz)	8012.82
Solvent	DEUTERIUM OXIDE
Spectrum Offset (Hz)	2490.8132
Spectrum Type	standard
Sweep Width (Hz)	8012.70
Temperature (degree C)	25.164

Operator:

Date:

Compound ID: SW-05

ET89462-1139-P1C D2O Bruker_02_M_400MHz 13C-NMR

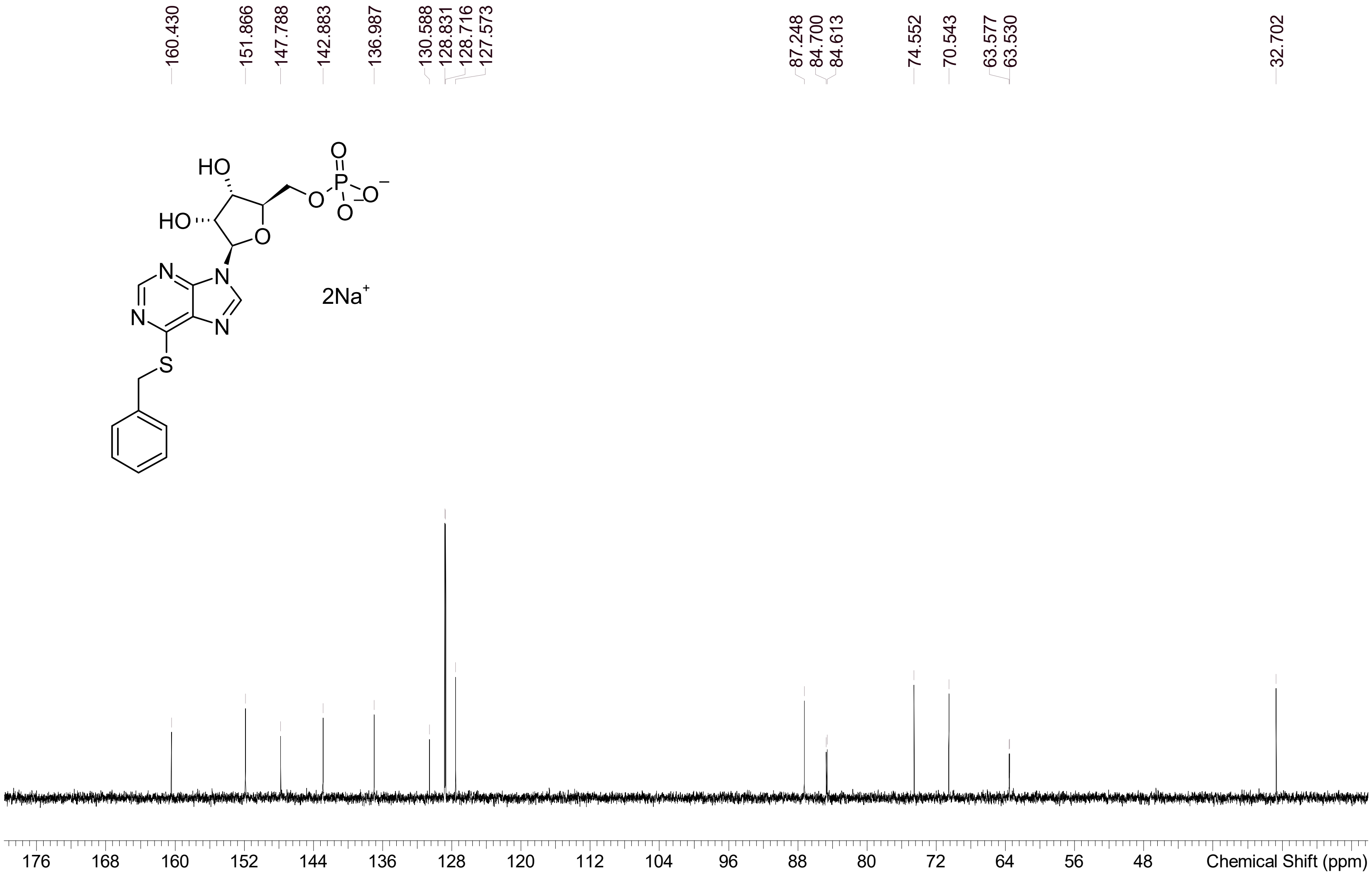

Acquisition Time (sec)	1.3763
Comment	ET89462-1139-P1C D2O Bruker_02_M_400MHz 13C-NMR
Date	22 May 2025 00:05:38 (GMT+08:00)
Frequency (MHz)	100.4996
Nucleus	13C
Number of Transients	2048
Origin	Avance
Original Points Count	32768
Owner	nmr
Points Count	65536
Pulse Sequence	zgpg30
Receiver Gain	16.25
SW(cyclical) (Hz)	23809.52
Solvent	DEUTERIUM OXIDE
Spectrum Offset (Hz)	10049.9629
Spectrum Type	standard
Sweep Width (Hz)	23809.16
Temperature (degree C)	25.165

Operator:

Date:

Supplementary Table 1. Compound identification for targeted metabolomics.

Molecule	Isotopomer	Chemical Formula*	Retention Time (min) Average	Retention Time (min) Standard Deviation	Ion Polarity	Precursor Ion (m/z)	Product Ion (m/z)	Collision Energy (eV)	Fragmentor (V)	Cell Accelerator Voltage (V)
PRPP	M+0	C5H13O14P3	20.59	0.12	Negative	388.9	290.9	9	106	4
ADP-ribose	M+0	C15H23N5O14P2	20.06	0.13	Positive	560	136	25	175	4
ADP-ribose	M+2	C15H23N'2N3O14P2	20.06	0.13	Positive	562	138	25	175	4
Ribose-5P	M+0	C5H11O8P	20.49	0.12	Negative	229	96.9	4	91	4
IMP	M+0	C10H13N4O8P	18.78	0.12	Positive	349.1	137	8	96	5
IMP	M+2	C10H13N'2N2O8P	18.78	0.12	Positive	351.1	139	8	96	5
IDP	M+0	C10H14N4O11P2	20.38	0.14	Positive	429	137	21	166	4
IDP	M+2	C10H14N'2N2O11P2	20.38	0.14	Positive	431	139	21	166	4
ITP	M+0	C10H15N4O14P3	21.72	0.16	Positive	509	137	21	166	3
ITP	M+2	C10H15N4O14P3	21.72	0.16	Positive	511	139	21	166	3
GMP	M+0	C10H14N5O8P	21.36	0.16	Positive	364.1	152	12	91	3
GMP	M+2	C10H14N'2N3O8P	21.36	0.16	Positive	366.1	154	12	91	3
GMP	M+3	C10H14N'3N2O8P	21.36	0.16	Positive	367.1	155	12	91	3
GDP	M+0	C10H15N5O11P2	22.99	0.17	Positive	444	152	17	166	5
GDP	M+2	C10H15N'2N3O11P2	22.99	0.17	Positive	446	154	17	166	5
GDP	M+3	C10H15N'3N2O11P2	22.99	0.17	Positive	447	155	17	166	5
GTP	M+0	C10H16N5O14P3	24.26	0.17	Positive	524	151.9	25	166	5
GTP	M+2	C10H16N'2N3O14P3	24.26	0.17	Positive	526	153.9	25	166	5
GTP	M+3	C10H16N'3N2O14P3	24.26	0.17	Positive	527	154.9	25	166	5
AMP	M+0	C10H14N5O7P	18.76	0.12	Positive	348.1	136	16	102	4
AMP	M+2	C10H14N'2N3O7P	18.76	0.12	Positive	350.1	138	16	102	4
ADP	M+0	C10H15N5O10P2	20.36	0.12	Positive	428	136	33	166	4
ADP	M+2	C10H15N'2N3O10P2	20.36	0.12	Positive	430	138	33	166	4
ATP	M+0	C10H16N5O13P3	21.63	0.20	Positive	508	136.1	41	166	3
ATP	M+2	C10H16N'2N3O13P3	21.63	0.20	Positive	510	138.1	41	166	3
G6P	Internal Isotopic Standard	C'6H13O9P	21.61	0.16	Negative	265.1	97	8	96	5
F16BP	Internal Isotopic Standard	C'1C5H14O12P2	23.63	0.18	Negative	340.1	96.9	20	96	4

*In chemical formula, ' denotes heavy isotope of the preceeding element. For example, C' denotes 13C and N' denotes 15N.
