# Supplementary material for: Metabolite glues as a means of purine sensing and chemotherapeutic response
Source: Nature. 2026 Jul 15;655(8125):1300–8. doi: 10.1038/s41586-026-10790-3 (PMC13421311; doi:10.1038/s41586-026-10790-3)
Supplement: Supplementary file 2 — Reporting Summary [file 41586_2026_10790_MOESM2_ESM.pdf]

nature portfolio

Corresponding author(s): Michael Rapé

Last updated by author(s): May 9, 2026

Reporting Summary

Nature Portfolio wishes to improve the reproducibility of the work that we publish. This form provides structure for consistency and transparency in reporting. For further information on Nature Portfolio policies, see our Editorial Policies and the Editorial Policy Checklist.

Statistics

For all statistical analyses, confirm that the following items are present in the figure legend, table legend, main text, or Methods section.

n/a	Confirmed	
☐	☒	The exact sample size (*n*) for each experimental group/condition, given as a discrete number and unit of measurement
☐	☒	A statement on whether measurements were taken from distinct samples or whether the same sample was measured repeatedly
☐	☒	The statistical test(s) used AND whether they are one- or two-sided<br>*Only common tests should be described solely by name; describe more complex techniques in the Methods section.*
☐	☒	A description of all covariates tested
☐	☒	A description of any assumptions or corrections, such as tests of normality and adjustment for multiple comparisons
☐	☒	A full description of the statistical parameters including central tendency (e.g. means) or other basic estimates (e.g. regression coefficient) AND variation (e.g. standard deviation) or associated estimates of uncertainty (e.g. confidence intervals)
☐	☒	For null hypothesis testing, the test statistic (e.g. *F*, *t*, *r*) with confidence intervals, effect sizes, degrees of freedom and *P* value noted<br>*Give P values as exact values whenever suitable.*
☒	☐	For Bayesian analysis, information on the choice of priors and Markov chain Monte Carlo settings
☒	☐	For hierarchical and complex designs, identification of the appropriate level for tests and full reporting of outcomes
☒	☐	Estimates of effect sizes (e.g. Cohen's *d*, Pearson's *r*), indicating how they were calculated

Our web collection on statistics for biologists contains articles on many of the points above.

Software and code

Policy information about availability of computer code

Data collection	N/A
Data analysis	All software used is freely/commercially available: FACSDiva (Version 9.0), FlowJo (Version 10.10.0), GraphPad Prism (Version 10), SerialEM (Version 4.1), AlphaFold 3, Coot (Version 0.9.8.92), ChimeraX (Version 1.8), PyMOL (Version 2.5.5), PHENIX (Version 1.21.1-5286), cryoSPARC (Version 4.3), Isolde (Version 1.2), Spectronaut (Version 18.0), Agilent MassHunter (Version 10.1), Skyline (MacCoss Lab version 24.1.0.214), Xcalibur (Version 4.3). Python (v3.10), SciPy library (v1.11), Matplotlib (v3.7), Refeyn AquireMP (2024 R2), Refeyn DiscoverMP (2024 R2), IsoCorrectoR (version 3.22).

For manuscripts utilizing custom algorithms or software that are central to the research but not yet described in published literature, software must be made available to editors and reviewers. We strongly encourage code deposition in a community repository (e.g. GitHub). See the Nature Portfolio guidelines for submitting code & software for further information.

Data

Policy information about availability of data

All manuscripts must include a data availability statement. This statement should provide the following information, where applicable:

- Accession codes, unique identifiers, or web links for publicly available datasets
- A description of any restrictions on data availability
- For clinical datasets or third party data, please ensure that the statement adheres to our policy

Structural models of PPAT-NUDT5-nucleotide complexes have been deposited to the Worldwide Protein Data Bank (wwPBD) and Electron Microscopy Data Bank (EMDB) with the following accession codes: AMP complex (PDB: 9Q0M; EMDB: EMD-72099), 6-meTIMP complex (PDB: 9Q0N; EMDB: EMD-72100), 6-benzylTIMP complex (PDB: 9Q0O; EMDB: EMD-72101). Numerical and immunoblot source data are provided as supplementary data with this manuscript. The mass spectrometry proteomics data have been deposited to the ProteomeXchange Consortium via the PRIDE partner repository with the dataset identifier PXD077951. Untargeted metabolomics data were deposited to the MassIVE repository with dataset identifier MSV000101706. There are no restrictions on data availability.

Research involving human participants, their data, or biological material

Policy information about studies with human participants or human data. See also policy information about sex, gender (identity/presentation), and sexual orientation and race, ethnicity and racism.

Reporting on sex and gender	This study does not involve human participants, their data or their biological material
Reporting on race, ethnicity, or other socially relevant groupings	This study does not involve human participants, their data or their biological material
Population characteristics	This study does not involve human participants, their data or their biological material
Recruitment	This study does not involve human participants, their data or their biological material
Ethics oversight	This study does not involve human participants, their data or their biological material

Note that full information on the approval of the study protocol must also be provided in the manuscript.

Field-specific reporting

Please select the one below that is the best fit for your research. If you are not sure, read the appropriate sections before making your selection.

☒ Life sciences ☐ Behavioural & social sciences ☐ Ecological, evolutionary & environmental sciences

For a reference copy of the document with all sections, see nature.com/documents/nr-reporting-summary-flat.pdf

Life sciences study design

All studies must disclose on these points even when the disclosure is negative.

Sample size	No methods to predetermine sample size for experiments were used. Sample sizes were chosen based on robustness of the assay and technical and economical considerations. For cell based assays, a minimum of n=3 biological replicates were conducted. For in vitro enzyme assays, a minimum of 3 independent experiments were performed to ensure reproducibility.
Data exclusions	A minimal number of technical outliers were excluded from in vitro enzyme assay analysis. Outliers were identified conservatively as deviating >50% from the expected value that was corroborated by at least 2 additional independent measurements for the same condition. These outliers likely arise from multichannel pipetting errors over the course of pipetting hundreds of wells per assay, and are not expected to arise from biological varianbility. There were no data exclusions from cell-based assays or any other assay type.
Replication	All results were verified by performing at least two independent biological replicate experiments for cellular assays. All in vitro assays were performed in at least two independent experiments. The precise number of replicates and their nature are reported in figure legends.
Randomization	Not applicable, there was no subjective rating of data involved in our study. Randomization is not applicable for most standard cell culture based assays and in vitro biochemical experiments.
Blinding	Not applicable, there was no subjective rating of data involved in our study.

Reporting for specific materials, systems and methods

We require information from authors about some types of materials, experimental systems and methods used in many studies. Here, indicate whether each material, system or method listed is relevant to your study. If you are not sure if a list item applies to your research, read the appropriate section before selecting a response.

Materials & experimental systems

n/a	Involved in the study
☐	☒ Antibodies
☐	☒ Eukaryotic cell lines
☒	☐ Palaeontology and archaeology
☒	☐ Animals and other organisms
☒	☐ Clinical data
☒	☐ Dual use research of concern
☒	☐ Plants

Methods

n/a	Involved in the study
☒	☐ ChIP-seq
☐	☒ Flow cytometry
☒	☐ MRI-based neuroimaging

Antibodies

Antibodies used	The following antibodies were used in this study for Western blot, with specific dilutions varying depending on the experiment: anti-PPAT (rabbit, ProteinTech, 15401-1-AP); anti-NUDT5 (rabbit, ProteinTech, 27004-1-AP); anti-PYCR2 (rabbit, ProteinTech, 17146-1-AP); anti-HPRT (rabbit, ProteinTech,15059-1-AP); anti-vinculin (rabbit, CST, 4650); anti-FLAG (mouse, Clone M2; Sigma, F1804); anti-DYKDDDDK Tag (rabbit, CST, 1473) anti-NRF2 (rabbit, CST, D1Z9C); anti-NEDD8 (rabbit, CST, 19E3); anti-α-tubulin (mouse, DM1A, Calbiochem, CP06); anti-V5 (rabbit, CST, D3H8Q); anti-HA (rabbit, CST, C29F4)
Validation	All antibodies were used only for Western blot. Anti-PPAT, anti-NUDT5 , and anti-HPRT antibodies were knockout validated in lab. Anti-HA, anti-V5, anti-FLAG (M2) and anti-DYKDDDK antibodies detected exogenously expressed fusion proteins of expected size in cell lysates and did not produce bands in untransfected cells. Anti-PYCR2 (rabbit) was validated through corroborating immunoprecipitation and Western blotting with IP-MS data. Anti-NRF2 antibody is cited in over 800 publications and the Western blot band is stabilized by proteasomal inhibition by MG132. Anti-NEDD8 (19E3) Rabbit mAb detects endogenous levels of both free and conjugated NEDD8 protein. The antibody does not cross-react with other ubiquitin family members, including ubiquitin, SUMO-1, SUMO-2, SUMO-3 and ISG15 (manufacturer website). Anti-tubulin (>750 citations) and anti-vinculin (>350) citations detected bands of expected size.

Eukaryotic cell lines

Policy information about cell lines and Sex and Gender in Research

Cell line source(s)	HEK293T, U2OS, and Expi293F, MOLT4, Jurkat, NALM6 cells were obtained from the University of California Berkeley Cell Culture Facility
Authentication	Human cell line authentication was performed by short tandem repeat analysis using GenePrint 10 System from Promega
Mycoplasma contamination	All cell lines were routinely tested biweekly for mycoplasma contamination using the Mycoplasma PCR Detection Kit (abm, G238) and consistently tested negative.
Commonly misidentified lines (See ICLAC register)	No commonly misidentified cell lines were used in this study.

Plants

Seed stocks	*Report on the source of all seed stocks or other plant material used. If applicable, state the seed stock centre and catalogue number. If plant specimens were collected from the field, describe the collection location, date and sampling procedures.*
Novel plant genotypes	*Describe the methods by which all novel plant genotypes were produced. This includes those generated by transgenic approaches, gene editing, chemical/radiation-based mutagenesis and hybridization. For transgenic lines, describe the transformation method, the number of independent lines analyzed and the generation upon which experiments were performed. For gene-edited lines, describe the editor used, the endogenous sequence targeted for editing, the targeting guide RNA sequence (if applicable) and how the editor was applied.*
Authentication	*Describe any authentication procedures for each seed stock used or novel genotype generated. Describe any experiments used to assess the effect of a mutation and, where applicable, how potential secondary effects (e.g. second site T-DNA insertions, mosiacism, off-target gene editing) were examined.*

Flow Cytometry

Plots

Confirm that:

☒ The axis labels state the marker and fluorochrome used (e.g. CD4-FITC).

☒ The axis scales are clearly visible. Include numbers along axes only for bottom left plot of group (a 'group' is an analysis of identical markers).

☒ All plots are contour plots with outliers or pseudocolor plots.

☒ A numerical value for number of cells or percentage (with statistics) is provided.

Methodology

Sample preparation	Samples were derived from HEK293T or U2OS cell culture experiments in a mixed population of cells expressing either GFP or mCherry markers.
Instrument	BD Biosciences LSR Fortessa Analyzer
Software	FACSDiva (Version 9.0), FlowJo (Version 10.10.0)
Cell population abundance	For all assays, cells populations were determined using fluorescent markers. Non-fluorescent populations were clearly separated from those with fluorescent markers. Sufficient events were ensured for all populations.
Gating strategy	Initial gating steps include identification of live cells (SSC-A/FSC-A) followed by identification of single cells (FSC-H/FSC-A). Then, mCherry+ and GFP+ populations were determined using PE Texas Red-A and FITC-A channels and ratios were calculated.

☒ Tick this box to confirm that a figure exemplifying the gating strategy is provided in the Supplementary Information.
