# Supplementary material for: Metabolite glues as a means of purine sensing and chemotherapeutic response
Source: Nature. 2026 Jul 15;655(8125):1300–8. doi: 10.1038/s41586-026-10790-3 (PMC13421311; doi:10.1038/s41586-026-10790-3)
Supplement: Supplementary file 3 — Peer Review file [file 41586_2026_10790_MOESM3_ESM.pdf]

nature portfolio

Peer Review File

Metabolite glues as a means of purine sensing and chemotherapeutic response

Corresponding Author: Professor Michael Rapé

Any redactions in this file are there to maintain patient confidentiality, the confidentiality of unpublished data, or to remove third-party material.

This file contains all reviewer reports in order by version, followed by all author rebuttals in order by version.

Version 0:

Reviewer comments:

Referee #1

(Remarks to the Author)
This study discusses the role of molecular glues in regulating purine biosynthesis in human cells, specifically focusing on the interaction between the enzyme PPAT and its inhibitor NUDT5. It reveals that purine nucleotides act as molecular glues, enabling cells to monitor and control purine levels, which is crucial for maintaining metabolic balance. The study also highlights how thiopurine chemotherapeutics, used for cancer treatment, function as enhanced molecular glues that inhibit purine synthesis more effectively than natural nucleotides. By exploring the structural flexibility of the binding sites, the research suggests potential pathways for developing new therapeutic agents that could improve treatment efficacy while minimizing side effects.

Major comments:
1. The manuscript suggests that Y74 phosphorylation regulates the R70–Y74 interface, but no in vivo evidence is provided. The authors should show phospho-specific detection (e.g., Western blot or phosphoproteomics) of Y74 modification under physiological or stress conditions. Mutant rescue experiments (WT, Y74F, Y74E) could help confirm its functional relevance.
2. While isotope tracing indicates AMP/NUDT5 feedback inhibition, its physiological impact remains unclear. The authors should test PPAT–NUDT5 interaction or purine flux changes under nutrient fluctuations (e.g., purine depletion and repletion).
3. All experiments use HEK293T or U2OS cells. Including leukemia or lymphoma models where 6-MP and 6-TG are used clinically would greatly strengthen translational significance.
4. The claim that 6-MP acts mainly through the NUDT5–PPAT “glue” mechanism needs direct validation. The authors should use NUDT5 mutants unable to form the complex and test 6-MP/6-TG sensitivity. They should also measure thioguanine incorporation into DNA to discern direct effects of the drug from the glue-mediated effects.
5. The discussion overstates the generality of the “metabolic glue” model. Either tone down the claim or provide supporting evidence (e.g., analysis of metabolite–protein interactions in other pathways).
6. The pocket flexibility observed in the 6-benzylTIMP structure is interesting but raises questions about specificity. Please include a short discussion on selectivity and potential off-target effects to improve clarity for therapeutic relevance.
7. The study mentions AMP-mediated PPAT inhibition independent of NUDT5 but doesn’t quantify it. Comparative kinetic analysis could clarify how this mechanism complements NUDT5-dependent regulation.

Minor comments:
1. Please indicate replicate numbers and statistical tests for all key biochemical assays.
2. Clarify whether enzyme assays were performed with purified PPAT tetramers or mixed oligomeric states.
3. Keep “metabolic glue” for endogenous molecules and “thiopurine molecular glue” for drugs.
4. Add schematic overview panels summarizing the PPAT–NUDT5 glue mechanism and thiopurine interaction to aid readability.
5. Strengthen the connection between purine sensing, disease relevance (e.g., leukemia, immunosuppression), and motivation for discovering new regulatory mechanisms in the introduction.

Referee #2

(Remarks to the Author)
The authors propose a new mode of metabolic regulation in human cells, identifying an endogenous metabolic glue which allows cells to sense and control PPAT mediated purine synthesis through NUDT5 regulation. They link these two proteins via sophisticated exploratory approaches including CRISPR-based genetic screening, protein interactome mapping and elucidate a metabolite-driven feedback loop for nutrient sensing with extensive characterization using high-resolution cryo-EM structural analysis, biochemical reconstitution and enzyme kinetics. In addition to deciphering this previously unknown mechanism the manuscript offers significant broader impact in our understanding the mode of action of Thiopurine drugs which have been in cancer treatment for over 70 years and are shown here to compete with the endogenous metabolic sensing feedback loop. The deep insight into previously unknown mechanism and link to cancer treatment provides exciting elements suitable for publication after the following clarification on mechanistic experiments and some toning down of the claims in the abstract and discussion.
Comments:
1. Many metabolite binding complexes undergo conformational changes exposing molecules to different protein environments and interfaces during reaction cycle cascades or ligand binding events for activity and regulation purposes. Whilst metabolite molecular glue could be a fair description of this new mechanism in purine synthesis I wonder if this really is the first endogenous example as the authors claim. Maybe good to tone this 'first in class' portion of the paper down.
2. There are multiple generalized comments of plasticity of all metabolic glue pockets in the abstract however the authors have only described one example of a metabolic glue. This should be reworded so that it refers to this novel system only.
3. There are some claims about specificity of the system. However, no specificity screens have been performed. The authors nicely show the system can be chemically tuned for improved potency of inhibition but don't provide any data on specificity of these compounds. This should be made clear what is meant by specificity.
4. Molecular glues function through positive cooperativity enhancing protein complex formation between proteins with much weaker affinity for each other. It would be interesting to understand to what extend the binding affinity of the complex is enhanced by this metabolite in the presence and absence of AMP and its analogues. Is this already a stable complex under physiological conditions that simply undergoes conformational changes upon AMP binding in the PPAT site? The titration in extended figure 5h shows very clear binding of NUDT5 in the absence of AMP.
5. Related to the above comment the mechanism in figure 5E suggests oligomeric assembly of PPAT and NUDT5 from dimers to hetero-octomer but there is limited experimental evidence to support this. Does AMP binding really drive oligomerization to form the complex or simply alter the conformation of proteins in the complex? Some light scattering experiments of the complex +- AMP could be interesting. There may also be interesting intermediate effects on oligomerization of the complex by looking at mutations at each interface to help validate the model.
6. The Alpha fold model is described as a heterodimer in apo state, but the complex detected is a larger oligomer bound to AMP. Therefore, alphafold was unable to predict the model because it was not provided with the appropriate information that the authors discovered in this study. With this new information alphafold could at least be given the opportunity to correctly model the full oligomer with and without nucleotides for a fair comparison.
7. The authors explore roles of clinical compounds which are believed to both intercalate into DNA to disrupt replication and inhibit purine synthesis via mimicking the novel feedback mechanism discovered here. Is it possible to chemically de-couple DNA intercalation whilst retaining PPAT inhibition to understand the contributing effects from each pathway in cells?

Referee #3

(Remarks to the Author)
In this manuscript, the authors report that purine nucleotides, represented by AMP, act together with the Nudix family protein NUDT5 to inhibit PPAT, the rate-limiting enzyme in purine biosynthesis. The study presents three novel findings: (1) NUDT5, an ADP-ribose hydrolase previously known to catalyze nuclear ATP production, unexpectedly binds and negatively regulates PPAT independently of its hydrolase activity; (2) NUDT5 inhibits PPAT by inserting its C-terminal tail into the enzyme's active site, together creating an AMP-binding site that is sensitive to AMP at physiological concentrations; (3) the purine nucleotide binding site formed between NUDT5 and PPAT is structurally plastic and can accommodate different thiopurine chemotherapeutics, which elicit their cytotoxic effects partially by inhibiting PPAT and purine biosynthesis.

While three other groups have recently deposited preprints documenting similar findings on the surprising non-enzymatic role of NUDT5 in blocking de novo purine synthesis by inhibiting PPAT (references 50, 51, 52), this manuscript goes beyond those findings by providing mechanistic insights that are otherwise missing from the AlphaFold predictions performed in the other three studies. Based on the characteristic trimolecular interface formed among the NUDT5 C-terminal tail, PPAT, and the purine inhibitor, the authors introduce the concept of "metabolic glues," underscoring how metabolic compounds can regulate enzymes by acting at protein-protein interfaces as molecular glues. This concept expands the chemical space of molecular glues beyond previously documented phytohormones and synthetic compounds, highlighting the broad significance of the molecular glue effect in biology.

This reviewer finds the study exciting and supports its publication after the authors address the following concerns.

Major points

1. One of the major conclusions the authors draw is that AMP stabilizes NUDT5-PPAT interaction as a molecular glue, thereby enhancing the inhibitory function of the Nudix hydrolase. However, the experimental data presented do not fully demonstrate this effect, and the study would benefit from more quantitative measurements to substantiate the proposed

mechanism. The most relevant results are presented in ED Figure 5h, in which in vitro co-IP of recombinant NUDT5 with strep-tagged PPAT was performed in the absence or presence of AMP. While AMP does seem to enhance NUDT5-PPAT binding to a certain degree, the effect is not as pronounced as what the central message of the story suggests. The authors should develop a biophysical method (e.g., SPR or BLI) to measure the affinity of the NUDT5-PPAT interaction with and without the compound. Alternatively, a chemically induced proximity assay (e.g., TR-FRET) could help substantiate the molecular glue concept.

2. Related to the point above, the statement on line 157 is technically imprecise. In Figure 3b (and other PPAT inhibition figures), the AMP dose response curves measured in the presence vs. absence of NUDT5 indicate that NUDT5 increases the potency of AMP by roughly 50-fold, rather than the reverse. Although AMP is expected to potentiate NUDT5 in return due to the cooperativity of the interactions, the data behind the statement do not directly indicate this.

3. Without knowing the Kd value of the NUDT5-PPAT interaction, it is difficult to assess whether the molecular glue effect of AMP mainly impacts NUDT5 binding to PPAT or the engagement of the NUDT5 C-terminal tail at the PPAT active site. If the two proteins show a considerable affinity to each other in the absence of the compound, it might be easier to quantify the molecular glue effect of the compound by measuring the binding between PPAT and an NUDT5 C-terminal peptide.

Minor points

1. ED Figure 5d. Assuming delta-C is delta-210-219, it is surprising that this NUDT5 mutant lacking the C-terminal tail could show some inhibitory effect on PPAT activity (by ~30%) in the absence of AMP. This is related to Figure 3b, in which the delta-C mutant plus AMP shows detectable enhancement in inhibiting PPAT in comparison to AMP only. Does this mean that NUDT5 can inhibit PPAT through another, albeit minor, mechanism, which does not involve blocking the active site of PPAT? Please comment on this in Discussion.

2. The study was built upon a genetic screen with CRL inhibition, which sensitizes the cell to the perturbation of enzymes involved in de novo purine synthesis. However, the story quickly evolves to PPAT inhibition by NUDT5 and AMP. Can the authors comment on how purine biosynthesis is related to CRL functions?

3. Line 161. The sentence needs to be re-worded. Unlike L217, which only contacts AMP, K218 of NUDT5 does make a salt bridge with D398 of PPAT (as shown in Figure 2d and Figure 4f).

4. Line 459 (ED Figure 5d legend). "W26A/W48A" should be "W28A/W46A". The design rationale of this mutant, by the way, is not introduced in either the main text or the ED Figure 5 legend.

5. ED Figure 4e, 4i. Three key PPAT contacting residues in NUDT5, namely Y74, F216, and F219 are not conserved in fish. Does this mean that the PPAT inhibitory function of NUDT5 is lost in fish?

Referee #4

(Remarks to the Author)
I co-reviewed this manuscript with one of the reviewers who provided the listed reports.

Referee #5

(Remarks to the Author)
The study by Witus et al. indicates that endogenous small-molecule glues play an important role in the regulation of metabolism. Given that exogenous small-molecule glues have been known for some time to enhance weak, preexisting protein-protein interactions, it is intuitive that human endogenous small molecules would function in the same manner. Witus et al. convincingly demonstrate that this is indeed the case, uncovering a regulatory mechanism that shows how purine nucleotide levels feed back on and restrict de-novo purine biosynthesis and, moreover, reveals an unexpected aspect of the mode-of action of chemotherapeutics that have been in use for more than half a century.

In addition to presenting a first example for a human endogenous small-molecule glue, this study significantly advances our understanding of how de novo purine biosynthesis and its regulation in response to purine availability. The structural and biochemical studies performed by the authors convincingly demonstrate that purine nucleotides promote the interaction of PPAT with NUDT5 to inhibit PPAT activity and limit de novo purine biosynthesis.

To my knowledge, this is the first report of an endogenous human metabolite family stabilizing a native protein-protein interaction through a molecular glue-like mechanism – there may be other examples for weak stabilization of P-P-interactions by metabolites, but what distinguishes the example presented here is that (i) the endogenous purine nucleotides acting as glues enhance P-P-interaction by almost two orders of magnitude, and (ii) that affinity of the pocket the purine nucleotides bind to when part of the P-P complex is compatible with the cellular concentrations of purine nucleotides, demonstrating biological relevance.

Taken together, the findings reported in this manuscript are of high significance for three reasons, because they (i) expand our understanding of how endogenous metabolites can regulate protein function in humans, (ii) demonstrate a feedback

mechanism that regulates purine biosynthesis, and (iii) provides strong evidence for an unexpected aspect of the mode of action for widely used chemotherapeutics.

However, I feel that the authors should be cautious not to overinterpret their data by extrapolating too much – I agree that it is likely that metabolic glues (I'm not particularly fond of this term, how about "metabolite glues") play a role in the regulation of many other metabolic and signaling pathways in diverse organisms. But I do not think there is enough data to predict (as the authors do, e.g., in line 319) that a high degree of structural flexibility is a general feature of metabolic glue pockets, as opposed to glue pockets of non-endogenous compounds. The current study simply does not provide sufficient evidence to make this claim, as it describes essentially one single example. It could certainly be that endogenous small-molecule glues bind to generally more flexible pockets than non-endogenous glues, but this can't be concluded based on the data presented here. Rather, it appears that the relative flexibility of this particular pocket is adapted to the diversity of purine metabolites present in the cell, and takes advantage of the fact that metabolites from this pathway - consisting of a polar, acidic phosphorylated ribose attached to a less polar, aromatic moiety - exhibit some general structural commonalities. Other pathways may have evolved to sense one specific metabolite, representing a key intermediate with characteristic (and perhaps otherwise uncommon) structural features, and correspondingly, the relevant binding pocket may exhibit much less flexibility, more similar to known non-endogenous glues.

Minor comments:
- I'm surprised the authors did not actually attempt to demonstrate that the R70-Y74 interface is likely broken by phosphorylation of Y74. It would seem that the team has all the necessary tools in hand. This is not critical, but would round the narrative by providing insight in how regulation by metabolite glues intersects with phosphorylation-based cascades to regulate purine biosynthesis.

- In the last part of the Results section, it seems unnecessary to first discuss 6-benzyl and then 6-ethyl-TIMP, in two separate paragraphs. This section should be condensed.

- The legends to Figures 1b-e lack necessary detail. For example, the color coding in 1b is not explained – one can infer indirectly (looking at panel 1d) that the colors relate to the role of the proteins in purine or pyrimidine biosynthesis, but this should be explicitly stated in the legend (the insert in the lower right of 1b is not a substitute, as is this merely adds to the confusion). Also, the description of the genetic screen in the main text is too abbreviated – it should be possible for a non-expert to understand what was done, without having to resort to references or the method section.

line 301: "prevent excessive use of metabolic building blocks." If I understand the authors' intent correctly, the point is that de novo purine biosynthesis, i.e., misallocation of metabolic resources, is prevented.

line 49: "amino acids, or ribose-5-phosphate" should be "amino acids, and ribose-5-phosphate"

line 100: extraneous "how".

Figure 3c and elsewhere – p-values should be added where appropriate.

Figure 4a: It should be indicated that the PPAT activity assay panels compare benzyl- (top) and ethyl- (bottom) derivatives with 6-meTIMP. Also, please confirm whether the same data for 6-meTIMP are shown in the top and bottom panels.

Extended Data Figure 2: If this is from modeling the monomer interaction, this should be covered in the Methods (the current AlphaFold section describes modeling of the 4-8 complex).

Methods:
Line 879: when calculating M+2, was the contribution from the 2x13C peak removed?
Line 874: "area … were"" singular-plural mismatch.
Line 888: "2uL" – should be microliter

Version 1:

Reviewer comments:

Referee #1

(Remarks to the Author)
The authors have addressed all my concerns. The manuscript is now acceptable for publication.

Referee #2

(Remarks to the Author)
The authors have responded constructively to all concerns, with appropriate revisions, experiments, and clearer framing of claims.
Specifically reduced overstatements of specificity to clearer wording and softened claims of first in class while preserving the novelty of this endogenous metabolite glue mechanism. Biochemical assays significantly clarify the mode of action

demonstrating the metabolite glue stabilizes rather than initiates complex formation. New cellular data disentangle, to a reasonable extent, the contribution of the glue mechanism from other effects such as DNA intercalation, supporting the biological and clinical significance.

Referee #3

(Remarks to the Author)
In this revised manuscript, the authors have added new mass photometry and AlphaLisa data quantifying the effects of AMP on complex formation between PPAT and NUDT5 and on preventing their complex disassembly induced by the PRPP substrate. These new data adequately address the major concerns from this reviewer and significantly strengthen the general conclusion of the study. This reviewer recommends the publication of this story.

Referee #4

(Remarks to the Author)
I co-reviewed this manuscript with one of the reviewers who provided the listed reports.

Referee #5

(Remarks to the Author)
In their revision the authors added substantial new data demonstrating assembly of the PPAT-NUDT5 complex in vitro and in cells under different nutrient conditions, including nutrient-dependent regulation of complex formation in cells. Further, the authors now include mass spectrometric data that confirm phosphorylation at Y74 of NUDT5 [new Fig. 1i]. These new results further support the authors' model for feedback regulation of purine biosynthesis and demonstrate its physiological relevance.
Myself and several other reviewers had taken issue with the authors' conjecture that a high degree of structural flexibility is a general feature of metabolic glue pockets. In the revised discussion, the authors have toned down their claims somewhat, but the wording could still be somewhat more careful (please see suggested edits below).

There is one additional point that I missed in my initial review and that, however, I think is important to address: Figure 1b shows that sgRNAs for enzymes that divert IMP from AMP production to GMP production, especially IMPDH2, are protective, as opposed to enzymes in the mainstem purine biosynthetic pathway. In fact, IMPDH2 is more protective than NUDT5. How does that fit with the model presented here? Is there any evidence that the AMP-to-GMP ratio feeds back in some way on purine biosynthesis, e.g., via PPAT? If the role of IMPDH2 cannot explained at this stage, it would be essential to comment on this in the Discussion, as an observation that the current model cannot yet explain.

Minor points:
- the last two paragraphs of the Results section are still poorly integrated. The importance of the large hydrophobic pocket for accommodating 6-benzylTIMP is explicitly discussed in lines 293-302, but then the following paragraph again starts by stating that there is a hydrophobic pocket. This last paragraph should be condensed significantly.
- Line 322 "Metabolite glues restrict de novo purine..". Not all metabolite glues will do this. What is meant is that "The identified metabolite glues restrict de novo purine…".
- Line 325: "endogenous molecular glues serve crucial regulatory functions". I think "can serve" would be more appropriate.

Version 1 continued [informal]:

Reviewer comments:

Referee #5
(Remarks to the Author)

In their revision the authors added substantial new data demonstrating assembly of the PPAT-NUDT5 complex in vitro and in cells under different nutrient conditions, including nutrient-dependent regulation of complex formation in cells. Further, the authors now include mass spectrometric data that confirm phosphorylation at Y74 of NUDT5 [new Fig. 1i]. These new results further support the authors' model for feedback regulation of purine biosynthesis and demonstrate its physiological relevance.
Myself and several other reviewers had taken issue with the authors' conjecture that a high degree of structural flexibility is a general feature of metabolic glue pockets. In the revised discussion, the authors have toned down their claims somewhat, but the wording could still be somewhat more careful (please see suggested edits below).

There is one additional point that I missed in my initial review and that, however, I think is important to address: Figure 1b shows that sgRNAs for enzymes that divert IMP from AMP production to GMP production, especially IMPDH2, are protective, as opposed to enzymes in the mainstem purine biosynthetic pathway. In fact, IMPDH2 is more protective than NUDT5. How does that fit with the model presented here? Is there any evidence that the AMP-to-GMP ratio feeds back in some way on purine biosynthesis, e.g., via PPAT? If the role of IMPDH2 cannot explained at this stage, it would be essential to comment on this in the Discussion, as an observation that the current model cannot yet explain.

Minor points:
- the last two paragraphs of the Results section are still poorly integrated. The importance of the large hydrophobic pocket for accommodating 6-benzylTIMP is explicitly discussed in lines 293-302, but then the following paragraph again starts by stating that there is a hydrophobic pocket. This last paragraph should be condensed significantly.
- Line 322 "Metabolite glues restrict de novo purine..". Not all metabolite glues will do this. What is meant is that "The identified metabolite glues restrict de novo purine...".
- Line 325: "endogenous molecular glues serve crucial regulatory functions". I think "can serve" would be more appropriate.

Open Access This Peer Review File is licensed under a Creative Commons Attribution 4.0 International License, which permits use, sharing, adaptation, distribution and reproduction in any medium or format, as long as you give appropriate credit to the original author(s) and the source, provide a link to the Creative Commons license, and indicate if changes were made.
In cases where reviewers are anonymous, credit should be given to 'Anonymous Referee' and the source.
The images or other third party material in this Peer Review File are included in the article's Creative Commons license, unless indicated otherwise in a credit line to the material. If material is not included in the article's Creative Commons license and your intended use is not permitted by statutory regulation or exceeds the permitted use, you will need to obtain permission directly from the copyright holder.
To view a copy of this license, visit https://creativecommons.org/licenses/by/4.0/

Response to Referees' Comments

We thank all reviewers for their expertise, time, and insightful feedback. We have been very much encouraged by the enthusiasm for our manuscript. In this revised version, we have addressed all comments by incorporating new experimental data or refining the text. These new results have substantially improved our understanding of the biochemical mechanisms and signaling functions of metabolite glues – which to our knowledge, are the first molecular glues found to act at an endogenous level in human cells. We thank all reviewers for motivating us to perform these experiments.

We now show that metabolite glues protect inhibitory PPAT-NUDT5 complexes against substrate-induced dissociation, a function that is true to the roles of glues even beyond biology. We also provide more evidence using clinically relevant ALL cell lines that the glue mechanism discovered here provides mechanistic insight into a chemotherapeutic that has been used to treat ALL for almost 70 years. Thus, this study reveals exciting biology with important implications for drug discovery and development.

Referee 1:

This study discusses the role of molecular glues in regulating purine biosynthesis in human cells, specifically focusing on the interaction between the enzyme PPAT and its inhibitor NUDT5. It reveals that purine nucleotides act as molecular glues, enabling cells to monitor and control purine levels, which is crucial for maintaining metabolic balance. The study also highlights how thiopurine chemotherapeutics, used for cancer treatment, function as enhanced molecular glues that inhibit purine synthesis more effectively than natural nucleotides. By exploring the structural flexibility of the binding sites, the research suggests potential pathways for developing new therapeutic agents that could improve treatment efficacy while minimizing side effects.

We thank this reviewer for the many insightful comments on our manuscript, which we believe strongly improved this study.

Major comments:

1. The manuscript suggests that Y74 phosphorylation regulates the R70–Y74 interface, but no in vivo evidence is provided. The authors should show phospho-specific detection (e.g., Western blot or phosphoproteomics) of Y74 modification under physiological or stress conditions. Mutant rescue experiments (WT, Y74F, Y74E) could help confirm its functional relevance.

While phosphorylation of NUDT5 Y74 (p-Y74) had previously been reported in the PhosSitePlus database (**Fig. 1g**, link to database), we now used DIA mass spectrometry to directly show phosphorylation of Y74 of NUDT5 **[new Fig. 1i]**. Loss of phosphorylation in NUDT5^{Y74F} documents the specificity of our approach, with the small amount of remaining signal likely resulting from co-immunoprecipitated WT NUDT5. Most importantly, we do not detect any phosphorylation of NUDT5-Y74, when NUDT5 is bound to PPAT. While discovering upstream signaling pathways that result in Y74 phosphorylation would be beyond the scope of this manuscript, these results deepen our mechanistic insight by showing that modification of Y74 in NUDT5 is incompatible with formation of a stable PPAT-NUDT5 complex.

2. While isotope tracing indicates AMP/NUDT5 feedback inhibition, its physiological impact remains unclear. The authors should test PPAT–NUDT5 interaction or purine flux changes under nutrient fluctuations (e.g., purine and repletion).

To complement our isotope tracing and *in vitro* PPAT activity assays, we have now rigorously characterized the formation and disassembly of the PPAT-NUDT5 complex *in vitro* and in cells under different nutrient conditions. Using quantitative AlphaLisa binding assays and mass photometry, we now show that the PPAT-NUDT5 complex dissociates in the presence of the PPAT substrate PRPP, which is counteracted by AMP or other purine glues **[new Fig 3c, d; new Fig. 4g; new Extended Data Fig. 6b; new Extended Data fig. 8e]**. In cells, we had already noted that the PPAT-NUDT5 complex dissociates upon treatment with antifolates (methotrexate and lometrexol) that deplete purines and elevate PRPP levels **[Fig. 4h; Extended Data Fig. 6f, g]**. We now show that antifolate-induced dissociation of PPAT-NUDT5 is reversed by supplementing cells with hypoxanthine, which repletes nucleotide pools while consuming PRPP **[new Fig. 3e],** directly demonstrating nutrient-dependent regulation of complex formation in cells. Together, our data support a model in which the PPAT-NUDT5 complex disassembles to activate PPAT under conditions favoring *de novo* purine synthesis (low AMP/GMP and high PRPP substrate availability).

3. All experiments use HEK293T or U2OS cells. Including leukemia or lymphoma models where 6-MP and 6-TG are used clinically would greatly strengthen translational significance.

We thank this reviewer for this important suggestion. We have now generated Δ*NUDT5* and *NUDT5*$^{L217A/K218A}$ mutant MOLT4 (T-ALL), Jurkat (T-ALL) and NALM6 (B-ALL) cell lines and monitored cytotoxicity of the mercaptopurine metabolite 6-meTIMP **[new Fig. 4k]**. We observed similar cytotoxicity profiles in these ALL cells as we had previously noted in HEK293T, including an important role for an intact glue interface. These findings underscore the clinical relevance of our discoveries.

4. The claim that 6-MP acts mainly through the NUDT5–PPAT "glue" mechanism needs direct validation. The authors should use NUDT5 mutants unable to form the complex and test 6-MP/6-TG sensitivity. They should also measure thioguanine incorporation into DNA to discern direct effects of the drug from the glue-mediated effects.

Our original data had strongly supported the notion that gluing NUDT5 to PPAT is a critical function of this cancer therapeutic. We had shown in competition assays that cells deficient in formation of the PPAT-NUDT5 complex ($NUDT5^{3A}$ cells) or unable to support the glue interaction ($NUDT5^{L217A/K218A}$ cells) are highly resistant to thiopurine treatment with 6-MP and 6-TG **[Extended Data Fig. 8a; Fig. 4j]**. These results had highlighted the importance of the glue mechanism for restraining cancer cell line proliferation upon 6-MP treatment. However, this reviewer correctly points out that this did not exclude the possibility of other cytotoxic effects of this drug. Indeed, 6-MP and 6-TG are known to be highly toxic also in healthy tissues, an issue that has limited the therapeutic window and efficacy of these long-used compounds.

To better delineate the cytotoxic effects of thiopurines, we treated multiple ALL cell lines with the 6-MP metabolite 6-meTIMP, which is the most potent PPAT-NUDT5 glue *in vitro*. Importantly, we found that 6-meTIMP shows much reduced cytotoxic effects when the glue pocket has been disrupted in $\mathit{NUDT5}^{L217A/K218A}$ cells **[new Fig. 4k]**. The resistance of glue-deficient ALL cells to 6-meTIMP is very similar to that observed in Δ*NUDT5* cells that entirely lack this protein inhibitor of PPAT, and these effects are observed within the range of 6-meTIMP concentrations that are experienced in patients during 6-MP treatment. At higher 6-meTIMP concentrations, we observed cytotoxicity independent of NUDT5, potentially through interference with other nucleotide-binding proteins or due to non-specific off-target effects. Using clinically relevant cell lines, these data therefore strongly support the notion that the glue mechanism strongly contributes to 6-MP sensitivity via the thiopurine glue metabolite 6-meTIMP, which is a potent blocker of de novo purine synthesis.

5. The discussion overstates the generality of the "metabolic glue" model. Either tone down the claim or provide supporting evidence (e.g., analysis of metabolite–protein interactions in other pathways).

While finalizing this work, we discovered a second metabolite glue that regulates heme biosynthesis, indicating that the findings reported here will be of general importance. Of course, we realize that making such statements would require us to publish the second glue. We have accordingly updated the text to specify that observations described in the current manuscript reflect the PPAT-NUDT5 complex. while suggesting that more general features of metabolite glue signaling will be borne out with additional examples.

6. The pocket flexibility observed in the 6-benzylTIMP structure is interesting but raises questions about specificity. Please include a short discussion on selectivity and potential off-target effects to improve clarity for therapeutic relevance.

Following the advice of this reviewer, we both updated our discussion and added new experiments to address this issue. Using ALL cell lines engineered to probe the contribution of the glue mechanism to cytotoxic effects of 6-MP, we have observed both glue-dependent and independent effects of the 6-MP thiopurine glue 6-meTIMP. The glue-independent effects, seen at high concentrations when 6-meTIMP kills Δ*NUDT5* and *NUDT5*$^{L217A/K218A}$ cells, motivated the discovery of glues that are more specific for PPAT-NUDT5 **[new Fig. 4k]**.

We hypothesized that addition of bulky side groups may increase specificity through weakening interactions with other proteins that do not possess similar flexibility in their nucleotide-binding pockets. Although this would be challenging to test for 6-benzylTIMP that likely does not cross the cell membrane and is recognized less efficiently by nucleotide processing enzymes, we were able to test this hypothesis for 6-ethylTIMP. Strikingly, although 6-ethylTIMP was slightly less cytotoxic than 6-meTIMP across all cell lines, growth inhibition was more dependent on both NUDT5 as well as the molecular glue mechanism than 6-meTIMP **[new Fig. 5e]**. These experiments not only document the specificity of the glue pocket, but also suggest that further optimization of glue derivatives can yield more specific PPAT-NUDT5 inhibitors that do not target other proteins and hence possess a broader therapeutic window.

7. The study mentions AMP-mediated PPAT inhibition independent of NUDT5 but doesn't quantify it. Comparative kinetic analysis could clarify how this mechanism complements NUDT5-dependent regulation.

We apologize if our description of our results had been lacking. We had actually quantified AMP-mediated PPAT inhibition independent of NUDT5 in **Fig. 3b** (gray curve). Under these conditions, ~13mM AMP is required to inhibit PPAT, which is far outside of the physiological range for monophosphorylated nucleotides. We have reworded our text to clarify this point, as was also requested by another referee.

Minor comments:

1. Please indicate replicate numbers and statistical tests for all key biochemical assays.

We have updated the figure legends to include this information and included statistical tests where warranted.

2. Clarify whether enzyme assays were performed with purified PPAT tetramers or mixed oligomeric states.

Purified PPAT dimers, as now observed by mass-photometry **[new Extended Data Fig. 6c]**, were the initial reaction component in the enzyme assays. However, we note that PPAT oligomeric state changes in the

presence of metabolites and NUDT5. This detail is now included in the methods sections and shown in **new Fig 3d and new Extended Data Fig. 6d, e**.

3. Keep "metabolic glue" for endogenous molecules and "thiopurine molecular glue" for drugs.

Together with the request by another reviewer to rename glues 'metabolite glues', we have done our best to implement the nomenclature suggested by this reviewer across the manuscript. We note that sometimes when referring to thiopurine activity we frame its activity in the context of the metabolite glue binding pocket, as this refers to the physical glue binding site in the protein that is the same for endogenous and therapeutic molecules.

4. Add schematic overview panels summarizing the PPAT–NUDT5 glue mechanism and thiopurine interaction to aid readability.

We have now added an additional panel summarizing the mechanism **[new Fig. 3h]**, where we present data revealing the nutrient-driven dynamics of the glue interaction. We have also kept an abridged version of the model panel in **Fig. 5f**.

5. Strengthen the connection between purine sensing, disease relevance (e.g., leukemia, immunosuppression), and motivation for discovering new regulatory mechanisms in the introduction.

We have updated the introduction to strengthen these points. On a basic level, we stipulate that little is known about regulation of purine biosynthetic enzymes in metazoans, motivating the study of metazoan-specific mechanisms (end of paragraph 2). We then propose that identifying new regulatory mechanisms of purine sensing that control purine production may reveal new strategies to modulate these processes in cancer and autoimmunity—an approach that has not been fully realized in the clinic (end of paragraph 3).

Referee 2:

The authors propose a new mode of metabolic regulation in human cells, identifying an endogenous metabolic glue which allows cells to sense and control PPAT mediated purine synthesis through NUDT5 regulation. They link these two proteins via sophisticated exploratory approaches including CRISPR-based genetic screening, protein interactome mapping and elucidate a metabolite-driven feedback loop for nutrient sensing with extensive characterization using high-resolution cryo-EM structural analysis, biochemical reconstitution and enzyme kinetics. In addition to deciphering this previously unknown mechanism the manuscript offers significant broader impact in our understanding the mode of action of

Thiopurine drugs which have been in cancer treatment for over 70 years and are shown here to compete with the endogenous metabolic sensing feedback loop. The deep insight into previously unknown mechanism and link to cancer treatment provides exciting elements suitable for publication after the following clarification on mechanistic experiments and some toning down of the claims in the abstract and discussion.

We thank this reviewer for their very generous comments on our manuscript. We have added several new experiments that further improved our study, providing even deeper insight into the biochemical function and signaling role of metabolite glues.

Comments:

1. Many metabolite binding complexes undergo conformational changes exposing molecules to different protein environments and interfaces during reaction cycle cascades or ligand binding events for activity and regulation purposes. Whilst metabolite molecular glue could be a fair description of this new mechanism in purine synthesis I wonder if this really is the first endogenous example as the authors claim. Maybe good to tone this 'first in class' portion of the paper down.

We appreciate this reviewer's comment. While we believe that this is the first example of an endogenous molecular glue in human cells, it is difficult to exclude with absolute certainty that other previously described metabolites behave as *bona fide* molecular glues. As such, we have followed this reviewer's advice and softened the wording of this claim while still highlighting its novelty.

2. There are multiple generalized comments of plasticity of all metabolic glue pockets in the abstract however the authors have only described one example of a metabolic glue. This should be reworded so that it refers to this novel system only.

We have reworded our discussion to be more specific to this example of the PPAT-NUDT5 complex. We propose in the discussion that this may be a general feature of metabolite glue complexes involving metabolic enzymes to be borne out with additional examples.

3. There are some claims about specificity of the system. However, no specificity screens have been performed. The authors nicely show the system can be chemically tuned for improved potency of inhibition but don't provide any data on specificity of these compounds. This should be made clear what is meant by specificity.

To provide a test of specificity for improved therapeutic glues, we used 6-ethylTIMP that *in vitro* showed increased potency as a molecular glue compared to 6-meTIMP, the major metabolite of 6-MP. In cells, 6-ethylTIMP was slightly less cytotoxic than 6-meTIMP across three different ALL cell lines, potentially due to decreased cellular uptake or more rapid turnover. Importantly, however, 6-ethylTIMP was also more specific, as its impact on cell viability was more dependent on NUDT5 and the molecular glue mechanism **[new Fig. 5e]**. Thus, it is possible that thiopurine optimization based on our structural insight may leverage the flexibility of the PPAT-NUDT5 glue pocket to yield more specific PPAT-NUDT5 inhibitors that broaden the therapeutic window of these widely used cancer drugs.

4. Molecular glues function through positive cooperativity enhancing protein complex formation between proteins with much weaker affinity for each other. It would be interesting to understand to what extend the binding affinity of the complex is enhanced by this metabolite in the presence and absence of AMP and its analogues. Is this already a stable complex under physiological conditions that simply undergoes conformational changes upon AMP binding in the PPAT site? The titration in extended figure 5h shows very clear binding of NUDT5 in the absence of AMP.

We thank this reviewer for raising this issue, which motivated us to perform a series of experiments that greatly deepen our insight into the biochemical mechanism of metabolite glue action. We have developed *in vitro* binding assays based on AlphaLisa or mass photometry to monitor PPAT-NUDT5 complex formation and stability. We found that while PPAT and NUDT5 can assemble into the full hetero-octameric complex in the absence of AMP **[new Fig. 3d; new Extended Data Fig. 6a]**, AMP promoted complex formation. Mutating the metabolite glue interface (L217A/K218A) had a significant impact on AMP-stimulated complex formation, indicative of a role of metabolite glues in complex formation. However, mutation of the glue interface had less dramatic effects than what we observed for the $NUDT5^{3A}$ mutant that completely lost binding **[new Fig. 2e]**, indicating that the purine glues might function at an additional step following initial complex formation.

Given the potency of metabolite glues in PPAT inhibition, we hypothesized that the function of glues may also be tied to the mechanism of PPAT activation by its substrate PRPP. In line with this, we now found that PRPP induces dissociation of the PPAT-NUDT5 complex **[new Fig. 3c, d]**. Strikingly, substrate-induced complex dissociation was blocked if AMP or thiopurine glues were present, and this was fully dependent on an intact glue interface **[new Fig. 3c, d; new Fig 4g; new Extended Data Fig. 6b; new Extended Data Fig. 8e]**. Our work thus shows that the glue mechanism prevents premature complex disassembly, thereby counteracting PPAT activation in the presence of either low substrate (PRPP) or abundant product (AMP). It is interesting to note that this glue function resembles glues in real life: preserving adherence of components (such as sheets of paper), rather than inducing their initial assembly.

5. Related to the above comment the mechanism in figure 5E suggests oligomeric assembly of PPAT and NUDT5 from dimers to hetero-octomer but there is limited experimental evidence to support this. Does AMP binding really drive oligomerization to form the complex or simply alter the conformation of proteins in the complex? Some light scattering experiments of the complex +- AMP could be interesting. There may also be interesting intermediate effects on oligomerization of the complex by looking at mutations at each interface to help validate the model.

We have now monitored complex formation by mass photometry, which reports on the mixture of oligomeric states in solution **[new Fig. 3d; new Extended Data Fig. 6c-e]**. While we found that the PPAT-NUDT5 complex can form in the absence of AMP, AMP does promote complex formation at relevant protein concentrations in cells (~1 μM; values obtained from OpenCell database, link). As mentioned above, these data complement the critical function of AMP in preventing substrate-induced complex dissociation. Importantly, we observed that complex formation using glue-deficient mutant NUDT5$^{L217A/K218A}$ is not stimulated by AMP, and the complex is not protected from PRPP-induced dissociation **[new Fig. 3d]**. Together, these data highlight the crucial functions of the metabolite glue in promoting complex formation and stabilization.

6. The Alpha fold model is described as a heterodimer in apo state, but the complex detected is a larger oligomer bound to AMP. Therefore, alphafold was unable to predict the model because it was not provided with the appropriate information that the authors discovered in this study. With this new information alphafold could at least be given the opportunity to correctly model the full oligomer with and without nucleotides for a fair compareson.

Following the suggestion of this reviewer, we have now folded the full complex (4x PPAT and 4x NUDT5) with 8 molecules of AMP, information that was only available after we solved the cryo-EM structure. AlphaFold3 does predict the interaction between the C-terminal tail of NUDT5 with AMP bound at the molecular glue interface in a manner that is partially correct. We have included this data as **new Extended Data Fig. 2c, d**.

7. The authors explore roles of clinical compounds which are believed to both intercalate into DNA to disrupt replication and inhibit purine synthesis via mimicking the novel feedback mechanism discovered here. Is it possible to chemically de-couple DNA intercalation whilst retaining PPAT inhibition to understand the contributing effects from each pathway in cells?

We thank this reviewer for this important comment. We have now performed experiments to better delineate the cytotoxic effects of thiopurines, using engineered cell lines that represent the cancer, ALL, that is still treated with mercaptopurines. We treated these ALL cells with the 6-MP metabolite 6-meTIMP, which we have found to be the most potent PPAT-NUDT5 glue *in vitro*. Importantly, we found that 6-meTIMP shows cytotoxic effects in *NUDT5*$^{L217A/K218A}$ cells only at much higher concentrations than in WT cells **[new Fig. 4k]**. The resistance of glue-deficient ALL cells to 6-meTIMP is very similar to that observed in Δ*NUDT5* cells that entirely lack this PPAT

inhibitor. The differences in cytotoxic behavior of 6-meTIMP in WT compared to glue-deficient ALL cells occur at 6-meTIMP concentrations that are within the range of those experienced in patients during 6-MP treatment. At higher 6-meTIMP concentrations, we observed cytotoxicity independent on NUDT5, potentially through interference with other nucleotide-binding proteins and off-target effects. Using clinically relevant cell lines, these data therefore strongly support the notion that the glue mechanism strongly contributes to 6-MP sensitivity via the thiopurine glue metabolite 6-meTIMP, which is a potent blocker of de novo purine synthesis.

Referee 3:

In this manuscript, the authors report that purine nucleotides, represented by AMP, act together with the Nudix family protein NUDT5 to inhibit PPAT, the rate-limiting enzyme in purine biosynthesis. The study presents three novel findings: (1) NUDT5, an ADP-ribose hydrolase previously known to catalyze nuclear ATP production, unexpectedly binds and negatively regulates PPAT independently of its hydrolase activity; (2) NUDT5 inhibits PPAT by inserting its C-terminal tail into the enzyme's active site, together creating an AMP-binding site that is sensitive to AMP at physiological concentrations; (3) the purine nucleotide binding site formed between NUDT5 and PPAT is structurally plastic and can accommodate different thiopurine chemotherapeutics, which elicit their cytotoxic effects partially by inhibiting PPAT and purine biosynthesis.

While three other groups have recently deposited preprints documenting similar findings on the surprising non-enzymatic role of NUDT5 in blocking de novo purine synthesis by inhibiting PPAT (references 50, 51, 52), this manuscript goes beyond those findings by providing mechanistic insights that are otherwise missing from the AlphaFold predictions performed in the other three studies. Based on the characteristic trimolecular interface formed among the NUDT5 C-terminal tail, PPAT, and the purine inhibitor, the authors introduce the concept of "metabolic glues," underscoring how metabolic compounds can regulate enzymes by acting at protein-protein interfaces as molecular glues. This concept expands the chemical space of molecular glues beyond previously documented phytohormones and synthetic compounds, highlighting the broad significance of the molecular glue effect in biology.

This reviewer finds the study exciting and supports its publication after the authors address the following concerns.

We are deeply grateful for these very generous comments, and we have carefully addressed all issues raised by this reviewer. Thank you!

Major points

1. One of the major conclusions the authors draw is that AMP stabilizes NUDT5-PPAT interaction as a molecular glue, thereby enhancing the inhibitory function of the Nudix hydrolase. However, the experimental data presented do not fully demonstrate this effect, and the study would benefit from more quantitative measurements to substantiate the proposed mechanism. The most relevant results are presented in ED Figure 5h, in which in vitro co-IP of recombinant NUDT5 with strep-tagged PPAT was performed in the absence or presence of AMP. While AMP does seem to enhance NUDT5-PPAT binding to a certain degree, the effect is not as pronounced as what the central message of the story suggests. The authors should develop a biophysical method (e.g., SPR or BLI) to measure the affinity of the NUDT5-PPAT interaction with and without the compound. Alternatively, a chemically induced proximity assay (e.g., TR-FRET) could help substantiate the molecular glue concept.

We agree with this reviewer's assessment and accordingly performed quantitative *in vitro* binding assays based on AlphaLisa to monitor PPAT-NUDT5 complex formation and stability. We have also directly measured complex formation and dynamics using mass photometry. We found that PPAT and NUDT5 can assemble into the full hetero-octameric complex in the absence of AMP, but complex formation is stimulated by the metabolite glue **[new Fig. 3d; new Extended Data Fig. 6a]**. Importantly, stimulation of complex formation by AMP was observed at PPAT and NUDT5 concentrations reflective of those measured in cells (~1 uM; values obtained from OpenCell database, link). Mutating the glue interface (L217/K218A) impaired complex formation, but as noted by this reviewer, this effect was less dramatic than what we observed for the $NUDT5^{3A}$ mutant that completely lost binding **[new Fig. 2e]**.

Given the importance of the glue mechanism for catalytic inhibition of PPAT, we hypothesized that purine glues may regulate PPAT activation in response to increase PRPP substrate availability. In line with this notion, we found by both AlphaLisa and mass photometry that PRPP induces dissociation of the PPAT-NUDT5 complex **[new Fig. 3c, d]**. Strikingly, PRPP-dependent complex dissociation was strongly blocked if AMP or thiopurine glues were present, and this role was fully dependent on an intact glue interface **[new Fig. 3c, d; new Fig. 4g; new Extended Data Fig. 6b; new Extended Data Fig. 8e]**. Our work now clearly shows that the glue mechanism both promotes complex formation and prevents PRPP-induced disassembly, thereby counteracting premature PPAT activation in the presence of low substrate (PRPP) or high product (AMP) levels. It is interesting to note that this function resembles the best-known role of glues in real life: preserving adherence of components (such as sheets of paper), rather than inducing their initial assembly. We thank this reviewer for motivating these critical experiments.

2. Related to the point above, the statement on line 157 is technically imprecise. In Figure 3b (and other PPAT inhibition figures), the AMP dose response curves measured in the presence vs. absence of NUDT5 indicate that NUDT5 increases the potency of AMP by roughly 50-fold, rather than the reverse. Although AMP is expected to potentiate NUDT5 in return due to the cooperativity of the interactions, the data behind the statement do not directly indicate this.

Thank you for pointing this out, and we have changed the text accordingly.

3. Without knowing the Kd value of the NUDT5-PPAT interaction, it is difficult to assess whether the molecular glue effect of AMP mainly impacts NUDT5 binding to PPAT or the engagement of the NUDT5 C-terminal tail at the PPAT active site. If the two proteins show a considerable affinity to each other in the absence of the compound, it might be easier to quantify the molecular glue effect of the compound by measuring the binding between PPAT and an NUDT5 C-terminal peptide

We have now quantified the K_d for the PPAT-NUDT5 interaction for wild-type and mutant proteins. We observe that mutating the C-terminal tail of NUDT5 has a significant effect on complex affinity (~7-fold compared to WT; **new Fig. 2e**). Using an AlphaLisa binding assay and mass photometry, we show that an additional important function of the AMP glue is to prevent premature dissociation of the PPAT-NUDT5 complex by the substrate, PRPP **[new Fig. 3c, d]**. When the glue interaction is lost in the $NUDT5^{L217A/K218A}$ mutant, AMP does not provide any buffering to PRPP-induced disassembly of the complex and fails to enhance AMP-mediated inhibition of PPAT.

Minor points

1. ED Figure 5d. Assuming delta-C is delta-210-219, it is surprising that this NUDT5 mutant lacking the C-terminal tail could show some inhibitory effect on PPAT activity (by ~30%) in the absence of AMP. This is related to Figure 3b, in which the delta-C mutant plus AMP shows detectable enhancement in inhibiting PPAT in comparison to AMP only. Does this mean that NUDT5 can inhibit PPAT through another, albeit minor, mechanism, which does not involve blocking the active site of PPAT? Please comment on this in Discussion.

Our data suggest that NUDT5 can provide some AMP-independent inhibition of PPAT **[Extended Data Fig. 5d]**. This stems from its ability to bind to PPAT via the R70-Y74 interface, likely rigidifying PPAT in a manner that is incompatible with full enzyme activity. We have now commented on this in the discussion. We note that the difference between the delta-C and L217A/K218A mutants and no NUDT5 in **Fig. 3b** is not statistically significant, which is now explicitly shown in the figure.

2. The study was built upon a genetic screen with CRL inhibition, which sensitizes the cell to the perturbation of enzymes involved in de novo purine synthesis. However, the story quickly evolves to PPAT inhibition by NUDT5 and AMP. Can the authors comment on how purine biosynthesis is related to CRL functions?

We currently do not know how CRL function is related to defects in purine metabolism and we are performing unbiased genetic screens to identify CRL complexes that may mediate these effects. In addition, we note that some roles of CRLs in regulating purine flux via purinosome assembly (Chou et al., Mol Cell. 2023) and the nucleobase transporter ENT2 (Emanuele et al., Cell. 2011.) have been reported. CRLs also are implicated in many other metabolic processes that feed into purine synthesis, such as regulation of mitochondrial health and activity. We now briefly comment on this interesting issue in our revised manuscript.

3. Line 161. The sentence needs to be re-worded. Unlike L217, which only contacts AMP, K218 of NUDT5 does make a salt bridge with D398 of PPAT (as shown in Figure 2d and Figure 4f).

We have reworded this to reflect that L217A and K218A are the sole residues in NUDT5 that directly contact the AMP glue.

4. Line 459 (ED Figure 5d legend). "W26A/W48A" should be "W28A/W46A". The design rationale of this mutant, by the way, is not introduced in either the main text or the ED Figure 5 legend.

We have fixed the residue labeling error and elaborated on this mutant in the ED Figure 5 legend. It is redundant with E112Q/E116Q (shown in main figure) as a catalytically inactive NUDT5 mutant that is deficient in ADP-ribose binding.

5. ED Figure 4e, 4i. Three key PPAT contacting residues in NUDT5, namely Y74, F216, and F219 are not conserved in fish. Does this mean that the PPAT inhibitory function of NUDT5 is lost in fish?

The residues in NUDT5 that contact PPAT have an interesting evolutionary trajectory. These motifs appear to take form during the sea to land transition as they are present in xenopus but largely missing in fish, suggesting that this regulation of PPAT is a more recent adaptation. Understanding the evolutionarily origin and physiological context for this adaptation will certainly be exciting for future studies, but we currently have no reason to believe that fish PPAT is regulated by the mechanism described here for human cells.

Referee 4:

I co-reviewed this manuscript with one of the reviewers who provided the listed reports.

We would like to thank this reviewer for providing comments on our manuscript!

Referee 5:

The study by Witus et al. indicates that endogenous small-molecule glues play an important role in the regulation of metabolism. Given that exogenous small-molecule glues have been known for some time to enhance weak, preexisting protein-protein interactions, it is intuitive that human endogenous small molecules would function in the same manner. Witus et al. convincingly demonstrate that this is indeed the case, uncovering a regulatory mechanism that shows how purine nucleotide levels feed back on and restrict de-novo purine biosynthesis and, moreover, reveals an unexpected aspect of the mode-of action of chemotherapeutics that have been in use for more than half a century.

In addition to presenting a first example for a human endogenous small-molecule glue, this study significantly advances our understanding of how de novo purine biosynthesis and its regulation in response to purine availability. The structural and biochemical studies performed by the authors convincingly demonstrate that purine nucleotides promote the interaction of PPAT with NUDT5 to inhibit PPAT activity and limit de novo purine biosynthesis.

To my knowledge, this is the first report of an endogenous human metabolite family stabilizing a native protein-protein interaction through a molecular glue-like mechanism – there may be other examples for weak stabilization of P-P-interactions by metabolites, but what distinguishes the example presented here is that (i) the endogenous purine nucleotides acting as glues enhance P-P-interaction by almost two orders of magnitude, and (ii) that affinity of the pocket the purine nucleotides bind to when part of the P-P complex is compatible with the cellular concentrations of purine nucleotides, demonstrating biological relevance.

Taken together, the findings reported in this manuscript are of high significance for three reasons, because they (i) expand our understanding of how endogenous metabolites can regulate protein function in humans, (ii) demonstrate a feedback mechanism that regulates purine biosynthesis, and (iii)

provides strong evidence for an unexpected aspect of the mode of action for widely used chemotherapeutics.

We thank this reviewer for having received such positive comments!

However, I feel that the authors should be cautious not to overinterpret their data by extrapolating too much – I agree that it is likely that metabolic glues (I'm not particularly fond of this term, how about "metabolite glues") play a role in the regulation of many other metabolic and signaling pathways in diverse organisms. But I do not think there is enough data to predict (as the authors do, e.g., in line 319) that a high degree of structural flexibility is a general feature of metabolic glue pockets, as opposed to glue pockets of non-endogenous compounds. The current study simply does not provide sufficient evidence to make this claim, as it describes essentially one single example. It could certainly be that endogenous small-molecule glues bind to generally more flexible pockets than non-endogenous glues, but this can't be concluded based on the data presented here. Rather, it appears that the relative flexibility of this particular pocket is adapted to the diversity of purine metabolites present in the cell, and takes advantage of the fact that metabolites from this pathway - consisting of a polar, acidic phosphorylated ribose attached to a less polar, aromatic moiety - exhibit some general structural commonalities. Other pathways may have evolved to sense one specific metabolite, representing a key intermediate with characteristic (and perhaps otherwise uncommon) structural features, and correspondingly, the relevant binding pocket may exhibit much less flexibility, more similar to known non-endogenous glues.

We appreciate this reviewer's concern, and we have changed the description to "metabolite glues" as suggested. We agree that while we demonstrate structural plasticity in the glue pocket that enables accommodation of multiple endogenous and therapeutic glues, it is possible that other glue complexes show less flexibility in their binding pockets. As such, we have re-worded our discussion to be more specific to PPAT-NUDT5, while stipulating that this may be a more general feature of protein complexes to be borne out with more examples.

Minor comments:

- I'm surprised the authors did not actually attempt to demonstrate that the R70-Y74 interface is likely broken by phosphorylation of Y74. It would seem that the team has all the necessary tools in hand. This is not critical, but would round the narrative by providing insight in how regulation by metabolite glues intersects with phosphorylation-based cascades to regulate purine biosynthesis.

While phosphorylation of NUDT5 Y74 has been reported in the PhosSitePlus database in multiple cell types (**Fig. 1g**, link to database), the responsible kinase has not been identified. Thus, full reconstitution is at this point

not possible. To address this reviewer's concern, we have used DIA mass spectrometry to detect phosphorylation of Y74 of NUDT5 **[new Fig. 1i]**. We can indeed detect NUDT5-Y74 phosphorylation and document specificity of our mass spectrometry-based approach, as phosphorylation is lost by mutation of Y74 ($NUDT5^{Y74F}$). Importantly, we did not detect any Y74-phosphorylation when NUDT5 was bound to PPAT, as revealed by DIA mass spectrometry of sequential PPAT-NUDT5 immunoprecipitations. While defining upstream signaling pathways responsible for Y74 phosphorylation would be beyond the scope of a manuscript, these results validate our structures and deepen mechanistic insight of this complex by showing that modification of Y74 in NUDT5 is incompatible with formation of a stable PPAT-NUDT5 complex.

- In the last part of the Results section, it seems unnecessary to first discuss 6-benzyl and then 6-ethyl-TIMP, in two separate paragraphs. This section should be condensed.

We have added additional data that expands on the functional significance of 6-ethylTIMP in enhancing on-target cytotoxicity in cells compared to 6-meTIMP **[new Fig. 5e]**. We hope that the reviewer agrees that our additional data now warrants an explicit description of the 6-ethylTIMP results.

- The legends to Figures 1b-e lack necessary detail. For example, the color coding in 1b is not explained – one can infer indirectly (looking at panel 1d) that the colors relate to the role of the proteins in purine or pyrimidine biosynthesis, but this should be explicitly stated in the legend (the insert in the lower right of 1b is not a substitute, as is this merely adds to the confusion). Also, the description of the genetic screen in the main text is too abbreviated – it should be possible for a non-expert to understand what was done, without having to resort to references or the method section.

We apologize for this oversight and have added details pertaining to colors to the legend, and we have added additional details to better explain the significance of the CRISPR screen.

line 301: "prevent excessive use of metabolic building blocks." If I understand the authors' intent correctly, the point is that de novo purine biosynthesis, i.e., misallocation of metabolic resources, is prevented.

Yes, this improves the clarity of our point. We have re-worded this statement accordingly.

line 49: "amino acids, or ribose-5-phosphate" should be "amino acids, and ribose-5-phosphate"

We have corrected this mistake.

line 100: extraneous “how”.

We have corrected this mistake as well.

Figure 3c and elsewhere – p-values should be added where appropriate.

We have added statistical tests for all key cellular and biochemical data.

Figure 4a: It should be indicated that the PPAT activity assay panels compare benzyl- (top) and ethyl- (bottom) derivatives with 6-meTIMP. Also, please confirm whether the same data for 6-meTIMP are shown in the top and bottom panels.

Yes, the 6-meTIMP data are the same in both panels. All data was collected at the same time and separated for clarity. We have now explicitly noted this in the figure legend. We have also noted this in all locations where data that were collected at the same time are displayed in different panels/figures together.

Extended Data Figure 2: If this is from modeling the monomer interaction, this should be covered in the Methods (the current AlphaFold section describes modeling of the 4-8 complex).

We have updated the Methods to include all AlphaFold modeling schemes.

Methods:

Line 879: when calculating M+2, was the contribution from the 2x13C peak removed?

For de novo purine synthesis measurements in [^{15}N-amide]-L-glutamine labeled samples, all raw peak areas were corrected for the natural abundance of ^{15}N, ^{13}C, ^{18}O, and ^{2}H isotopes using IsoCorrectoR (version 3.22). This detail is now included in the methods section.

.**Line 874: “area … were”” singular-plural mismatch.**

This is now corrected

Line 888: “2uL” – should be microliter

This is now corrected

Referee #5 (Remarks to Authors):

In their revision the authors added substantial new data demonstrating assembly of the PPAT-NUDT5 complex in vitro and in cells under different nutrient conditions, including nutrient-dependent regulation of complex formation in cells. Further, the authors now include mass spectrometric data that confirm phosphorylation at Y74 of NUDT5 [new Fig. 1i]. These new results further support the authors' model for feedback regulation of purine biosynthesis and demonstrate its physiological relevance.

Thank you for the positive feedback on our additional data!

Myself and several other reviewers had taken issue with the authors' conjecture that a high degree of structural flexibility is a general feature of metabolic glue pockets. In the revised discussion, the authors have toned down their claims somewhat, but the wording could still be somewhat more careful (please see suggested edits below).

Thank you for pointing this out. We will implement the suggested edits (addressed below).

Major point:

There is one additional point that I missed in my initial review and that, however, I think is important to address: Figure 1b shows that sgRNAs for enzymes that divert IMP from AMP production to GMP production, especially IMPDH2, are protective, as opposed to enzymes in the mainstem purine biosynthetic pathway. In fact, IMPDH2 is more protective than NUDT5. How does that fit with the model presented here? Is there any evidence that the AMP-to-GMP ratio feeds back in some way on purine biosynthesis, e.g., via PPAT? If the role of IMPDH2 cannot explained at this stage, it would be essential to comment on this in the Discussion, as an observation that the current model cannot yet explain.

Thank you so much for pointing this out! While an interesting result, it is important to remember that the screen described in **Figure 1b** investigated genetic interactions between broad

inhibition of all Cullin-RING E3 ligases by MLN4924 and metabolic enzymes. The most direct explanation for IMPDH2 scoring as a protective hit would be its regulation by an E3 ligase, rather than purine sensing being affected by the AMP-GMP ratio. So far, we have not been successful in disentangling all links between purine synthesis and E3 ligases, but previous studies had suggested that IMPDH2 is under control of an enzyme called CUL5-ANKRD9 (link to study; to be added to citations in text). This citation had been missing from our paper, and we are very grateful that this was pointed out by this comment.

Although it is possible that the AMP-GMP ratio may affect purine sensing, we would like to point out GMP (as well as IMP) also acts as a molecular glue of the PPAT-NUDT5 complex. This further suggests that the genetic interaction between IMPDH2 depletion and MLN4924 treatment stems from the blocked activity of CUL5-ANKRD9 (or another yet unknown E3 ligase). Clearly, these are very interesting observations that clearly warrant additional study, but we hope the reviewer agrees that they extend beyond the scope of our current work.

Minor points:

- the last two paragraphs of the Results section are still poorly integrated. The importance of the large hydrophobic pocket for accommodating 6-benzylTIMP is explicitly discussed in lines 293-302, but then the following paragraph again starts by stating that there is a hydrophobic pocket. This last paragraph should be condensed significantly.

In the first paragraph mentioned by the reviewer we note that the benzyl substituent reorients the PPAT hydrophobic pocket compared to 6-meTIMP. This is supported by a unique cryo-EM structure of PPAT-NUDT5-6-benzylTIMP complex where we see disruption to the local conformation of PPAT **(Figure 5c, d)**. In the second paragraph we stipulate that the intact hydrophobic pocket may be harnessed to increase the *in vitro* potency of thiopurine glues, as is observed with 6-ethylTIMP. As these experiments are probing different phenomena of the two thiopurine analogs, we believe that this deserves independent discussion in the results section. We hope that this is ok.

- Line 322 "Metabolite glues restrict de novo purine..". Not all metabolite glues will do this. What is meant is that "The identified metabolite glues restrict de novo purine…".

This will be changed to the reviewer's suggestion in the final version.

- Line 325: "endogenous molecular glues serve crucial regulatory functions". I think "can serve" would be more appropriate.

This will be changed to the reviewer's suggestion in the final version.
